# Well-posedness of Keller–Segel systems on compact metric graphs

**DOI:** 10.1007/s00028-024-01033-x

**Published:** 2024-12-15

**Authors:** Hewan Shemtaga, Wenxian Shen, Selim Sukhtaiev

**Affiliations:** Department of Mathematics and Statistics, Auburn University, Auburn, AL 36849 USA

**Keywords:** Neumann–Kirchhoff Laplacian, Heat semigroup, Chemotaxis, Quantum graphs, 35Q92, 92C17, 35P05

## Abstract

Chemotaxis phenomena govern the directed movement of microorganisms in response to chemical stimuli. In this paper, we investigate two Keller–Segel systems of reaction–advection–diffusion equations modeling chemotaxis on thin networks. The distinction between two systems is driven by the rate of diffusion of the chemo-attractant. The intermediate rate of diffusion is modeled by a coupled pair of parabolic equations, while the rapid rate is described by a parabolic equation coupled with an elliptic one. Assuming the polynomial rate of growth of the chemotaxis sensitivity coefficient, we prove local well-posedness of both systems on compact metric graphs, and, in particular, prove existence of unique classical solutions. This is achieved by constructing sufficiently regular mild solutions via analytic semigroup methods and combinatorial description of the heat kernel on metric graphs. The regularity of mild solutions is shown by applying abstract semigroup results to semi-linear parabolic equations on compact graphs. In addition, for logistic-type Keller–Segel systems we prove global well-posedness and, in some special cases, global uniform boundedness of solutions.

## Introduction

### Overview

Biological species often exhibit tendency to drift, spread or localize within complex media subject to external signals. This phenomenon plays a vital role in formation, ordering, and stability of patterns observed experimentally and numerically in models of bio-engineering (dermal tissue recovery [42]), ecology (population dynamics [44]), and social sciences (e.g., urban crime dynamics [45, 46]). The external signals and cues in question are generated by an attractant or a repellent facilitating the migration. In the biological context, the directed movement of bacteria, cells or other microorganisms in response to chemical attractant is often referred to as chemotaxis, which is a widely studied phenomenon in pure and applied mathematics [4, 11, 13, 27, 28, 32, 33, 41, 48, 56, 57, 59, 63, 65–71, 73, 74].

The two quantities central to the mathematical modeling of such processes are the density of biospecies *u*(*t*, *x*) and the concentration of attracting (repelling) substance *v*(*t*, *x*). Their coupled evolution is described by the Keller–Segel model, that is, by a pair of nonlinear partial differential equations stemming from the diffusion equations

1.1 $$\begin{aligned} {\left\{ \begin{array}{ll} \partial _tu+\partial _ xJ_{u,v}-\varphi (u,v)=0,\\ \tau \partial _tv+\partial _ x J_v-\psi (u,v)=0,\\ \end{array}\right. } \end{aligned}$$

where

- $J_{u,v}=-\partial _x u+\chi (u,v)\partial _xu$ is the density flux which consist of the taxis-flux term $\chi (u,v)\partial _xv$ that governs the population drifts in response to attractant *v* and the standard flux term $-\partial _x u$ given by Fick’s law,
- $J_v= - \partial _x v$ is the standard flux term describing diffusion of the chemo-attractant by Fick’s law; importantly, in the case of rapid diffusion, that is, $0<\tau \ll 1$ the second equation (1.1) can be approximated by $\partial _{xx}^2 v-\psi (u,v)=0$,
- $\varphi (u,v)$, $\psi (u,v)$ describe the rates at which the biospecies, respectively, the chemo-attractant are produced.

In this paper, we provide a systematic treatment of general chemotaxis systems with moderate and fast rates of diffusion of the attractant on arbitrary compact metric graphs. Concretely, we focus on the following two systems of reaction–advection–diffusion equations of parabolic-parabolic type

1.2 $$\begin{aligned} {\left\{ \begin{array}{ll} \partial _tu=\partial _ x(\partial _x u-\chi (u,v)\partial _x v)+\varphi (u,v),\\ \tau \partial _tv=\partial _ {xx}^2 v+\psi (u,v),\\ \end{array}\right. } \end{aligned}$$

and parabolic-elliptic type

1.3 $$\begin{aligned} {\left\{ \begin{array}{ll} \partial _tu=\partial _ x(\partial _x u-\chi (u,v)\partial _x v)+\varphi (u,v),\\ 0=\partial _ {xx}^2 v-\sigma v +\psi (u), \\ \end{array}\right. } \end{aligned}$$

with nonlinear terms $\chi , \varphi , \psi$ that exhibit polynomial growth conditions at infinity and vanish to the first order at zero, see Hypothesis 1.1 for more details.

### Description chemotaxis models on graphs

Throughout this paper we assume that ${\Gamma }=(\mathcal {V}, \mathcal {E})$ is a compact metric graph. That is, the set of vertices $\mathcal {V}$ is finite, the set edges $\mathcal {E}$ is finite and consists of edges of finite length. We assume that ${\Gamma }$ is a connected and oriented graph, that is, each edge *e* is equipped with the initial vertex $i(e)\in \mathcal {V}$ and the terminal vertex $t(e)\in \mathcal {V}$. An edge $e\in \mathcal {E}$ is identified with the interval (0, |*e*|) so that every point on the graph is described by a pair of coordinates $(e, \xi )$, $\xi \in (0,|e|)$. A function $u:\Gamma \rightarrow {\mathbb {R}}$ (resp. $u:{\bar{\Gamma }}\rightarrow {\mathbb {R}}$) is given by a collection of functions $\{u_e| e\in \mathcal {E}\}$ where $u_e:(0,|e|)\rightarrow {\mathbb {R}}$ (resp. $u_e:[0,|e|]\rightarrow {\mathbb {R}}$). Differentiation and integration are defined edge-wise with respect to the chosen orientation of edges, that is, $\partial _x u({ e,x}){:}{=}\partial _x u_e(x),$ $x\in (0,e)$ and $\int _{\Gamma } u(x)dx{:}{=} \sum \nolimits _{e\in \mathcal {E}} \int _0^{|e|} u_e(x)dx.$ Throughout this paper, $\partial _{\nu } u_e{(\vartheta )}$ (with $\vartheta =(e,0)$ or $\vartheta =(e,|e|)$) denotes the inward pointed normal derivative of *u* at the end points of the edge *e*, i.e.,

1.4 $$\begin{aligned} \partial _{\nu }u (\vartheta )={\left\{ \begin{array}{ll} u_e'(0)&  \vartheta =({e,0}),\\ -u_e'(|e|)&  \vartheta =({ e, |e|}). \end{array}\right. } \end{aligned}$$

The functional spaces are often considered as direct sums of the corresponding functional spaces on individual edges. That is, if $u_e\in \mathcal {X}(0, |e|)$ for all $e\in \mathcal {E}$ then we write $u\in \widehat{\mathcal {X}}(\Gamma )$ where $\mathcal {X}$ is one of the functional spaces $C_0^{\infty }$, *C*, $C^{\nu }$, $L^p$, $W^{k,p}$.

The class of chemotaxis models under investigation is parameterized by a scalar $\tau \ge 0$ and given by

1.5 $$\begin{aligned} {\left\{ \begin{array}{ll} u_t=\partial _x\big (\partial _{x} u-f_1(u,v)\partial _xv\big )+f_2(u,v), \quad {t>0,\,\, x\in \Gamma ,}\\ \tau v_t=\partial _{xx}^2 v+f_3(u,v), \quad { t>0,\,\, x\in \Gamma ,}\\ u(0, x)=u_0(x),\ v(0,x)=v_0(x),\quad { x\in \Gamma ,} \\ \end{array}\right. } \end{aligned}$$

where the functions

$$\begin{aligned} u:[0,\infty )\times {\overline{\Gamma }}\rightarrow {\mathbb {R}}, v:[0,\infty )\times {\overline{\Gamma }}\rightarrow {\mathbb {R}}, \end{aligned}$$

are subject to natural Neumann–Kirchhoff vertex conditions for $t>0$, that is, they obey

1.6 $$\begin{aligned}&\text {continuity at vertices: }u_e( t,\vartheta )=u_{e'}({ t,} \vartheta ),\ { v_e( t, \vartheta )=v_{e'}({t,}\vartheta )},\ \vartheta \sim e, e', \end{aligned}$$

**Fig. 1:**
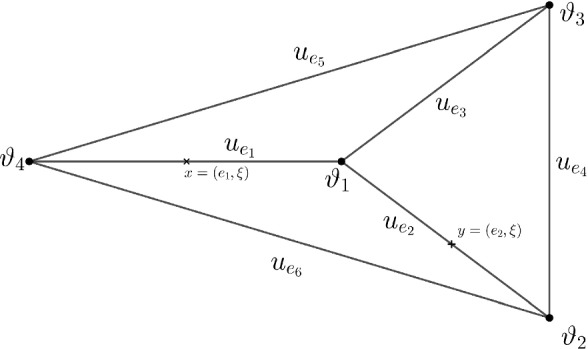
A metric graph with 6 edges and 4 vertices. A function *u* is given by a vector $u_{e_1},..., u_{e_6}$

1.7 $$\begin{aligned}&\text {current conservation: }\sum _{e\sim \vartheta }\partial _{\nu }u_e({ t,}\vartheta )=0, \,\,\, \ { \sum _{e\sim \vartheta }\partial _{\nu }v_e({t,} \vartheta )=0}, \ \end{aligned}$$

here and throughout the paper $\vartheta \sim e$ means that edge *e* is incident with vertex $\vartheta$, see Fig. 1, where, for example, $\vartheta _1\sim e_k$, $k=1,2,3$. The first condition (1.6) guarantees continuity of *u* over the graph $\Gamma$ considered as a metric space. The second condition (1.7) yields preservation of current through the vertex, and, in particular, facilitates preservation of total mass for the minimal model, i.e., $f_2, f_3\equiv 0$.

The distinction between two regimes $\tau =0$ and $\tau >0$ corresponding to parabolic–elliptic and parabolic–parabolic PDEs, respectively, plays an important role throughout the paper. The nonlinear terms $f_1, f_2, f_3$ under consideration satisfy the following assumptions.

#### Hypothesis 1.1

Let $f_k:{\mathbb {R}}^2\rightarrow {\mathbb {R}}$ and denote

1.8 $$\begin{aligned} g_1(s,{ r}):=\,s^{-1}f_1(s,{ r}), g_2(s,{r}):= s^{-1}f_2(s,{ r}), g_3(s,{ r}):=\,s^{-1}\partial _{ r}f_1(s,{ r}). \end{aligned}$$

For $k=1,2,3$, assume that $f_k, g_k\in C^{1,1}({\mathbb {R}}^2)$ and that

1.9 $$\begin{aligned} \sum _{\begin{array}{c} |\alpha |\le 1\\ 1\le k\le 3 \end{array}}|D^{\alpha } f_k(s,{ r})|+|D^{\alpha } g_k(s,{ r})| \le C(1+|s|^{\mu _1}+|{ r}|^{\mu _2}),\ (s,{ r})\in {\mathbb {R}}^2, \end{aligned}$$

for some $C=C(f_1, f_2, f_3, \mu _1, \mu _2)>0$, $\mu _1, \mu _2\ge 0$ with $\gamma {:}{=}\mu _1+\mu _2\ge 1$.

If $\tau =0$ assume that $f_3(u,v)=-\sigma v+f(u)$ with $\sigma >0$. Finally, throughout this paper we assume that $p> 8\gamma$.

#### Remark 1.1

The polynomial growth assumption (1.9) is used to prove various $L^p-L^q$ mapping properties of the nonlinear operators of the type $(u,v)\mapsto D^{\alpha }(f(u,v)D^{\beta }v)$ that naturally stem from the right-hand side of (1.5), see, for example, Propositions 4.1, 4.2 and Appendix B. A typical illustration of how condition (1.9), specifically the polynomial growth bound for $f_k$ ($k=1,2,3$), is used in this direction is given by the inequality

1.10 $$\begin{aligned} \int _{\Gamma }\left| f_k(u,v)\right| ^{\frac{p}{\gamma }}dx&\lesssim \int _{\Gamma }\big (1+|u|^{\frac{\mu _1 p}{\gamma }}+|v|^{\frac{\mu _2 p}{\gamma }}\big )dx\nonumber \lesssim (1+\Vert u\Vert _{L^p(\Gamma )}^p+\Vert v\Vert _{L^p(\Gamma )}^p), \end{aligned}$$

which gives the boundedness of the nonlinear mapping $L^{p}(\Gamma )\times L^{p}(\Gamma )\ni (u_1, u_2)\mapsto f_k(u,v)\in L^{\frac{p}{\gamma }}(\Gamma )$ ($k=1,2,3$). In a similar fashion, the condition (1.9), specifically polynomial growth bound for first partial derivatives of $f_k$, combined with the fundamental theorem of calculus yields local Hölder continuity of this mapping, cf. (B.6)–(B.9) with $F=f_k$. The polynomial growth bound for $g_k$ is used to infer Hölder continuity of the mappings *a*, *b* in (4.12) which naturally appear in the auxiliary non-autonomous linear system (4.10), (4.11).

This level of generality includes, in particular, the following models of chemotaxis: the minimal model [11, 27], the density and signal dependent sensitivity models [27, (M2b), (M3a)], the cell kinetics model [27, (M8)], the nonlinear diffusion [17] and secretion [72] models as well as some others, cf. [2, Table 1].

### Main results

Our first main result concerns well-posedness of the chemotaxis model on compact metric graphs in two regimes: $\tau =0$ and $\tau >0$. In the following, $\mathcal {X}^{\alpha }_p$ ($\alpha \in (0,1)$, $1\le p< \infty$) denotes the fractional power spaces generated by the Neumann–Kirchhoff Laplacian on $\widehat{L}^p(\Gamma )$. We provide a more detailed discussion of functional spaces on graphs in Appendix A.^1^

#### Theorem 1.1

Assume Hypothesis 1.1 and fix arbitrary $r\ge 1$, $\beta \in (0,1/8)$, $\nu <\beta$.

1. Assume that $\tau =0$. Then for arbitrary $u_0\in \widehat{L}^p(\Gamma )$ there exists $T_{\max }=T_{\max }(u_0)\in (0,\infty ]$ such that (1.5) has a unique classical solution $(u(t,x),v(t,x))=(u(t, x; u_0),v(t, x; u_0))$, $t\in [0, T_{\max })$ satisfying the Neumann–Kirchhoff vertex conditions (1.6), (1.7) for $t>0$, $u,v\in \widehat{C}^{1,2}( (0, T_{\max })\times \overline{\Gamma })$, and
  1.11 $$\begin{aligned} u\in C^{{\beta }}((0, T_{\max }), \widehat{C}^{\nu }(\overline{\Gamma }))\cap C([0, T_{\max }), \widehat{L}^p(\Gamma ))\cap C^{\beta }((0, T_{\max }), \mathcal {X}^{\beta }_r). \end{aligned}$$
  If $T_{\max }<\infty$ then $\limsup \limits _{t\rightarrow T_{\max }^-}\Vert u(t,\cdot )\Vert _{{ L^p(\Gamma )}}=\infty$. Finally, if $u_0\ge 0$, then *u*, *v* are non-negative as well.
2. Assume that $\tau >0$ and let $(u_0, v_0)\in \widehat{L}^p(\Gamma )\times \widehat{W}^{1, p}(\Gamma )$. Then there exists $T_{\max }=T_{\max }(u_0, v_0)\in (0,\infty ]$ such that (1.5) has a unique classical solution
  1.12 $$\begin{aligned} (u(t,x),v(t,x))=(u({ t,x}; u_0, v_0),v({ t,x}; u_0, v_0)), t\in [0, T_{\max }), \end{aligned}$$
  satisfying the Neumann–Kirchhoff vertex conditions (1.6), (1.7) for $t>0$, $u,v\in \widehat{C}^{1,2}( (0, T_{\max })\times \overline{\Gamma })$, and
  1.13 $$\begin{aligned} u&\in C([0, T_{\max }), \widehat{L}^p(\Gamma ))\cap C^{\beta }((0, T_{\max }), \mathcal {X}^{\beta }_r)\cap C^{\beta }((0, T_{\max }), \widehat{C}^{\nu }(\overline{\Gamma })), \end{aligned}$$
  1.14 $$\begin{aligned} v&\in C([0,T], \widehat{L}^p(\Gamma ))\cap C^{1+\beta } ((0,T_{\max }), \mathcal {X}^{\beta }_r)\cap C^{\beta }((0, T_{\max }), \widehat{C}^{2+\nu }(\overline{\Gamma })). \end{aligned}$$
  If $T_{\max }<\infty$ then $\limsup \limits _{t\rightarrow T_{\max }^-}\Vert (u(t,\cdot ),v(t,\cdot ))\Vert _{L^p(\Gamma )\times \widehat{W}^{1, p}(\Gamma )}=\infty .$ Finally, if $u_0\ge 0, v_0\ge 0$, then *u*, *v* are non-negative.

Our second principal result concerns global existence of unique classical solutions for logistic-type models given by

1.15 $$\begin{aligned} {\left\{ \begin{array}{ll} u_t=\partial _x\big (\partial _{x} u-\chi u\partial _xv\big )+g(u),\quad { t>0,\,\, x\in \Gamma ,} \\ \tau v_t=\partial _{xx}^2 v-v+u, \quad { t>0,\,\, x\in \Gamma ,}\\ u(0, x)=u_0(x),\ v(0,x)=v_0(x),\quad {x\in \Gamma }\\ \end{array}\right. } \end{aligned}$$

subject to the Neumann–Kirchhoff vertex conditions (1.6), (1.7) for $t>0$.

#### Theorem 1.2

Let $g: {\mathbb {R}}\rightarrow {\mathbb {R}}$ and $\widetilde{g}(s){:}{=}s^{-1}g(s)$. Assume that $g, \widetilde{g}\in C^1({\mathbb {R}})$ and suppose that for some constants $C>0$, $k\ge 0$, $l\ge 0$, $\gamma \ge 1$ one has

1.16 $$\begin{aligned}&|g(s)|+|g'(s)|+|\widetilde{g}(s)|+|\widetilde{g}'(s)|\le C(1+|s^{\gamma }|), \end{aligned}$$

1.17 $$\begin{aligned}&g(s)\le k+ls,\text { for all } s\ge 0. \end{aligned}$$

(1) Assume that $\tau =0$. Let $u_0\in \widehat{L}^p(\Gamma )$ with $u_0\ge 0$. Then the chemotaxis system (1.14) has a unique global non-negative classical solution $(u(t,x),v(t,x))=(u(t,x; u_0),v(t,x; u_0))$, satisfying the Neumann–Kirchhoff vertex conditions (1.6), (1.7) for $t>0$, $u,v\in \widehat{C}^{1,2}( (0, \infty ) \times \overline{\Gamma })$. Suppose, in addition, that for some $m\ge 0$ and $\varepsilon >0$ one has

1.18 $$\begin{aligned} g(s)\le k+ls-m s^{1+\varepsilon },\text { for all } s\ge 0. \end{aligned}$$

If either $m>0$ or $k=l=m=0$ then the solution is globally uniformly bounded, that is,

1.19 $$\begin{aligned} \sup _{t\ge 0}\Vert u(t, \cdot )\Vert _{\widehat{L}^{\infty }(\Gamma )}<\infty . \end{aligned}$$

(2) Assume that $\tau >0$. Let $(u_0, v_0)\in \widehat{L}^p(\Gamma )\times \widehat{W}^{1, p}(\Gamma )$, $u_0\ge 0, v_0\ge 0$. Then the chemotaxis system (1.14) has a unique global non-negative classical solution

1.20 $$\begin{aligned} (u(t,x),v(t,x))=(u(t,x; u_0,v_0),v(t,x; u_0,v_0)), u,v\in \widehat{C}^{1,2}( (0, \infty )\times \overline{\Gamma }), \end{aligned}$$

satisfying the Neumann–Kirchhoff vertex conditions (1.6), (1.7) for $t>0$. Suppose, in addition, that *g* satisfies (1.17). If either $m>0$ or $k=l=m=0$ then the solution is globally uniformly bounded, that is,

1.21 $$\begin{aligned} \sup _{t\ge 0}\left( \Vert u(t, \cdot )\Vert _{\widehat{L}^{\infty }(\Gamma )}+\Vert v(t, \cdot )\Vert _{\widehat{L}^{\infty }(\Gamma )}\right) <\infty . \end{aligned}$$

Theorem 1.1 is proved by showing the existence, uniqueness, and regularity of mild solutions of (1.5) subject to (1.6), (1.7). Theorem  1.2 is proved via the boundedness of $\Vert u(t,\cdot )\Vert _{\widehat{L}^q(\Gamma )}+\Vert v(t,\cdot )\Vert _{\widehat{L}^q(\Gamma )}$ for all $q\ge 1$. The proofs of the existence, uniqueness, and regularity of mild solutions^2^ of (1.5) subject to (1.6), (1.7), and the boundedness^3^ of $\Vert u(t,\cdot )\Vert _{\widehat{L}^q(\Gamma )}+\Vert v(t,\cdot )\Vert _{\widehat{L}^q(\Gamma )}$ heavily rely on various quantitative and qualitative properties of the analytic semigroup $e^{\Delta t}$ generated by Neumann–Kirchhoff Laplacian and the evolution operator generated by linear non-autonomous parabolic equations on metric graphs. One of the important results along this line is an $L^p-W^{1, q}$ bound for the norm of the heat semigroup, which we present next.

#### Theorem 1.3

Let $\{e^{\Delta t}\}_{t\ge 0}$ be analytic semigroup generated by the Neumann–Kirchhoff Laplacian in $\widehat{L}^p(\Gamma )$, $1\le p<\infty$. Let $q\in [p, \infty )$ and $T>0$. There exists a constant $C=C( T, p, q, \Gamma )>0$ such that the following statements hold.

1. For arbitrary $u\in C_0^\infty (\Gamma )$ and ${t\in (0, T]}$ one has
  1.22 $$\begin{aligned}&\Vert e^{\Delta t}\partial _x u\Vert _{\widehat{L}^q(\Gamma )}\le Ct^{-\frac{1}{2}-\frac{1}{2}\left( \frac{1}{p}-\frac{1}{q} \right) }\Vert u\Vert _{\widehat{L}^p(\Gamma )}, \end{aligned}$$
  1.23 $$\begin{aligned}&\Vert \partial _xe^{\Delta t} u\Vert _{\widehat{L}^q(\Gamma )}\le Ct^{-\frac{1}{2}-\frac{1}{2}\left( \frac{1}{p}-\frac{1}{q} \right) }\Vert u\Vert _{\widehat{L}^p(\Gamma )}. \end{aligned}$$
  In particular, the operators $e^{\Delta t}\partial _x$, $\partial _xe^{\Delta t}$ originally defined on $C_0^{\infty }(\Gamma )$ can be uniquely extended to bounded operators in^4^$\mathcal {B}(\widehat{L}^p(\Gamma ), \widehat{L}^q(\Gamma ))$ with
  1.24 $$\begin{aligned}&\Vert e^{\Delta t}\partial _x \Vert _{\mathcal {B}(\widehat{L}^p(\Gamma ), \widehat{L}^q(\Gamma ))}\le Ct^{-\frac{1}{2}-\frac{1}{2}\left( \frac{1}{p}-\frac{1}{q} \right) },\ {t\in (0, T]}, \end{aligned}$$
  1.25 $$\begin{aligned}&\Vert \partial _x e^{\Delta t} \Vert _{\mathcal {B}(\widehat{L}^p(\Gamma ),\widehat{L}^q(\Gamma ))}\le Ct^{-\frac{1}{2}-\frac{1}{2}\left( \frac{1}{p}-\frac{1}{q} \right) },\ {t\in (0, T]}. \end{aligned}$$
2. For arbitrary $u\in \widehat{L}^p(\Gamma )$, ${t\in (0, T]}$ one has
  1.26 $$\begin{aligned} ||e^{\Delta t}u||_{\widehat{L}^q(\Gamma )} \le C t^{-\frac{1}{2}(\frac{1}{p} -\frac{1}{q})}||u||_{\widehat{L}^p(\Gamma )}, \end{aligned}$$
  and
  1.27 $$\begin{aligned} \Vert e^{\Delta t} \Vert _{\mathcal {B}(\widehat{L}^p(\Gamma ), \widehat{W}^{1,q}(\Gamma ))}\le C\max \left\{ t^{-\frac{1}{2}-\frac{1}{2}\left( \frac{1}{p}-\frac{1}{q} \right) }, t^{{-\frac{1}{2}} \left( \frac{1}{p}-\frac{1}{q} \right) }\right\} . \end{aligned}$$

The analogous estimates for the heat semigroup on ${\mathbb {R}}^n$ and on bounded domains (with Neumann boundary conditions) have been obtained in [48] and [29], respectively. In the settings of non-compact metric graphs, the inequality (1.25) with $t\in (0, T]$ replaced by $t>0$ and $T-$independent constant *C* has been established in [3] for unbounded graphs.^5^ In this context, we also mention the estimates on the heat kernel discussed in [11, Proposition 2.2]. Our proof of Theorem 1.3 employs the combinatorial description of the heat kernel given, discussed, for example, in [3, 12], and integration by parts.

Another important object in the proof of Theorem 1.1 is the evolution operator for a general linear non-autonomous parabolic equation on a compact graph which is of independent interest.

#### Theorem 1.4

Let $0< \kappa <T$, $\sigma >0$, and $\psi _0\in \widehat{L}^q(\Gamma )$ for some $q\ge 1$. Assume that for some $\beta >0$ the mappings $[\kappa ,T)\ni t\mapsto a(t,\cdot )\in \widehat{L}^q(\Gamma )$ and $[\kappa ,T)\ni t\mapsto b(t,\cdot )\in \widehat{L}^q(\Gamma )$ belong to ${ C^\beta }\big ([\kappa , T), \widehat{L}^q(\Gamma )\big )$. Let us consider the non-autonomous parabolic equation on an arbitrary compact metric graph $\Gamma$

1.28 $$\begin{aligned} {\left\{ \begin{array}{ll} \partial _t\Psi =(\Delta -\sigma )\Psi +a(t,x)\partial _x\Psi +b(t,x)\Psi ,\ \, \, { t>\kappa ,}\,\, x\in \Gamma ,\\ \sum \limits _{\vartheta \sim e} \partial _{\nu }\Psi _e(t, \vartheta )=0,\ \Psi _e(t, \vartheta )=\Psi _{e'}(t, \vartheta ),\ e, e'\sim \vartheta ,\,\, t>\kappa \\ \Psi ({\kappa }, \cdot )=\psi _0(\cdot )\in \widehat{L}^q(\Gamma ). \end{array}\right. } \end{aligned}$$

Then the following assertions hold. For arbitrary $\gamma \in (0,1)$, $\tau \in (\kappa , T)$ and some $\varepsilon >0$ there exists a unique ${ \Psi (t)= \Psi (t; \psi _0,\kappa )}$ such that

1.29 $$\begin{aligned}&\Psi \in C((\kappa , \tau ], {\widehat{C}^{\varepsilon }}(\overline{\Gamma }))\cap C([\kappa , \tau ], L^{q}(\Gamma )),\,\, \Psi (t)\in \mathcal {X}^{\gamma }_q\,\, \textrm{for}\,\, t\in (\kappa ,\tau ],\nonumber \\&\Psi (t)=e^{(\Delta -\sigma )(t-\kappa )}\psi _0+\int _{\kappa }^{t}e^{(\Delta -\sigma )(t-\kappa -s)}\nonumber \\&\qquad \left( { a( s},\cdot )\partial _x\Psi { (s)}+{ b( s},\cdot )\Psi {(s)}\right) ds,\ t\in [\kappa , \tau ], \end{aligned}$$

where the integral converges in $\widehat{L}^q(\Gamma )$. Moreover, for $t\in (\kappa ,\tau ]$ the evolution operator $T(t,\kappa )$ defined by

2.1 $$\begin{aligned} T(t,\kappa ): L^q(\Gamma )\rightarrow \mathcal {X}^{\gamma }_q,\ T(t,\kappa )\psi _0={\Psi (t;\psi _0,\kappa )}, \psi _0\in \widehat{L}^q(\Gamma ), \end{aligned}$$

is bounded, i.e., $T(t,\kappa )\in \mathcal {B}(\widehat{L}^q(\Gamma ), \mathcal {X}^{\gamma }_q)$, with $\Vert T(t,\kappa )\Vert _{\mathcal {B}(\widehat{L}^q(\Gamma ), \mathcal {X}^{\gamma }_q)}\le C(t-\kappa )^{-\gamma }.$

In addition, if the mappings $t\mapsto a(t, \cdot ), b(t,\cdot )$ belong to ${ C^{\beta }}\big ([\kappa , T), \widehat{C}(\widehat{\Gamma })\big )$ then $\Psi (t,x)=\Psi (t,x;\psi _0,\kappa )$ defined by $\Psi (t,x;\psi _0,\kappa ){:}{=}\Psi (t;\psi _0,\kappa )(x)$ is a unique classical solution of the Cauchy problem (1.27) on the interval $[\kappa ,{ T)}$.

The two key features of the above result that play an important role in the sequel are boundedness of the evolution operator $T(t, \kappa )$ from $\widehat{L}^q(\Gamma )$ to $\mathcal {X}^{\gamma }_p$ for *arbitrary* $\gamma \in (0,1)$ and the explicit estimate on its norm. We refer the reader to the classical text [26] where a version of Theorem 1.27 is discussed for parabolic equations of the type $u_t=Au+B(t)u$ on abstract Banach spaces and the evolution operator is shown to act boundedly from $\mathcal {X}^{\alpha }_p$ to itself (whereas in our case, that is for special parabolic equation, we prove boundedness from $\mathcal {X}^{0}_p$ to $\mathcal {X}^{\gamma }_p$).

To conclude the introduction, let us provide a brief review of related recent results. The vast mathematical literature on chemotaxis models, Keller–Segel systems includes [4, 11, 18, 19, 24, 27, 28, 30–36, 41, 48, 49, 49–54, 56, 57, 64–70]. A large portion of these papers deals with versions of (1.5) posed on $\Omega \subseteq {\mathbb {R}}^d$, $d\ge 1$ with specific choices of nonlinear terms $f_1, f_2, f_3$ and boundary conditions (typically Neumann); often only one of the two cases, $\tau >0$ or $\tau =0$, is considered.

One of the main goals of the current work is to prove well-posedness of parabolic-parabolic and parabolic-elliptic Keller–Segel systems on metric graphs. Our motivation is threefold. First, such systems are widely used in bio-engineering models of dermal tissue reparation [22, 42, 55], where the propagation of fibroblasts (the cells responsible for reparation of dermal tissue) through artificial scaffolds (a network of fibers) is an important topic. In this context, the edges of the graph represent individual fibers whose crossings form the vertices of the model network. Second, in connection with the applied studies, the numerical analysis of the Keller–Segel model on networks has been used to demonstrate interesting dependence of the dynamics on the geometry of the graph [7, 9]. The current work naturally supplements the numerical studies with the rigorous proof of global well-posedness for rather general nonlinearities and completely general compact graphs. Our third motivation is to link a large body of work on elliptic differential operators on metric graphs see, for example, [5, 14–16, 20, 23, 37–40, 60, 61] to a rapidly developing theory of parabolic operators  [3, 8, 11, 21, 43] and evolution equations on metric graphs. The two papers on evolution equations that are especially closely related to ours are [3, 11]. In [11] the authors considered (1.5) with $f_1(u,v)=u$, $f_2(u,v)=0$, $f_3(u,v)=u-v$, $\tau \ge 0$ and constructed, via a fixed point argument, a mild solution of the parabolic-parabolic system in the space $L^{\infty }\left( (0, T); \widehat{C}(\Gamma )\right) \times L^{\infty }\left( (0, T); \widehat{W}^{1,\infty }(\Gamma )\right)$ and a global mild solution of the parabolic-elliptic system in $L^{\infty }\left( (0, \infty ); \widehat{C}(\Gamma )\right) \times L^{\infty }\left( (0, T); \widehat{C}^{2}(\Gamma )\right)$. In [11], the authors also provided a discussion (stemming from [47, 62]) of the heat kernel on compact graphs. An in-depth analysis of the heat semigroup, however, appeared in [3] where the authors established analyticity, ultracontractivity, and some heat kernel estimates all of which are employed in our work. We supplement the ultracontractivity estimate in [11, eq. (4.1)] by its analogue for the operator $e^{t(\Delta -\sigma )}\partial _x$, see Theorem 1.3. This is a key technical estimate that allows us to treat the chemotaxis sensitivity term $\partial _x(\chi (u,v)\partial _xv)$ which critically distinguishes the Keller–Segel model from the usual reaction-diffusion system.

**Outline of the paper.** In Sect. 2, we discuss the operator $e^{t\Delta }\partial _x$ as a mapping between $\widehat{L}^p(\Gamma )$ and ${\widehat{L}^q(\Gamma )}$ and prove Theorem 1.3. In Sect. 3, we study linear non-autonomous parabolic equations of the form (1.27) on metric graphs and prove Theorem 1.4. In Sects. 4 and 5, we give the proofs of Theorems 1.1 and 1.2 correspondingly, in particular, subsections 4.1, 5.1 contain brief outline of our proofs. In Appendix A, we recall fractional power spaces and interpolation theorems on metric graphs, in Appendix B we discuss $L^p-L^q$ mapping properties of nonlinear mappings arising in the right-hand side of (1.5), in Appendix C we summarize some important facts about general semi-linear parabolic equations on compact metric graphs. Throughout the rest of this paper, if no confusion occurs, we write $\widehat{L}^p(\Gamma )$ and $\widehat{L}^q(\Gamma )$ as $L^p(\Gamma )$ and $L^q(\Gamma )$, respectively.

## $L^p-L^q$ estimates for the operator $e^{\Delta t}\partial _x$ and proof of Theorem 1.3

In this section, we derive principal estimates on the norm of the operators $e^{\Delta t}$ and $e^{\Delta t}\partial _x$, which will be used to establish well-posedness of (1.5) subject to (1.6),(1.7), and prove Theorem 1.3.

We first recall from [3] the construction of the analytic semigroup or heat semigroup $e^{\Delta t}$. Let $\Delta$ be the Neumann–Kirchhoff Laplacian in $L^p(\Gamma )$, $1\le p<\infty$ defined as follows

2.2 $$\begin{aligned}&\Delta :\operatorname {dom}(\Delta )\subset L^p(\Gamma )\rightarrow L^p(\Gamma ),\ \Delta u:=\partial _x^2u,\ u\in \operatorname {dom}(\Delta ),\nonumber \\&\operatorname {dom}(\Delta ){:}{=}\left\{ u\in \widehat{W}^{2,p}(\Gamma ): \sum \limits _{\vartheta \sim e} \partial _{\nu }u_e(\vartheta )=0,\ u_e(\vartheta )=u_{e'}(\vartheta ), \vartheta \sim e, e'\right\} . \end{aligned}$$

For a given edge *e* we let $-e$ denote the same edge equipped with opposite orientation, i.e., $t(\pm e)=i(\mp e)$. The graph consisting of all edges $\pm e$ is denoted by $\widetilde{\Gamma }$. We say that edges $e, e'\in \mathcal {E}(\widetilde{\Gamma })$ are connected by a path *P* along $m\in {\mathbb {N}}_0$ edges whenever there exists $m+1$ edges $(e_0, e_1,..., e_m)\subset (\mathcal {E}(\widetilde{\Gamma }))^{m+1}$ with $e_0{:}{=} e$, $e_m{:}{=} e'$ and $t(e_k)=i(e_{k+1})$, $0\le k\le m-1$, the length of such path is dented by $|P|{:}{=}\sum _{k=0}^{m-1}|e_k|$. The collection of all paths connecting $e, e'$ along *m* edges is denoted by $\mathcal {P}_{e, e'}(m)$. The scattering coefficient matrix of size $2|\mathcal {E}|\times 2|\mathcal {E}|$ (here $|\mathcal {E}|$ denotes the number of edges of $\Gamma$) between edges $e, e'\in \mathcal {E}(\widetilde{\Gamma })$ is defined via

2.3 $$\begin{aligned} S_{e, e'}= {\left\{ \begin{array}{ll} \frac{2}{{ \deg (t(e))}}, & e\not =-e', t(e)=i(e'),\\ \frac{2}{{ \deg (t(e))}}-1, & e=-e', t(e)=i(e'),\\ 0, & \text { otherwise}, \end{array}\right. } \end{aligned}$$

where ${ \deg (t(e))}$ denotes the number of edges incident to *t*(*e*).

**Fig. 2:**
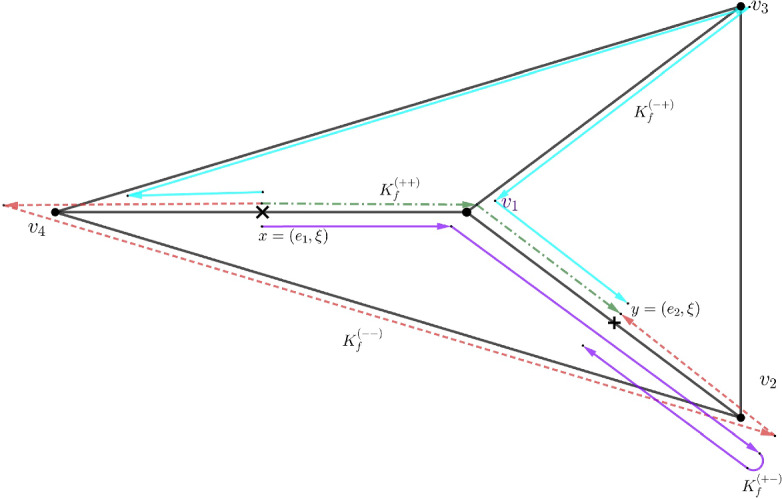
The orientation of edge $$e_1$$ is given by the choice $$i(e_1)=v_4, t(e_1)=v_1$$. The orientation of edge $$e_2$$ is given by the choice $$ i(e_2)=v_1, t(e_2)=v_2$$. The four paths shown illustrate four (out of infinitely many) itineraries $$x=(e_1, \xi )$$, $$y=(e_2, \xi ')$$ that appear in (2.9). For example, the red dotted path corresponds to $$K_f^{(--)}$$ while the green one corresponds to $$K_f^{(++)}$$

The scattering coefficient along the path $P=(e_0,..., e_m)\in \mathcal {P}_{e, e'}(m)$ is given by $S_P{:}{=}{ \Pi _{k=0}^{m-1}}S_{e_k, e_{k+1}}$. With this notation in hand let us recall the definition of a convolution operator on a metric graph with kernel $K_f(x,y), x,y\in \Gamma$ for $f:{\mathbb {R}}\rightarrow {\mathbb {R}}$, $f\in L^1({\mathbb {R}})$ satisfying

2.4 $$\begin{aligned} \sum _{m=0}^{\infty }3^m \Vert f\Vert _{L^1(m, \infty )}<\infty . \end{aligned}$$

Following [3, Definition 3.4], we define kernel $K_f$ by the formulas

2.5 $$\begin{aligned} K_f: \Gamma \times \Gamma \rightarrow {\mathbb {R}},\ K_f=K_f^{(++)}+K_f^{(+-)}+K_f^{(-+)}+K_f^{(--)}, \end{aligned}$$

where

2.6 $$\begin{aligned} K_f^{(++)}((e, \xi ), (e', \xi ')){:}{=} \delta _{e,e'}f(\xi '-\xi )+\sum _{\begin{array}{c} m\in {\mathbb {N}}\\ P\in \mathcal {P}_{e, e'}(m) \end{array}} S_Pf(\xi '+|P|-\xi ), \end{aligned}$$

with $\delta _{e,e'}=0$ if the edges *e* and $e'$ are different, and $\delta _{e, e'}=1$ otherwise,

2.7 $$\begin{aligned}&K_f^{(+-)}((e, \xi ), (e', \xi ')){:}{=} \sum _{\begin{array}{c} m\in {\mathbb {N}}\\ P\in \mathcal {P}_{e, -e'}(m) \end{array}} S_Pf(|e'|-\xi '+|P|-\xi ), \end{aligned}$$

2.8 $$\begin{aligned}&K_f^{(-+)}((e, \xi ), (e', \xi ')){:}{=} \sum _{\begin{array}{c} m\in {\mathbb {N}}\\ P\in \mathcal {P}_{-e, e'}(m) \end{array}} S_Pf(\xi '+|P|-(|e|-\xi )),\end{aligned}$$

2.9 $$\begin{aligned}&K_f^{(--)}((e, \xi ), (e', \xi ')){:}{=} \sum _{\begin{array}{c} m\in {\mathbb {N}}\\ P\in \mathcal {P}_{-e, -e'}(m) \end{array}} S_Pf(|e'|-\xi '+|P|-(|e|-\xi )). \end{aligned}$$

The above four terms represent sums along all possible finite paths on $\widetilde{\Gamma }$ connecting points $(e, \xi )\in \Gamma$ and $(e', \xi ')\in \Gamma$, see Fig. 2. Such paths are grouped into four categories depending on their itinerary which we briefly describe as follows

2.10 $$\begin{aligned}&K^{(++)}_f\text { terms correspond to paths } (e, \xi )\rightarrow (e, |e|)\rightarrow ...(e', 0)\rightarrow (e', \xi '),\nonumber \\&K^{(+-)}_f \text { terms correspond to paths } (e, \xi )\rightarrow (e, |e|)\rightarrow ...(e', |e'|)\rightarrow (e', \xi '),\nonumber \\&K^{(-+)}_f \text { terms correspond to paths } (e, \xi )\rightarrow (e, 0)\rightarrow ...(e', 0)\rightarrow (e', \xi '),\nonumber \\&K^{(--)}_f \text { terms correspond to paths } (e, \xi )\rightarrow (e, 0)\rightarrow ...(e', |e'|)\rightarrow (e', \xi '). \end{aligned}$$

Then the convolution operator $f*$ is defined by

2.11 $$\begin{aligned} (f*u)(e, \xi ){:}{=}\int _{\Gamma }K_f((e,\xi ), (e',\xi '))u(e',\xi ')d(e',\xi '), \,\,\, u\in L^p(\Gamma ), \end{aligned}$$

or simply

2.12 $$\begin{aligned} ( f*u)(\xi ){:}{=}\int _{\Gamma }K_f(\xi ,\xi ')u(\xi ')d\xi '. \end{aligned}$$

The Neumann–Kirchhoff Laplacian generates analytic semigroup $e^{t\Delta }$ in $L^p(\Gamma )$ defined by the following convolution operator

2.13 $$\begin{aligned} (e^{\Delta t}u)(\xi ){:}{=}\int _{\Gamma } K_t(\xi , \xi ')u(\xi ')d\xi ', \text { with }K_t(\xi , \xi '){:}{=}K_{f_t}(\xi , \xi '), \,\, t>0, \end{aligned}$$

where

2.14 $$\begin{aligned} f_t(x){:}{=}\frac{1}{\sqrt{4\pi t}}e^{-\frac{x^2}{4t}},\,\,\, x\in {\mathbb {R}},\,\,\, t>0, \end{aligned}$$

see [3, Theorem 4]. With these preliminary discussions we are now ready to prove Theorem 1.3.

### Proof of Theorem 1.3

(1) Let $u \in C_0^{\infty }(\Gamma )$. Using (2.12), integrating by parts, and noting that the boundary terms vanish, we obtain

2.15 $$\begin{aligned} (e^{\Delta t}\partial _x u)(x)=\int _{\Gamma } K_t(x, y)\partial _y u(y)dy = - \int _{\Gamma } (\partial _y K_t(x, y) )u(y)dy. \end{aligned}$$

Recalling (2.4), it suffices to show

2.16 $$\begin{aligned} \bigg \Vert \int _{\Gamma } \partial _y K_t^{(i,j)}(x, y) u(y)dy \bigg \Vert _{L^q(\Gamma , dx)} \le { C(T, p, q, \Gamma )}t^{-\frac{1}{2}-\frac{1}{2}\left( \frac{1}{p}-\frac{1}{q} \right) }\Vert u\Vert _{L^{p}(\Gamma )},\ i,j\in \{+,-\} \end{aligned}$$

for all $t\in (0, T]$ and some $C(T, p, q, \Gamma )>0$.

Below we will derive this inequality for $i=j=+$, the other three cases can be proved similarly. Using (2.5) we obtain

2.17 $$\begin{aligned}&\bigg \Vert \int _{\Gamma } \partial _y K_t^{(++)}(x, y) u(y)dy \bigg \Vert _{L^q(\Gamma , dx)}\nonumber \\&=\sum _{e\in \mathcal {E}} \bigg \Vert \sum _{e'\in \mathcal {E}} \int _{e'} \partial _y \big ( \delta _{e,e'}{f_t}(y-x)+\sum _{\begin{array}{c} m\in {\mathbb {N}}\\ P\in \mathcal {P}_{e, e'}(m) \end{array}}S_P{f_t}(y+|P|-x) \big ) u(y) dy \bigg \Vert _{L^q(e, dx)}\nonumber \\&\le \sum _{e\in \mathcal {E}}\bigg \Vert \int _{e} \partial _y {f_t}(y-x)u(y) dy \bigg \Vert _{L^q(e, dx)} \nonumber \\&\quad + { \sum _{e,e'\in \mathcal {E}}}\bigg \Vert \int _{e'} \sum _{\begin{array}{c} m\in {\mathbb {N}}\\ P\in \mathcal {P}_{e, e'}(m) \end{array}}S_P \partial _y {f_t}(y+|P|-x) u(y) dy \bigg \Vert _{L^q(e, dx)}\nonumber \\&=I_1+I_2, \end{aligned}$$

where $f_t$ is as in (2.13). To estimate $I_1$ let us fix an arbitrary edge $e \in \mathcal {E}$. Then one has

2.18 $$\begin{aligned} \bigg \Vert \int _{e} \partial _y {f_t}(y-x)u(y) dy \bigg \Vert _{L^q(e,dx)}&\le \bigg \Vert \int _{e} \frac{|y-x|}{2t\sqrt{4\pi t}} e^{\frac{-(y-x)^2}{4t}} |u(y)| dy \bigg \Vert _{L^q(e, dx)}\nonumber \\&\le \bigg \Vert \int _{\mathbb {R}} \frac{|y-x|}{2t\sqrt{4\pi t}} e^{\frac{-(y-x)^2}{4t}} |{ {\tilde{u}}_e(y)}| dy \bigg \Vert _{L^q(\mathbb {R}, dx)}, \end{aligned}$$

where ${ {\tilde{u}}_e(y)}$ is a function defined on $\mathbb {R}$ such that ${ {\tilde{u}}_e}(y)= u(y)$ for $y \in [0, |e|]$ and ${ {\tilde{u}}_e}(y)=0$, $y\in {\mathbb {R}}{\setminus }[0, |e|]$. Next, introducing an auxiliary function

2.19 $$\begin{aligned} h_t(z){:}{=}\frac{|z|}{2t\sqrt{4\pi t}} e^{\frac{-z^2}{4t}}, \end{aligned}$$

we notice that the right-hand side of (2.17) is equal to $\Vert h_t*|u_e|\Vert _{L^q(\mathbb {R})}$, hence, for $r\in [1,\infty )$ satisfying $q^{-1} +1 = r^{-1} + p^{-1}$ one has

2.20 $$\begin{aligned} \bigg \Vert \int _{e} \partial _y{ f_t}(y-x)u(y) dy \bigg \Vert _{L^q(e,dx)}&\le \Vert h_t*|{ {\tilde{u}}_e}|\Vert _{L^q(\mathbb {R})} \le \Vert h_z \Vert _{L^r(\mathbb {R})} \Vert { {\tilde{u}}_e}\Vert _{L^p(\mathbb {R})}\nonumber \\&=\Vert h_z \Vert _{L^r(\mathbb {R})} \Vert u\Vert _{L^p(e)}\le C t^{-\frac{1}{2}-\frac{1}{2}(\frac{1}{p}-\frac{1}{q})}\Vert u\Vert _{L^p(e)}, \end{aligned}$$

where we used

2.21 $$\begin{aligned} \Vert h_t \Vert _{L^r(\mathbb {R})} = C t^{-\frac{1}{2}-\frac{1}{2}(\frac{1}{p}-\frac{1}{q})},\ C=\frac{2^{1/r} \Vert |y| e^{-y^2} \Vert _{L^r({\mathbb {R}}, dy)}}{2\sqrt{\pi }}. \end{aligned}$$

Summing over all (finitely many) edges $e\in \mathcal {E}$ and using (2.19) we obtain

2.22 $$\begin{aligned} I_1\le C t^{-\frac{1}{2}-\frac{1}{2}(\frac{1}{p}-\frac{1}{q})}\Vert u\Vert _{L^p(\Gamma )} \end{aligned}$$

for $$C=C(p,q, \Gamma )$$, $$t>0$$.

Let us now derive a similar estimate for $I_2.$ To that end, let us fix a pair of edges $e,e'\in \mathcal {E}$ and a path *P*, $|P|>0$, connecting $e, e'$. Then for $x\in e$ one has

2.23 $$\begin{aligned}&\int _{e'} \big | S_P \partial _y {f_t}(y+|P|-x) u(y)\big | dy\le \int _{e'}\frac{|y+|P|-x|}{2t\sqrt{4\pi t}} e^{\frac{-(y+|p|-x)^2}{4t}} |u(y)| dy\nonumber \\&\quad \le \frac{1}{\sqrt{t}} \int _{e'} \bigg (\frac{|y+|p|-x|}{2\sqrt{t}} e^{\frac{-(y+|p|-x)^2}{8t}}\bigg ) \bigg (\frac{1}{\sqrt{4\pi t}}e^{\frac{-(y+|p|-x)^2}{8t}} |u(y)|\bigg ) dy\nonumber \\&\quad \le \frac{1}{\sqrt{t}}C_0 \int _{e'} \frac{1}{\sqrt{4\pi t}}e^{\frac{-(y+|p|-x)^2}{8t}} |u(y)|dy,\ t>0, \end{aligned}$$

where

2.24 $$\begin{aligned} C_0{:}{=}\sup _{z\ge 0, t>0}\bigg (\frac{z}{2\sqrt{t}} e^{\frac{-z^2}{8t}}\bigg ) \le \sqrt{2} \sup _{\zeta \in [0,\infty )} \big (\zeta e^{-\zeta ^2} \big ) <\infty . \end{aligned}$$

Let us notice that the sum appearing in $I_2$ is taken over all paths in $\mathcal {E}(\widetilde{\Gamma })$ connecting *e* and $e'$. In general, the number of such paths grows exponentially with respect to the length of the path, that is, the number of paths of length $m\in {\mathbb {N}}$ can be as high as $\deg ^m$ where $\deg$ is the largest vertex degree. The argument we give below stems from the fact that this rapid growth is balanced by an even faster decay of the heat kernel which is of order $e^{-m^2}$. To facilitate this argument we split the sum in $I_2$ into the sum over paths of large length and those whose length is order one.

For $e, e'\in \mathcal {E}$, $x\in e$, and $R>1$ using (2.22), we obtain

2.25 $$\begin{aligned}&\sum _{\begin{array}{c} m\in {\mathbb {N}}\\ P\in \mathcal {P}_{e, e'}(m) \end{array}} \int _{e'} \big | S_P \partial _y f(y+|P|-x) u(y)\big | dy \le \frac{C_0}{\sqrt{t}} \sum _{\begin{array}{c} m\in {\mathbb {N}}\\ P\in \mathcal {P}_{e, e'}(m) \end{array}} \int _{e'} \frac{1}{\sqrt{4\pi t}}e^{\frac{-(y+|P|-x)^2}{8t}} |u(y)|dy \nonumber \\&\quad \le \frac{C_0}{\sqrt{t}} \bigg (\sum _{\begin{array}{c} m \le R\\ P\in \mathcal {P}_{e, e'}(m) \end{array}} \int _{e'} \frac{1}{\sqrt{4\pi t}}e^{\frac{-(y+|P|-x)^2}{8t}} |u(y)|dy + \sum _{\begin{array}{c} m > R\\ P\in \mathcal {P}_{e, e'}(m) \end{array}} \int _{e'} \frac{1}{\sqrt{4\pi t}}e^{\frac{-(y+|P|-x)^2}{8t}} |u(y)|dy \bigg )\nonumber \\&\quad = \frac{C_0}{\sqrt{t}} \bigg (A+B\bigg ). \end{aligned}$$

The choice^6^ of *R* that yields the splitting of $I_2$ into the sum over paths of large length and those whose length is order one is given by

2.26 $$\begin{aligned} R{:}{=}(2\ell _+^2 + 8T\log {(\deg )}+1)\ell _-^{-2}, \end{aligned}$$

where $\ell _{\pm }>0$ are lower and upper bounds on length of edges. This choice stems from the fact for $m>R$ one has

2.27 $$\begin{aligned} \deg ^m e^{\frac{2m\ell _+^2-(ml_-)^2}{8t}}\le e^{-\frac{m}{8t}}, t\in (0,T], \end{aligned}$$

which is a crucial estimate for the following chain of inequalities. Noting that for a path $P\in \mathcal {P}_{e,e'}(m)$ one has $|P| \ge m\ell _-$ and $(y-x)|P| \ge -m\ell _+^2$ we obtain

2.28 $$\begin{aligned} B&=\sum _{\begin{array}{c} m> R \\ P\in \mathcal {P}_{e, e'}(m) \end{array}} \int _{e'} \frac{1}{\sqrt{4\pi t}}e^{\frac{-(y+|P|-x)^2}{8t}} |u(y)|dy \nonumber \\&\le \sum _{m> R} \deg ^m \int _{e'} \frac{1}{\sqrt{4\pi t}}e^{\frac{-(y-x)^2 -2(y-x)|P|-|P|^2}{8t}} |u(y)|dy \nonumber \\&\le \sum _{m> R} \deg ^m \int _{e'} \frac{1}{\sqrt{4\pi t}}e^{\frac{-(y-x)^2 +2m\ell _+^2-(ml_-)^2}{8t}} |u(y)|dy \nonumber \\&\le \sum _{m\in {\mathbb {N}},m > R } e^{\frac{m(2\ell _+^2+8t\log (\deg ) - R\ell _-^2)}{8t}} \int _{e'} \frac{1}{\sqrt{4\pi t}}e^{\frac{-(y-x)^2}{8t}} |u(y)|dy \nonumber \\&\le \sum _{m\in {\mathbb {N}}} e^{-\frac{m}{8t}} \int _{e'} \frac{1}{\sqrt{4\pi t}}e^{\frac{-(y-x)^2}{8t}} |u(y)|dy\nonumber \\&\le \frac{1}{1-e^{-\frac{1}{8t}}} \int _{\mathbb {R}} \frac{1}{\sqrt{4\pi t}}e^{\frac{-(y-x)^2}{8t}} |{ {\tilde{u}}_{e'}(y)}|dy, t\in (0,T] \end{aligned}$$

where, as before, we let ${ {\tilde{u}}_{e'}}$ denote the extension of *u* by zero outside of $(0, |e'|)$. Next, introducing an auxiliary function

2.29 $$\begin{aligned} \varphi _t(z){:}{=}\frac{1}{\sqrt{4\pi t}}e^{-z^2/8t}, \text { with } \Vert \varphi _t\Vert _{L^r({\mathbb {R}})}\le C t^{{-\frac{1}{2}}+\frac{1}{2r}}, r\ge 1, C=C(r)>0, \end{aligned}$$

we use (2.27) to obtain

2.30 $$\begin{aligned} \Vert B\Vert _{L^q(e)}&\le \frac{\Vert \varphi _t*(|u_{e'}|)\Vert _{L^q(e)}}{1-e^{-\frac{1}{8t}}}\le \frac{\Vert \varphi _t\Vert _{L^r({\mathbb {R}})} \Vert { {\tilde{u}}_{e'}}\Vert _{L^p(e')}}{1-e^{-\frac{1}{8t}}}\nonumber \\&\le C\frac{t^{{-\frac{1}{2}}+\frac{1}{2r}}}{1-e^{-\frac{1}{8t}}}{ \Vert {\tilde{u}}_{e'}\Vert _{L^p(e')}}, \end{aligned}$$

where $$1+q^{-1}=r^{-1}+p^{-1}$$.

In order to estimate term *A* in (2.24) we change variables $z= x-y-|P|$ and obtain

2.31 $$\begin{aligned}&\sum _{\begin{array}{c} m\in {\mathbb {N}}, m \le R\\ P\in \mathcal {P}_{e, e'}(m) \end{array}} \int _{e'} \frac{1}{\sqrt{4\pi t}}e^{\frac{-(y+|P|-x)^2}{8t}} |u(y)|dy =\sum _{\begin{array}{c} m\in {\mathbb {N}}, m \le R\\ P\in \mathcal {P}_{e, e'}(m) \end{array}} \int _{{\mathbb {R}}} \frac{1}{\sqrt{4\pi t}}e^{\frac{-(y+|P|-x)^2}{8t}} |{ {\tilde{u}}_{e'}(y)}|dy\nonumber \\&\quad \le \sum _{\begin{array}{c} m\in {\mathbb {N}}, m \le R\\ P\in \mathcal {P}_{e, e'}(m) \end{array}}\int _{\mathbb {R}} \frac{1}{\sqrt{4\pi t}}e^{\frac{-z^2}{8t}} |{ {\tilde{u}}_{e'}}(x-z-|P|)|dz, \end{aligned}$$

for arbitrary $x\in e$. Then one has

2.32 $$\begin{aligned} \Vert A\Vert _{L^q(e)}\le \sum _{\begin{array}{c} m\in {\mathbb {N}}, m \le R\\ P\in \mathcal {P}_{e, e'}(m) \end{array}} C\Vert \varphi _t\Vert _{L^r(\mathbb {R})}\Vert { {\tilde{u}}_{e'}}(\cdot -|P|) \Vert _{L^p(\mathbb {R})} \le C( T, p,q, \Gamma )t^{-\frac{1}{2}(\frac{1}{p}-\frac{1}{q})} { \Vert u\Vert _{L^p(e')}.} \end{aligned}$$

Finally, combining (2.21), (2.24), (2.29), (2.31) we obtain

2.33 $$\begin{aligned} \Vert e^{(\Delta -\sigma )t}\partial _x u\Vert _{L^q(\Gamma )}\le C t^{-1/2}\left( t^{-\frac{1}{2}(\frac{1}{p}-\frac{1}{q})}+\frac{t^{-\frac{1}{2}(\frac{1}{p}-\frac{1}{q})}}{1-e^{-\frac{1}{8t}}}\right) \Vert u\Vert _{L^p(\Gamma )}, t\in (0, T], \end{aligned}$$

where $C=C(T, p, q, \Gamma )$ as asserted in (1.21) and (1.23). The proof of (1.22) and (1.24) is analogous.

(2) the derivation of (1.25) is analogous to that of (1.21). The inequality (1.26) follows from (1.24) and (1.25). $\square$

### Corollary 2.1

Assume the setting of Theorem 1.3. Then there exists $\sigma _0$ such that the following assertions hold. For every $\sigma >\sigma _0$ there exists $\delta \in (0,\sigma )$^7^ and $C=C(\sigma , \delta , p, q, \Gamma )>0$ such that for all $t>0$ one has

2.34 $$\begin{aligned}&||e^{(\Delta -\sigma )t}||_{\mathcal {B}( L^p(\Gamma ), L^q(\Gamma ))} \le Ce^{-\delta t} t^{-\frac{1}{2}(\frac{1}{p} -\frac{1}{q})}, \end{aligned}$$

2.35 $$\begin{aligned}&\Vert e^{(\Delta -\sigma ) t}\partial _x \Vert _{\mathcal {B}( L^p(\Gamma ), L^q(\Gamma ))}\le Ce^{-t\delta }t^{-\frac{1}{2}-\frac{1}{2}\left( \frac{1}{p}-\frac{1}{q} \right) }, \end{aligned}$$

2.36 $$\begin{aligned}&\Vert \partial _xe^{(\Delta -\sigma ) t} u\Vert _{\mathcal {B}( L^p(\Gamma ), L^q(\Gamma ))}\le Ce^{-t\delta }t^{-\frac{1}{2}-\frac{1}{2}\left( \frac{1}{p}-\frac{1}{q} \right) }. \end{aligned}$$

### Proof

Let us prove (2.33), the other two inequalities can be proved analogously. Let $C(T)>0$ abbreviate the constant $C(T, p,q, \Gamma )$ from Theorem 1.3. For arbitrary $t>0$ let us write $t=\tau +n$ for $\tau \in (0, 1]$ and $n\in {\mathbb {N}}_0$.

If $n=0$ then by (1.25) one has

2.37 $$\begin{aligned} ||e^{(\Delta -\sigma )t}||_{\mathcal {B}( L^p(\Gamma ), L^q(\Gamma ))}\le C(1) e^{-\sigma t} t^{-\frac{1}{2}(\frac{1}{p} -\frac{1}{q})}. \end{aligned}$$

Assume now that $n\ge 1$ then one has

2.38 $$\begin{aligned}&||e^{(\Delta -\sigma )t}||_{\mathcal {B}( L^p(\Gamma ), L^q(\Gamma ))} = ||e^{(\Delta -\sigma )(\tau +n)}||_{\mathcal {B}( L^p(\Gamma ), L^q(\Gamma ))}\end{aligned}$$

2.39 $$\begin{aligned}&\le (C(1))^{n-1} e^{-\sigma t}\Vert e^{ \Delta (\tau +1) }||_{\mathcal {B}( L^p(\Gamma ), L^q(\Gamma ))}\end{aligned}$$

2.40 $$\begin{aligned}&\le (C(1))^{n-1} C(2)e^{-\sigma t}(1+\tau )^{-\frac{1}{2}\left( \frac{1}{p}-\frac{1}{q}\right) }. \end{aligned}$$

Without loss of generality we may assume that $\alpha {:}{=}\log (\max (C(1), C(2)))>0$. Then for $\sigma > \alpha$ the above chain of inequalities yields

2.41 $$\begin{aligned} ||e^{(\Delta -\sigma )t}||_{\mathcal {B}( L^p(\Gamma ), L^q(\Gamma ))}&= ||e^{(\Delta -\sigma )(\tau +n)}||_{\mathcal {B}( L^p(\Gamma ), L^q(\Gamma ))}\le e^{n\alpha -\sigma n-\sigma \tau }(1+\tau )^{-\frac{1}{2}\left( \frac{1}{p}-\frac{1}{q}\right) }\end{aligned}$$

3.1 $$\begin{aligned}&=Ce^{-(\sigma -\alpha )t}\le C e^{-(\sigma -\alpha )t/2}t^{{-\frac{1}{2}\left( \frac{1}{p}-\frac{1}{q}\right) }}, \end{aligned}$$

where $$C=C(\alpha ,\sigma , p,q)>0$$. $$\square $$

## Linear non-autonomous parabolic equations and proof of Theorem 1.4

In this section, we study the linear non-autonomous parabolic equation (1.27) and prove Theorem 1.4.

### Proof of Theorem 1.4

Let us define an operator

3.2 $$\begin{aligned} V(t)\psi :=a({ t,\cdot }) \psi _x+b({ t,\cdot })\psi , \quad \psi \in \mathcal {X}^{\alpha }_q. \end{aligned}$$

We notice that if $\alpha >0$ is such that $\mathcal {X}^{\alpha }_q\hookrightarrow \widehat{C}^1(\Gamma )$ then one has

3.3 $$\begin{aligned} \Vert V(t)\psi \Vert _{L^q(\Gamma )}\le C \Vert \psi \Vert _{\mathcal {X}^{\alpha }_q}\left( \Vert a(t,\cdot )\Vert _{L^q(\Gamma )}+\Vert b(t,\cdot )\Vert _{L^q(\Gamma )}\right) , \end{aligned}$$

and

3.4 $$\begin{aligned} \mathcal {V}\in C^{\beta }\left( (0, T), \mathcal {B}(\mathcal {X}^{\alpha }_q, L^q(\Gamma )) \right) , \end{aligned}$$

where we used Hölder continuity of *a*, *b* in time.

Next, consider integral equation (1.28). Our first objective is to prove existence of solutions with initial data drawn from a fractional power space $\mathcal {X}^{\alpha }_q$. To that end, fix arbitrary $\psi _0\in \mathcal {X}^{\alpha }_q$. Then the Hölder continuity of $t\mapsto V(t)$ on $[\kappa ,\tau ]\subset (0,T)$ and Theorem C.2^8^ yield the existence of a unique $\Psi = \Psi (t; \psi _0,\kappa )$ such that

3.5 $$\begin{aligned}&\Psi \in C([\kappa , \tau ], L^q(\Gamma )), \Psi (t; \psi _0,\kappa )\in \mathcal {X}^{\alpha }_q,\\&\Psi (t;\psi _0,\kappa )=e^{(\Delta -\sigma )(t-\kappa )}\psi _0+\int _{\kappa }^{t}e^{(\Delta -\sigma )(t-\kappa -s)}V(s)\Psi (s;\psi _0,\kappa )ds, t\in [\kappa , \tau ], \end{aligned}$$

where the integral converges in $L^q(\Gamma )$. By [26, Theorem 7.1.1 (b)], for arbitrary $\gamma \in (0,1)$ we have $\Psi (t;\psi _0,\kappa )\in \mathcal {X}^{\gamma }_q$ whenever $\psi _0\in \operatorname {dom}(\Delta )$. This gives rise to the evolution operator $T(t,\kappa )$ defined by

3.6 $$\begin{aligned} T(t,\kappa ): \operatorname {dom}(T(t,\kappa )){:}{=}\operatorname {dom}(\Delta )\subset L^q(\Gamma )\rightarrow \mathcal {X}^{\gamma }_q,\quad T(t,\kappa )\psi _0=\Psi (t;\psi _0,\kappa ). \end{aligned}$$

We then show that $T(t,\tau )$ can be extended to a bounded linear operator in $\mathcal {B}(L^q(\Gamma ), \mathcal {X}^{\gamma }_q)$ satisfying

$$\begin{aligned} \Vert T(t,\kappa )\Vert _{\mathcal {B}(L^q(\Gamma ), \mathcal {X}^{\gamma }_q)}\le C(t-\kappa )^{-\gamma }. \end{aligned}$$

To that end, we may assume without loss of generality that $1<2\gamma -\frac{1}{q}$ so that $\mathcal {X}^{\gamma }_q\hookrightarrow \widehat{C}^1(\Gamma )$. We first show that there exists a constant $C>0$ such that

3.7 $$\begin{aligned} \left\| \Psi (t; f,\kappa )-\Psi (t; g,\kappa ) \right\| _{\mathcal {X}^{\gamma }_q}\le C (t-\kappa )^{-\gamma }\Vert f-g\Vert _{L^q(\Gamma )}, \end{aligned}$$

for all $t\in (\kappa ,\tau ]$, $f,g\in \operatorname {dom}(\mathcal {A}_q)$. Since $\Psi (\cdot ; f,\kappa )$, $\Psi (\cdot ; g,\kappa )$ satisfy (3.5), one has

3.8 $$\begin{aligned}&\Vert \Psi (t;f,\kappa )-\Psi (t;g,\kappa )\Vert _{\mathcal {X}^{\gamma }_q}\le C\Vert (\sigma -\Delta )^{\gamma }e^{(\Delta -\sigma )(t-\kappa )}(f-g)\Vert _{L^q(\Gamma )}\nonumber \\&\quad +C\int _{\kappa }^t\Vert (\sigma -\Delta )^{\gamma }e^{(\Delta -\sigma )(t-\kappa -s)}V(s)(\Psi (s;f,\kappa )-\Psi (s;g,\kappa ))\Vert _{L^q(\Gamma )}ds, \end{aligned}$$

and, using (A.10),

3.9 $$\begin{aligned}&\Vert \Psi (t;f,\kappa )-\Psi (t;g,\kappa )\Vert _{\mathcal {X}^{\gamma }_q}\le C(t-\kappa )^{-\gamma }\Vert (f-g)\Vert _{L^q(\Gamma )}\nonumber \\&\quad +C \int _{\kappa }^t(t-\kappa -s)^{-\gamma }\Vert V(s)(\Psi (s;f,\kappa )-\Psi (s;g,\kappa ))\Vert _{L^q(\Gamma )}ds. \end{aligned}$$

We claim

3.10 $$\begin{aligned} \Vert V(s)(\Psi (s;f,\kappa )-\Psi (s;g,\kappa ))\Vert _{L^q(\Gamma )}\le C\Vert (\Psi (s;f,\kappa )-\Psi (s;g,\kappa ))\Vert _{\mathcal {X}^{\gamma }_q}. \end{aligned}$$

Indeed, one has

3.11 $$\begin{aligned}&\Vert V(s)(\Psi (s;f,\kappa )-\Psi (s;g,\kappa ))\Vert _{L^q(\Gamma )}\nonumber \\&\quad \le \Vert a(s,\cdot )(\Psi _x(s;f,\kappa )-\Psi _x(s;g,\kappa ))\Vert _{L^q(\Gamma )} \nonumber \\&\qquad + \Vert b(s,\cdot )(\Psi (s;f,\kappa )-\Psi (s;g,\kappa ))\Vert _{L^q(\Gamma )}\nonumber \\&\quad \le C{ (\Vert a(s,\cdot )\Vert _{L^q(\Gamma )}+\Vert b(s,\cdot )\Vert _{L^q(\Gamma )})}\Vert (\Psi (s;f,\kappa )-\Psi (s;g,\kappa ))\Vert _{\mathcal {X}^{\gamma }_q}, \end{aligned}$$

where in the last step we used the embedding $\mathcal {X}^{\gamma }_q\hookrightarrow \widehat{C}^1(\Gamma )$. Since $s\mapsto a(s,\cdot )$, $s\mapsto b(s,\cdot )$ are Hölder continuous on $[\kappa ,\tau ]$, the required inequality (3.10) follows from (3.11). Finally, combining (3.9), (3.10) and Gronwall’s inequality (C.36), (C.37) we obtain (3.7).

Next, we construct a bounded extension of $T(t,\tau )$ introduced in (3.6). Let $\psi _n\in \operatorname {dom}(\mathcal {A}_q)$ be such that $\Vert \psi _n-\psi _0\Vert _{L^q(\Gamma )}\rightarrow 0$ as $n\rightarrow \infty$. Then as we just proved one has $\Psi (t;\psi _n,\kappa )\in \mathcal {X}^{\gamma }_q$ and

3.12 $$\begin{aligned} \Psi (t;\psi _n,\kappa )=e^{(\Delta -\sigma )(t-\kappa )}\psi _n+\int _{\kappa }^{t}e^{(\Delta -\sigma )(t-\kappa -s)}V(s)\Psi (s; \psi _n,\kappa )ds, t\in [\kappa , \tau ]. \end{aligned}$$

Using (3.7) with $f=\psi _n$, $g=\psi _m$ we infer that the sequence $\{\Psi (t; \psi _n,\kappa )\}_{n=1}^{\infty }$ converges in $\mathcal {X}^{\gamma }_q$ to $\Psi _\infty (t)$ for $t\in (\tau , \kappa ]$. To pass to the limit in (3.12) we observe that

3.13 $$\begin{aligned}&\Vert V(s)\Psi (s;\psi _n,\kappa )\Vert _{L^q(\Gamma )}\le \Vert V(s)\Vert _{\mathcal {B}(\mathcal {X}^{\gamma }_q, L^q(\Gamma ))}\Vert \Psi (s; \psi _n,\kappa )\Vert _{\mathcal {X}^{\gamma }_q}\nonumber \\&\quad \le C (s-\kappa )^{{-\gamma }}\Vert V(s)\Vert _{\mathcal {B}(\mathcal {X}^{\gamma }_q, L^q(\Gamma ))}\Vert \psi _n\Vert _{L^q(\Gamma )}\le C (s-\kappa )^{{-\gamma }}, s\in (\kappa , \tau ], \end{aligned}$$

where we used continuity of $s\mapsto V(s)\in \mathcal {B}(\mathcal {X}^{\gamma }_q, L^q(\Gamma ))$, boundedness of $\{\Vert \psi _n\Vert _{L^q(\Gamma )}\}_{n=1}^{\infty }$, and $\gamma \in (0,1)$. Finally, recalling that $\psi _n\rightarrow \psi _0$ in $L^q(\Gamma )$, we pass to the limit as $n\rightarrow \infty$ in (3.12) which yields

3.14 $$\begin{aligned} \Psi _\infty (t)=e^{(\Delta -I)(t-\kappa )}\psi _0+\int _{\kappa }^{t}e^{(\Delta -I)(t-\kappa -s)}V(s)\Psi _\infty (s)ds,\ t\in [\kappa , \tau ]. \end{aligned}$$

We claim that $\Psi _\infty (t)$ does not depend on the choice of the approximating sequence $\{\psi _n\}$ but only on $\psi _0$. To that end, let $\widetilde{\psi }_n\in \operatorname {dom}(\mathcal {A}_q)$ be such that $\Vert \widetilde{\psi }_n-\psi _0\Vert _{L^q(\Gamma )}\rightarrow 0, n\rightarrow \infty$. Proceeding as above, we note that $\{\Psi (t;\widetilde{\psi }_n,\kappa )\}_{n=1}^{\infty }$ converges in $\mathcal {X}^{\gamma }_q$ to $\widetilde{\Psi }_\infty (t)$ for $t\in (\kappa ,\tau ]$ and

3.15 $$\begin{aligned} {\tilde{\Psi }}_\infty (t)=e^{(\Delta -\sigma )(t-\kappa )}\psi _0+\int _{\kappa }^{t}e^{(\Delta -\sigma )(t-\kappa -s)}V(s){\tilde{\Psi }}_\infty (s)ds,\ t\in [\kappa , \tau ]. \end{aligned}$$

By (3.14) and (3.15),

3.16 $$\begin{aligned} \Psi _\infty (t)-\widetilde{\Psi }_\infty (t)=\int _{\kappa }^t e^{(\Delta -\sigma )(t-\kappa -s)}V(s)\big (\Psi _\infty (s)-\widetilde{\Psi }_\infty (s)\big )ds,\ t\in (\kappa ,\tau ], \end{aligned}$$

hence, for $t\in (\kappa , \tau ]$ one has

3.17 $$\begin{aligned}&\Vert \Psi _\infty (t)-\widetilde{\Psi }_\infty (t)\Vert _{L^q(\Gamma )} \le C\int _\kappa ^t \Vert \Psi _\infty (s)-\widetilde{\Psi }_\infty (s)\Vert _{\mathcal {X}_q^\gamma }ds\nonumber \\&\quad =C\int _\kappa ^t \lim _{n\rightarrow \infty } \Vert \Psi (s;\psi _n)-\Psi (s;{\tilde{\psi }}_n)\Vert _{\mathcal {X}_q^\gamma } ds\nonumber \\&\quad \le C\int _{\kappa }^t (s-\kappa )^{-\gamma }\lim _{n\rightarrow \infty }\Vert \psi _n-\widetilde{\psi }_n\Vert _{L^q}ds=0. \end{aligned}$$

It then follows that $\Psi _\infty (t)= {\tilde{\Psi }}_\infty (t), t\in [\kappa ,\tau ].$ Then we define $T(t,\kappa )\psi _0{:}{=}\lim _{n\rightarrow \infty }T(t,\kappa )\psi _n,$ and note that $T(t,\kappa )$ is a single-valued linear operator defined on $L^q(\Gamma )$. Moreover, denoting $\Psi (t;\psi _0,\kappa ){:}{=}T(t,\kappa )\psi _0$ one has

$$\begin{aligned} \Psi (t;\psi _0,\kappa )=e^{(\Delta -\sigma )(t-\kappa )}\psi _0+\int _\kappa ^t e^{(\Delta -\sigma )(t-\kappa -s)} V(s) \Psi (s;\psi _0,\kappa )ds,\ t\in [\kappa ,\tau ], \end{aligned}$$

and by (3.7) with $f=\psi _0$, $g=0$

$$\begin{aligned} \left\| T(t,\kappa )\psi _0\right\| _{\mathcal {X}^{\gamma }_q}=\left\| \Psi (t; \psi _0,\kappa ) \right\| _{\mathcal {X}^{\gamma }_q}\le C (t-\kappa )^{-\gamma }\Vert \psi _0\Vert _{L^q(\Gamma )}. \end{aligned}$$

This gives a unique solution of (1.28) with initial condition in $L^q(\Gamma )$ as well as the asserted bound for the norm of the evolution operator.

Let us now prove the last part of the theorem. Since $$s\mapsto V(s)\in \mathcal {B}(\mathcal {X}^{\alpha }_q, L^q(\Gamma ))$$ is Hölder continuous on $$[\kappa , \tau ]$$, Theorem C.1 with $$f(t,x, u,v){:}{=}a(t,x)v+b(t,x)u, $$ yields a strong solution, cf. Definition C.1, $${ \Psi (t,x; \psi _0,\kappa ){:}{=}\Psi (t;\psi _0,\kappa )(x)}$$ of (1.27) in $$L^q(\Gamma )$$. We claim that this solution is in fact classical. To prove this claim, let us fix an arbitrary $$\varepsilon \in (0, { T-\kappa })$$ and note that $${ \Psi _0{:}{=}}\Psi (t,\cdot ; \kappa +\varepsilon , \psi _0)\in \mathcal {X}^{\gamma }_q$$ for all $$\gamma \in (0,1)$$. Finally, by picking $$\gamma >0$$ so that $$2\gamma -p^{-1}>1$$ and using Theorem C.2 with initial time $$\kappa +\varepsilon $$ we conclude that $$ { \Psi (\cdot +\varepsilon ,\cdot ;\psi _0,\kappa )=}\Psi (\cdot , \cdot ;\Psi _0,\kappa +\varepsilon )\in \widehat{C}^{1,2}({ (\kappa +\varepsilon ,T)} \times {\overline{\Gamma }}), $$ as required. $$\square $$

## Local existence of classical solutions for general chemotaxis model

The main purpose of this section is to provide the proof of Theorem 1.1.

### Outline of the proof of Theorem 1.1

Noting that it suffices to consider two cases, $\tau =0$ and $\tau =1$ (the proof for $\tau >0$ is similar), we outline the strategy of the proof next. To begin, we observe that the Duhamel’s principle applied to the reaction-advection–diffusion system (1.5) with $\tau =1$ gives rise to a system of integral equations

4.1 $$\begin{aligned} {\left\{ \begin{array}{ll} u(t)& =e^{(\Delta -\sigma )t}u_0-\int _{0}^{t} e^{(\Delta -\sigma )(t-s)}\partial _{x}\big (f_1(u(s), v(s))\partial _xv(s)\big )ds\\ & \quad + \int _{0}^{t} e^{(\Delta -\sigma )(t-s)}(f_2(u(s), v(s))+\sigma u(s))ds,\\ v(t)& =e^{(\Delta -\sigma I)t}v_0+ \int _{0}^{t} e^{(\Delta -\sigma )(t-s)}\left( f_3(u(s), v(s))+\sigma v(s)\right) ds, \\ \end{array}\right. } \end{aligned}$$

and, similarly, in case $\tau =0,$ to the following integral equation

4.2 $$\begin{aligned} {\left\{ \begin{array}{ll} u(t)& =e^{(\Delta -\sigma )t}u_0+\int _{0}^{t} e^{(\Delta -\sigma )(t-s)}\partial _x(f_1(u(s), v(s))\partial _x v(s))ds\\ & \quad +\int _{0}^{t} e^{(\Delta -\sigma )(t-s)}\left( f_1(u(s), v(s))+\sigma v(s)\right) ds,\\ & v(s){:}{=}-(\Delta -\sigma )^{-1}{f(u(s))}, \end{array}\right. } \end{aligned}$$

where we used $f_3(u,v)=f(u)-\sigma v$ in (4.2) (see Hypothesis 1.1).

Our first objective, see Sect. 4.2, is to show that the integrals in (4.1), (4.2) are convergent in suitable Sobolev and fractional power spaces $\mathcal {X}^{\beta }_p$ whenever $u\in C([0, {T}], L^p(\Gamma ))$, $v\in C([0, {T}], \widehat{W}^{1, p}(\Gamma ))$, see Propositions 4.1, 4.2. In Sect. 4.2, we also show that these integrals, as functions of *t*, are Hölder continuous, see Proposition 4.3. Our proofs rely on the $L^p-L^q$ mapping properties of nonlinear operators discussed in Appendix B.

Our second objective is to construct solutions of (4.1) in

4.3 $$\begin{aligned} C([0, T], L^p(\Gamma )\times L^p(\Gamma ))\cap C((0, T_{\max }), L^p(\Gamma )\times \widehat{W}^{1, p}(\Gamma )), \end{aligned}$$

with initial data $(u_0, v_0)\in L^p(\Gamma )\times \widehat{W}^{1, p}(\Gamma )$ and solutions of (4.2) in

4.4 $$\begin{aligned} C([0, T_{\max }), L^p(\Gamma ))\text { with initial data } u_0\in L^p(\Gamma ), \end{aligned}$$

for a maximal $T_{\max }\in (0,\infty ]$ via a fixed point argument and an extension argument. These solutions will be referred to as *mild solutions of* (1.5) *subject to* (1.6),(1.7). To that end, for $T>0$ and $(u_0, v_0)\in L^p(\Gamma )\times \widehat{W}^{1, p}(\Gamma )$, we formally define a pair of mappings

4.5 $$\begin{aligned} \Lambda (u_0,v_0): (u,v)\mapsto \Lambda (u,v;u_0,v_0)=(\Phi (u,v;u_0)), \Psi (u,v;v_0)) \end{aligned}$$

for $$ (u,v)\in C([0, {T]}, L^p(\Gamma )\times \widehat{W}^{1, p}(\Gamma ))$$, and

4.6 $$\begin{aligned} \Theta (u_0): u\mapsto \Theta (u;u_0)\quad \textrm{for}\quad u\in C([0, {T]}, L^p(\Gamma )) \end{aligned}$$

by the following formulas

4.7 $$\begin{aligned} (\Phi (u,v;u_0)(t)&=e^{(\Delta -\sigma )t}u_0-\int _{0}^{t} e^{(\Delta -\sigma )(t-s)}\partial _{x}\big (f_1(u(s), v(s))\partial _xv(s)\big )ds\nonumber \\&\quad + \int _{0}^{t} e^{(\Delta -\sigma )(t-s)}(f_2(u(s), v(s))+\sigma u(s))ds, \end{aligned}$$

4.8 $$\begin{aligned} (\Psi (u,v;v_0))(t)&=e^{(\Delta -\sigma I)t}v_0+ \int _{0}^{t} e^{(\Delta -\sigma )(t-s)}\left( f_3(u(s), v(s))+\sigma v(s)\right) ds, \end{aligned}$$

4.9 $$\begin{aligned} (\Theta (u;u_0))(t)&{:}{=} (\Phi (u, -(\Delta -\sigma )^{-1}f(u))(t), t\in {[0,T]}. \end{aligned}$$

In Sect. 4.3, we prove that $\Lambda , \Theta$ are contractions for small *T* provided that $v_0\in \mathcal {X}_p^{\frac{1}{2}+\epsilon }$, for some $\epsilon \in (0,1)$ (see Proposition  4.4). Then we use the fixed points of these mappings together with some approximation arguments, to obtain a unique solution $(u(t),v(t))=(u(t;u_0,v_0),v(t;u_0,v_0))$ to (4.1) (resp. a unique solution $(u(t),v(t))=(u(t;u_0),v(t;u_0))$ to (4.2)) on a small interval [0, *T*] for initial data $(u_0, v_0)\in L^{p}(\Gamma )\times \widehat{W}^{1, p}(\Gamma )$, see Proposition  4.5. By an extension argument we obtain a unique solution $(u(t),v(t))=(u(t;u_0,v_0),v(t;u_0,v_0))$ to (4.1) (resp. a unique solution $(u(t),v(t))=(u(t;u_0),v(t;u_0))$ to (4.2)) on the maximal interval $[0,T_{\max })=[0,T_{\max }(u_0,v_0))$ (resp. on the maximal interval $[0,T_{\max })=[0,T_{\max }(u_0))$ with the following regularity properties (see Theorem 4.1),

$$\begin{aligned} u\in {C^{{ \beta }}}((0, T_{\max }), \widehat{C}^{\nu }(\overline{\Gamma }))\cap { C^{\beta }}((0, T_{\max }), \mathcal {X}^{\beta }_r)\text { for arbitrary } \, r\ge 1,\,\,\, 0<\nu<\beta <\frac{1}{8}, \end{aligned}$$

and

$$\begin{aligned}&v\in {C^{1+\beta }} ((0,T_{\max }), \mathcal {X}^{\beta }_r)\cap {C^{\beta }}((0, T_{\max }), \widehat{C}^{2+\nu }(\overline{\Gamma }))\quad \text {when}\,\, (u,v)\,\, \text { is the solution of } (4.1),\\&v\in {C^{\beta }}((0, T_{\max }), \widehat{C}^{2+\nu }(\overline{\Gamma }))\quad \text {when},\, (u,v)\,\, \text { is the solution of } (4.2). \end{aligned}$$

This range of values of parameter $\beta$ does not yield the embedding $\mathcal {X}^{\beta }_p\hookrightarrow \widehat{C}^1(\Gamma )$, which is an obstacle to showing that the obtained mild solutions are actually classical solutions of (1.5) subject to (1.6),(1.7).

Our third, and final, objective is to show higher time and space regularity of the mild solution and then prove Theorem 1.1. To that end, we formally rewrite (1.5) as follows

4.10 $$\begin{aligned}&u_t=(\Delta -\sigma )u-a(t,x)\partial _xu+b(t,x)u,\ \ \ \end{aligned}$$

4.11 $$\begin{aligned}&\tau v_t=\partial _{xx}^2 v+f_3(u,v), \end{aligned}$$

where

4.12 $$\begin{aligned} a(t,\cdot )&=\partial _uf_1(u(t),v(t))\partial _x v(t),\nonumber \\ b(t,\cdot )&=\left( \frac{f_2(u,v)+\sigma u- f_1(u,v)\partial _{xx}v-\partial _vf_1(u,v)(\partial _{x}v)^2}{u}\right) . \end{aligned}$$

Applying Theorem 1.4, we first show the existence and uniqueness of classical solutions to the above linear system. We then show that the obtained classical solution to the linear system coincides with the mild solution of (1.5) subject to (1.6),(1.7) from the previous step. To carry out this item we use explicit estimate on the norm of the evolution operator from Theorem 1.4 and a version of Gronwall’s inequality recalled in Proposition C.1. This line of arguments leads to $(u(t),v(t))\in \mathcal {X}_r^\alpha$ for arbitrary $t\in (0,T_{\max })$, $r\ge 1$ and $\alpha \in (0, 1)$. This regularity is enough for us to show that the obtained mild solutions are actually classical solutions of (1.5) subject to (1.6), (1.7).

### Rigorous formulation of integral equations

In Propositions 4.1, 4.2, 4.3, we prove that the integral in (4.7) converges absolutely in $L^p(\Gamma )$, the integral in (4.8) converges in $\widehat{W}^{1, p}(\Gamma )$ both to elements of $\mathcal {X}^{\beta }_p(\Gamma )$. We also examine regularity properties of the mappings $t\mapsto \Phi (t), \Psi (t), \Theta (t)$ in $L^p(\Gamma )$ and in $\mathcal {X}_p^{\beta }$ for a suitable range of parameters $\beta , p$.

#### Hypothesis 4.1

Assume Hypothesis 1.1. Let us fix $$ T>0$$, $$u\in C([0, {T}], L^p(\Gamma ))$$, $$v\in C([0, {T}], \widehat{W}^{1, p}(\Gamma ))$$, $$t\in (0,T)$$, $$s\in (0,t). $$

#### Proposition 4.1

Assume Hypothesis 4.1. Then one has

4.13 $$\begin{aligned} f_1(u(s), v(s))\partial _xv(s)\in L^{\frac{p}{2\gamma }}(\Gamma ). \end{aligned}$$

The operator $e^{(\Delta -\sigma )(t-s)}\partial _x$ originally defined on $\widehat{C}_0^{\infty }(\Gamma )$ can be extended to a bounded linear operator in $\mathcal {B}(L^{\frac{p}{2\gamma }}(\Gamma ), L^p(\Gamma ))$. For such an extension one has

4.14 $$\begin{aligned} F_1(s):= e^{(\Delta -\sigma )(t-s)}\partial _x\big (f_1(u(s), v(s))\partial _xv(s) \big )\in L^p(\Gamma ), \end{aligned}$$

and, in addition, we have

4.15 $$\begin{aligned}&F_2(s):=e^{(\Delta -{\sigma }I)(t-s)}(f_2(u(s),v(s))+\sigma u(s))\in \widehat{W}^{1, p}(\Gamma ), \end{aligned}$$

4.16 $$\begin{aligned}&F_3(s):=e^{(\Delta -{ \sigma }I)(t-s)}{ \big (f_3(u(s),v(s))+\sigma u(s)\big )}\in \widehat{W}^{1, p}(\Gamma ), \end{aligned}$$

and the functions $(0,t)\ni s\mapsto F_1(s)\in L^p(\Gamma )$, $(0,t)\ni s\mapsto F_2(s)\in \widehat{W}^{1, p}(\Gamma )$, $(0,t)\ni s\mapsto F_3(s)\in \widehat{W}^{1, p}(\Gamma )$ are integrable on (0, *t*).

#### Proof

Inclusions (4.13), (4.14) follow from Proposition B.5 with $F=f_1$ and

4.17 $$\begin{aligned} R{:}{=}\max _{s\in [0,T]} \Vert (u(s), v(s))\Vert _{L^{p}(\Gamma )\times \widehat{W}^{1, p}(\Gamma )}<\infty . \end{aligned}$$

Next, by (B.34) and (B.37) one has $F_1\in C\big ((0,t), L^p(\Gamma )\big )$. To prove that $F_1$ is integrable we write

$$\begin{aligned}  &   \int _0^t \Vert F_1(s) \Vert _{L^p(\Gamma )}ds \le \int _0^t \big \Vert e^{(\Delta -\sigma )(t-s)}\partial _x \big (f_1(u(s), v(s))\partial _xv(s) \big )\big \Vert _{L^p(\Gamma )} ds\\  &   \quad \le C \int _0^t (t-s)^{-\frac{p+2\gamma -1}{2p}}\Vert (u(s), v(s))\Vert _{L^{p}(\Gamma )\times \widehat{W}^{1, p}(\Gamma )} ds\\  &   \quad \le C \int _0^t (t-s)^{-\frac{p+2\gamma -1}{2p}}ds<\infty , \end{aligned}$$

where we used (B.36).

Letting ${ F(u,v)=f_2(u,v)+\sigma u}$ in Proposition B.3 and *R* be as in (4.17), then (B.16) yields (4.15), and (B.17) and (B.19) yield the continuity of the mapping $(0, t)\ni s\mapsto F_2(s)\in \widehat{W}^{1, p}(\Gamma ).$ To prove the integrability of $F_2$ we employ (B.16) (with $U=(u(s), v(s))$ and $\mathcal {F}(U)=F_2(u(s),v(s))+\sigma u(s)$) to obtain

$$\begin{aligned} \left\| F_2(s)\right\| _{\widehat{W}^{1, p}(\Gamma )}\le C\max \{(t-s)^{-\frac{p+\gamma -1}{2p}}, (t-s)^{-\frac{\gamma -1}{2p}}\} \Vert (u(s), v(s))\Vert _{L^{p}(\Gamma )\times \widehat{W}^{1, p}(\Gamma )} \end{aligned}$$

and then use the dominated convergence theorem together with (4.17) to get

$$\begin{aligned} \int _0^t\max \{(t-s)^{-\frac{p+\gamma -1}{2p}}, (t-s)^{-\frac{\gamma -1}{2p}}\}ds<\infty , \end{aligned}$$

where we used the assumption $p>8\gamma$ and $\gamma \ge 1$, which yields

$$\begin{aligned} 0\le \frac{\gamma -1}{2p}<\frac{p+\gamma -1}{2p}<1. \end{aligned}$$

A similar argument yields (4.16). $\square$

#### Proposition 4.2

Assume Hypothesis 4.1. Let $u_0\in L^p(\Gamma )$ and $v_0\in W^{1,p}(\Gamma )$. Then for $\beta \in (0,1/4), t>0$ one has

4.18 $$\begin{aligned}&(\Phi (u,v;u_0))(t)\in \mathcal {X}^{\beta }_p, \end{aligned}$$

4.19 $$\begin{aligned}&(\Psi (u,v;v_0))(t)\in \mathcal {X}^{{\frac{1}{2}+\beta }}_p,\end{aligned}$$

4.20 $$\begin{aligned}&(\Theta (u;u_0))(t)\in \mathcal {X}^{\beta }_p. \end{aligned}$$

#### Proof

We first prove (4.18). Let us recall $F_1, F_2$ from (4.13), (4.15) and notice that

4.21 $$\begin{aligned} (\Phi (u,v;u_0)(t))=e^{(\Delta -\sigma )t}u_0-\int _{0}^{t} F_1(s)ds+ \int _{0}^{t} F_2(s)ds. \end{aligned}$$

Since $e^{(\Delta -\sigma )t}$ is an analytic semigroup in $L^p(\Gamma )$, we have

4.22 $$\begin{aligned} {e^{(\Delta -\sigma )t}u_0\in \mathcal {X}^{\alpha }_p,\ \alpha >0.} \end{aligned}$$

By Proposition B.5, specifically (B.36), with $F{:}{=}f_1$, we have $F_1(s)\in \mathcal {X}^{\beta }_p$ and

$$\begin{aligned}&\int _0^t \Vert F_1(s) \Vert _{\mathcal {X}^{\beta }_p}ds \le \int _0^t \left\| e^{(\Delta -\sigma )(t-s)}\partial _x \big (f_1(u(s),v(s))\partial _x v(s)\big )\right\| _{\mathcal {X}_p^{\beta }} ds\\&\quad \le C\int _0^t (t-s)^{-\beta -\frac{p+2\gamma -1}{2p} }\Vert (u(s), v(s))\Vert _{L^{p}(\Gamma )\times \widehat{W}^{1, p}(\Gamma )}ds<\infty , \end{aligned}$$

where the last inequality holds due to $\beta +(p+2\gamma -1)/2p<1$. Hence, the second term in (4.21) belongs to $\mathcal {X}^{\beta }_p$. By Proposition B.3, specifically (B.18), with $F(u,v)=f_2(u,v)+\sigma u$, we have $F_2(s)\in \mathcal {X}^{{\frac{1}{2}+\beta }}_p$ and

4.23 $$\begin{aligned} \int _{0}^{t} \Vert F_2(s)\Vert _{\mathcal {X}^{{\frac{1}{2}+\beta }}_p} ds \le C\int _{0}^{t} (t-s)^{{ -\frac{1}{2}}-\beta -\frac{\gamma -1}{2p}} (1+\Vert (u(s), v(s))\Vert _{L^{p}(\Gamma )\times \widehat{W}^{1, p}(\Gamma )}) ds<\infty , \end{aligned}$$

where we used $\frac{1}{2}+\beta +(\gamma -1)/(2p)<1$. Hence, the third term in (4.21) belongs to $\mathcal {X}^{{\frac{1}{2}+\beta }}_p$. It then follows that (4.18) holds.

Next, note that

$$\begin{aligned} (\Psi (u,v;v_0)(t))=e^{(\Delta -\sigma )t}v_0+ \int _{0}^{t} F_3(s)ds. \end{aligned}$$

Then (4.19) follows from the arguments of (4.22) and (4.23).

Finally, (4.20) follows from (4.9), (4.18), and Corollary B.1. $\square$

#### Proposition 4.3

Assume Hypothesis 4.1. Let $\beta \in (0,1/8)$, $u_0\in L^p(\Gamma )$, and $v_0\in W^{1,p}(\Gamma )$. Then one has

4.24 $$\begin{aligned} \Phi (u,v;u_0), \Theta (u;u_0)\in C\left( [0,T], L^p(\Gamma )\right) \cap { C^{\beta }}\left( (0, T], \mathcal {X}_p^{\beta }\right) , \end{aligned}$$

and if $v_0\in \mathcal {X}_p^{\frac{1}{2}+\beta }$, then

4.25 $$\begin{aligned} \Psi (u,v;v_0)\in C\left( [0,T], \widehat{W}^{1,p}(\Gamma )\right) \cap {C^{\beta }}\left( (0, T], \mathcal {X}_p^{\frac{1}{2}+\beta }\right) . \end{aligned}$$

#### Proof

We first prove the Hölder continuity. Recalling (4.21) and (4.14), (4.15), (4.16), let us denote

4.26 $$\begin{aligned}&(I_0(u_0))(t):= e^{(\Delta -\sigma )t}u_0, \ I_1(t)=\int _0^t e^{(\Delta -\sigma )(t-s)}\partial _x(f_1(u(s), v(s))\partial _x v(s))ds,\nonumber \\&I_k(t)=\int _0^t e^{(\Delta -\sigma )(t-s)}(f_k(u(s), v(s))+\sigma u(s)) ds,\ k=2,3, \end{aligned}$$

so that

$$\begin{aligned} (\Phi (u,v;u_0))(t)=(I_0(u_0))(t){-}I_1(t)+I_2(t), \quad (\Psi (u,v;v_0))(t)=(I_0(v_0))(t)+I_3(t). \end{aligned}$$

We will show Hölder continuity of each $I_k$ separately. Throughout the rest of this proof we fix $t\in (0,T)$ and $h\in (0,T-t)$.

For $I_0$ term one has

4.27 $$\begin{aligned} \Vert I_0(u_0)(t+h)-I_0(u_0)(t)\Vert _{\mathcal {X}^{{ \alpha }}_p}&=\Vert (e^{(\Delta -\sigma )h}-I)(\sigma -\Delta )^{{\alpha }} e^{(\Delta -\sigma )t}u_0\Vert _{L^p(\Gamma )}\nonumber \\&\underset{(\hbox {A.11})}{\le } C h^{{\alpha }}\Vert (\sigma -\Delta )^{{\alpha }}e^{(\Delta -\sigma )t} u_0\Vert _{\mathcal {X}^{\alpha }_p}\nonumber \\&\le C h^{\alpha }t^{-2\alpha }\Vert u_0\Vert _{L^p(\Gamma )},\ \alpha \in (0,1), \end{aligned}$$

which implies Hölder continuity of $I_0$ in $\mathcal {X}^\alpha$ with exponent $\alpha$ for any $\alpha \in (0,1)$.

To treat $I_1$, we use Proposition B.5 with $F=f_1$. First, let us notice that

$$\begin{aligned} I_1(t+h)-I_1(t)&= \int _0^t (e^{(\Delta -I)h}-I)e^{(\Delta -I)(t-s)}\partial _x \mathcal {H}(U(s))ds \\&\quad {+} \int _t^{t+h} e^{(\Delta -I)(t-s+h)}\partial _x \mathcal {H}(U(s))ds \end{aligned}$$

where $\mathcal {H}$ is as in Proposition B.5. Then by (B.37) one has

$$\begin{aligned}&\int _0^t \Vert (e^{(\Delta -\sigma )h}-I)e^{(\Delta -\sigma )(t-s)}\partial _x \mathcal {H}(U(s))\Vert _{\mathcal {X}^{\beta }} ds\le \\&\quad \le C\int _0^t h^{\beta }(t-s)^{-2\beta -\frac{p+2\gamma -1}{2p} }\Vert (u(s), v(s))\Vert _{L^{p}(\Gamma )\times \widehat{W}^{1, p}(\Gamma )} ds\le Ch^{\beta }, \end{aligned}$$

and by (B.36)

4.28 $$\begin{aligned}&\int _t^{t+h} \Vert e^{(\Delta -\sigma )(t-s+h)}\partial _x \mathcal {H}(U(s))\Vert _{\mathcal {X}^{\beta }}ds\nonumber \\&\quad \le C\int _t^{t+h} (t-s+h)^{-\beta -\frac{p+2\gamma -1}{2p} }\Vert (u(s), v(s))\Vert _{L^{p}(\Gamma )\times \widehat{W}^{1, p}(\Gamma )}ds\nonumber \\&\quad \le Ch^{-\beta -\frac{p+2\gamma -1}{2p}+1}< Ch^\mathbf{\beta }, \end{aligned}$$

where we used $h\in (0,1)$ and

4.29 $$\begin{aligned} -\beta -\frac{p+2\gamma -1}{2p}+1>\beta >0, \end{aligned}$$

which in turn follows from $\beta \in (0,1/8)$, $p>8\gamma \ge 8$ per Hypothesis 1.1. Therefore

4.30 $$\begin{aligned} \Vert I_1(t+h)-I_1(t)\Vert _{\mathcal {X}^\beta }\le C h^\beta ,\ 0<h\ll 1. \end{aligned}$$

To treat $I_2$ we note

$$\begin{aligned} I_2(t+h)-I_2(t)&= \int _0^t (e^{(\Delta -\sigma )h}-I)e^{(\Delta -I)(t-s)}(f_2(u(s),v(s))+\sigma u)ds\\&\quad { +} \int _t^{t+h} e^{(\Delta -\sigma )(t-s+h)}(f_2(u(s),v(s))+\sigma u(s))ds. \end{aligned}$$

Then Proposition B.3, concretely (B.18), with $F(u,v)=f_2(u,v)+\sigma u$ yields

$$\begin{aligned}&\int _0^t \Vert (e^{(\Delta -\sigma )h}-I)e^{(\Delta -I)(t-s)}(f_2(u(s),v(s))+\sigma u)\Vert _{\mathcal {X}^{{\frac{1}{2}+\beta }}_p} ds\nonumber \\&\quad \underset{(A.11)}{\le }\int _0^t h^\beta \Vert e^{(\Delta -I)(t-s)}(f_2(u(s),v(s))+\sigma u)\Vert _{\mathcal {X}^{\frac{1}{2}+2\beta }_p} ds\nonumber \\&\quad \underset{(\hbox {B.18})}{\le }C \int _0^t h^{\beta }(t-s)^{-\frac{1}{2}-2\beta -\frac{\gamma -1}{2p}}(1+\Vert (u(s), v(s))\Vert _{L^{p}(\Gamma )\times \widehat{W}^{1, p}(\Gamma )})ds<C h^{\beta }, \end{aligned}$$

and

$$\begin{aligned}&\int _t^{t+h} \Vert e^{(\Delta -\sigma )(t-s+h)}(f_2(u(s),v(s))+\sigma u)\Vert _{\mathcal {X}^{\frac{1}{2}+\beta }_p} ds \nonumber \\&\quad \underset{(\hbox {B.18})}{\le } C\int _t^{t+h} (t-s+h)^{-\beta -\frac{1}{2}-\frac{\gamma -1}{2p} }(1+\Vert (u(s), v(s))\Vert _{L^{p}(\Gamma )\times \widehat{W}^{1, p}(\Gamma )})ds< Ch^{\beta }. \end{aligned}$$

Therefore

4.31 $$\begin{aligned} \Vert I_2(t+h)-I_2(t)\Vert _{\mathcal {X}^{\frac{1}{2}+\beta }_p}\le C h^\beta , \, 0<h\ll 1. \end{aligned}$$

By the similar arguments of (4.31), we have

4.32 $$\begin{aligned} \Vert I_3(t+h)-I_3(t)\Vert _{\mathcal {X}^{\frac{1}{2}+\beta }_p}\le C h^\beta , \, 0<h\ll 1. \end{aligned}$$

By (4.27), (4.30), and (4.31), we have $\Phi (u,v;u_0)\in { C^{\beta }}\left( (0,T], \mathcal {X}_p^{\beta }\right)$. By (4.27) with $u_0$ being replaced by $v_0$ and (4.32), we have $\Psi (u,v;v_0)\in {C^{\beta }}\left( (0, T], \mathcal {X}_p^{\frac{1}{2}+\beta }\right)$. By (4.9) and Corollary  B.1, we have $\Theta (u;u_0)\in {C^{\beta }}\left( (0,T], \mathcal {X}_p^{\beta }\right)$.

Next, we prove $\Phi (u,v;u_0),\Theta (u;u_0)\in C\left( [0,T], L^p(\Gamma )\right)$ and if $v_0\in \mathcal {X}_p^{\frac{1}{2}+\beta }$, then $\Psi (u,v;v_0)\in C\big ([0,T], \widehat{W}^{1,p}(\Gamma )\big )$. Note that

$$\begin{aligned} \mathcal {X}^\beta \hookrightarrow L^p(\Gamma ),\quad \mathcal {X}^{\frac{1}{2}+\beta }\hookrightarrow W^{1,p}(\Gamma ). \end{aligned}$$

It then suffices to prove that $\Phi (u,v;u_0)(t)$ and $\Theta (u;u_0)(t)$ are continuous at $t=0$ in $L^p(\Gamma )$, and $\Psi (u,v;v_0)(t)$ is continuous at $t=0$ in $W^{1,p}(\Gamma )$ provided $v_0\in \mathcal {X}_p^{\frac{1}{2}+\beta }$.

Note that4.33By the continuity of $e^{(\Delta -\sigma )t}$ at $t\ge 0$, we have

4.34 $$\begin{aligned} \lim _{t\rightarrow 0} \Vert e^{(\Delta -\sigma )t}u_0-u_0\Vert _{L^p(\Gamma )}=0. \end{aligned}$$

Using Proposition B.5, concretely (B.36), with $F=f_1$ we obtain

4.35 $$\begin{aligned}&\int _{0}^{t} \Vert e^{(\Delta -\sigma )(t-s)}\partial _x(f_1(u(s), v(s))\partial _x v(s))\Vert _{L^p(\Gamma )}ds\nonumber \\&\quad \le C \int _0^t (t-s)^{-\frac{p+2\gamma -1}{2p}}\Vert (u(s), v(s))\Vert _{L^p(\Gamma )\times \widehat{W}^{1, p}(\Gamma )}ds\rightarrow 0\,\, \textrm{as}\,\, t\rightarrow 0. \end{aligned}$$

Using Proposition B.3, specifically (B.16), with $F(u,v)=f_2(u,v)+\sigma u$ one obtains

4.36 $$\begin{aligned}&\int _{0}^{t} \Vert e^{(\Delta -\sigma )(t-s)}(f_2(u(s),v(s))+\sigma u(s))\Vert _{L^p(\Gamma )}ds\nonumber \\&\quad \le C \int _0^t \max ((t-s)^{-\frac{p+\gamma -1}{2p}}, (t-s)^{-\frac{\gamma -1}{2p}})(1+\Vert (u(s), v(s))\Vert _{L^p(\Gamma )\times \widehat{W}^{1, p}(\Gamma )})ds\nonumber \\&\qquad \rightarrow 0\,\, \textrm{as}\,\, t\rightarrow 0. \end{aligned}$$

It then follows that $\Phi (u,v;u_0)(t)$ is continuous at $t=0$ in $L^p(\Gamma )$.

Similarly, it can be proved that $\Theta (u;u_0)(t)$ is continuous at $t=0$ in $L^p(\Gamma )$.

To prove the continuity of $\Psi (u,v;v_0)(t)$ at $t=0$ in $\widehat{W}^{1, p}(\Gamma )$ we first note

$$\begin{aligned}&\Vert (\Psi (u,v;v_0))(t)-(\Psi (u,v;v_0))(0)\Vert _{W^{1,p}(\Gamma )}\\&\quad \le \Vert e^{(\Delta -\sigma )t }v_0-v_0\Vert _{W^{1,p}(\Gamma )}+\left\| \int _0^t e^{(\Delta -\sigma )(t-s)}\Big (f_3(u(s),v(s))+\sigma v(s)\Big )ds\right\| _{W^{1,p}(\Gamma )}. \end{aligned}$$

Furthermore, for $v_0\in \mathcal {X}_p^{\frac{1}{2}+\beta }$ one has

4.37 $$\begin{aligned} \Vert e^{(\Delta -\sigma )t} v_0-v_0\Vert _{W^{1,p}(\Gamma )}&\le C\Vert (\sigma -\Delta )^{\frac{1}{2}+\beta }\big ( e^{(\Delta -\sigma )t}v_0-v_0\big )\Vert _{L^p(\Gamma )}\nonumber \\&= C\Vert e^{(\Delta -\sigma )t} (\sigma -\Delta )^{\frac{1}{2}+\beta }v_0-(\sigma -\Delta )^{\frac{1}{2}+\beta }v_0\Vert _{L^p(\Gamma )}\nonumber \\&\rightarrow 0\quad \textrm{as }\quad t\rightarrow 0, \end{aligned}$$

where in the first inequality we used the embedding $\mathcal {X}_p^{\beta +\frac{1}{2}}\hookrightarrow \widehat{W}^{1, p}(\Gamma )$. By (B.16) with $F(u,v)=f_3(u,v)+\sigma v$, we have

4.38 $$\begin{aligned}&\int _{0}^{t} \Vert e^{(\Delta -\sigma )(t-s)}(f_3(u(s),v(s))+\sigma v(s))\Vert _{W^{1,p}(\Gamma )}ds\nonumber \\&\quad \le C \int _0^t \max ((t-s)^{-\frac{p+\gamma -1}{2p}}, (t-s)^{-\frac{\gamma -1}{2p}})(1+\Vert (u(s), v(s))\Vert _{L^p(\Gamma )\times \widehat{W}^{1, p}(\Gamma )})ds\nonumber \\&\qquad \rightarrow 0\quad \textrm{as}\quad t\rightarrow 0. \end{aligned}$$

Therefore, $\Psi (u,v;v_0)(t)$ is continuous at $t=0$ in $W^{1,p}(\Gamma )$ provided $v_0\in \mathcal {X}_p^{\frac{1}{2}+\beta }$. This completes the proof of the proposition. $\square$

### The fixed point argument and an extension argument

In this subsection, we show existence of mild solutions of (4.1) and (4.2).

For fixed $T>0$, $R>0$ let us introduce

4.39 $$\begin{aligned} B_k(T,R)&:=\left\{ u\in C\big ([0,T], \widehat{W}^{k,p}(\Gamma ) \big ) : \Vert u\Vert _{L^{\infty }_t W^{k,p}_x}:=\max _{t\in [0,T]}\Vert u(t)\Vert _{\widehat{W}^{k,p}(\Gamma )}\le R \right\} ,\nonumber \\&\quad k=0,1. \end{aligned}$$

Below, for example in Proposition  4.4, we consider $B_0(T, R)\times B_1(T, R)$ as a complete metric space with respect to the metric

4.40 $$\begin{aligned} \rho ((u_1, v_1), (u_2,v_2))=\Vert u_1-u_2\Vert _{L^{\infty }_t L^p_x}+\Vert v_1-v_2\Vert _{L^{\infty }_t W^{1,p}_x}, \end{aligned}$$

$$(u_k,v_k)\in B_0(T, R)\times B_1(T, R)$$.

Let us fix $(u_0, v_0)\in L^p(\Gamma )\times \widehat{W}^{1, p}(\Gamma )$, recall (4.5), (4.6), and note that by Proposition 4.3, if $v_0\in \mathcal {X}_p^{\frac{1}{2}+\beta }$, one has

4.41 $$\begin{aligned}&\Lambda (u_0,v_0): C([0, T], L^p(\Gamma )\times \widehat{W}^{1, p}(\Gamma ))\rightarrow C([0, T], L^p(\Gamma )\times \widehat{W}^{1, p}(\Gamma )), \end{aligned}$$

4.42 $$\begin{aligned}&\Theta (u_0): C([0, T], L^p(\Gamma ))\rightarrow C([0, T], L^p(\Gamma )). \end{aligned}$$

#### Proposition 4.4

Assume Hypothesis 1.1. Then for arbitrary $R{ >} \Vert (u_0, v_0)\Vert _{L^p(\Gamma )\times \widehat{W}^{1, p}(\Gamma )}$ there exists $T(R)>0$ such that the following assertions hold:

1. If $v_0\in \mathcal {X}_p^{\frac{1}{2}+\epsilon }$ for some $0<\epsilon \ll 1$, then $\Lambda (u_0,v_0)$ is a contraction mapping on $B_0(T(R), R)\times B_1(T(R), R)$,
2. $\Theta (u_0)$ is a contraction mapping on $B_0(T(R), R)$.

#### Proof

(1). First we note that $\mathcal {X}_p^{\frac{1}{2}+\epsilon }\hookrightarrow \widehat{W}^{1, p}(\Gamma )$, hence, the mapping properties (4.41), (4.42) of $\Lambda , \Theta$ hold as asserted. Next, we show that $\Lambda (u_0,v_0)$ maps $B_0(T(R), R)\times B_1(T(R), R)$ into itself. For $(u,v)\in B_0(T(R), R)\times B_1(T(R), R)$ we have

$$\begin{aligned} \Vert (\Phi (u,v;u_0))(t)\Vert _{L^p(\Gamma )}&\le \Vert e^{(\Delta -\sigma )t}u_0\Vert _{L^p(\Gamma )}\\&\quad + \int _{0}^{t} \Vert e^{(\Delta -\sigma )(t-s)}\partial _x(f_1(u(s), v(s))\partial _x v(s))ds \nonumber \\  &\quad + \int _{0}^{t} \Vert e^{(\Delta -\sigma )(t-s)}(f_2(u(s), v(s))+\sigma u(s))\Vert _{L^p(\Gamma )}ds \end{aligned}$$

and

$$\begin{aligned} \Vert (\Psi (u,v;v_0))(t)\Vert _{\widehat{W}^{1, p}(\Gamma )}&\le \Vert e^{(\Delta -\sigma I)t}v_0\Vert _{\widehat{W}^{1, p}(\Gamma )}\\&\quad + \int _{0}^{t} \Vert e^{(\Delta -\sigma )(t-s)}\left( f_3(u(s), v(s))+\sigma v(s)\right) \Vert _{\widehat{W}^{1, p}(\Gamma )} ds. \end{aligned}$$

Then by (4.34), (4.35), and (4.36), for sufficiently small *T*(*R*) one has

4.43 $$\begin{aligned} \Vert (\Phi (u,v;u_0))(t)\Vert _{L^p(\Gamma )}\le R,\ t\in [0, T(R)]. \end{aligned}$$

Similarly, by (4.37) and (4.38),

$$\begin{aligned} \Vert (\Psi (u,v;v_0))(t)\Vert _{\widehat{W}^{1,p}(\Gamma )}\le R\quad \,\, t\in [0,T(R)] \end{aligned}$$

for *T*(*R*) sufficiently small. Therefore, $\Lambda (u_0,v_0)$ maps $B_0(T, R)\times B_1(T, R)$ into itself for $0<T\ll 1$.

To show that $\Lambda (u_0,v_0): B_0(T(R), R)\times B_1(T, R) \rightarrow B_0(T,R)\times B_1(T,R)$ is a contraction mapping for $0<T\ll 1$, let us first observe that

4.44 $$\begin{aligned}&\Vert (\Phi (u_1,v_1;u_0))(t) - (\Phi (u_2, v_2;u_0))(t)\Vert _{L^p(\Gamma )}\nonumber \\&\qquad +\Vert (\Psi (u_1,v_1;v_0))(t) - (\Psi (u_2, v_2;v_0))(t)\Vert _{\widehat{W}^{1, p}(\Gamma )} \nonumber \\&\quad \le \int _{0}^{t} \Vert e^{(\Delta -\sigma )(t-s)}\partial _x(f_1(u_1(s), v_1(s))\partial _x v_1(s)) \nonumber \\&\qquad - e^{(\Delta -\sigma )(t-s)}\partial _x(f_1(u_2(s), v_2(s))\partial _x v_2(s))\Vert _{L^p(\Gamma )}ds \nonumber \\&\qquad + \int _{0}^{t} \Vert e^{(\Delta -\sigma )(t-s)}(f_2(u_1(s), v_1(s))+\sigma u_1(s))\nonumber \\&\qquad -e^{(\Delta -\sigma )(t-s)}(f_2(u_2(s), v_2(s))+\sigma u_2(s))\Vert _{L^p(\Gamma )}ds\nonumber \\&\qquad +\int _{0}^{t} \Vert e^{(\Delta -\sigma )(t-s)}({f_3}(u_1(s), v_1(s))+\sigma v_1(s))\nonumber \\&\qquad -e^{(\Delta -\sigma )(t-s)}({f_3}(u_2(s), v_2(s))+\sigma v_2(s))\Vert _{\widehat{W}^{1, p}(\Gamma )}ds\nonumber \\&\quad =I+II+III. \end{aligned}$$

Using Proposition B.5, concretely (B.35), with $F=f_1$ one obtains

4.45 $$\begin{aligned} I\le C(R)\int _0^t(t-s)^{-\frac{p+2\gamma -1}{2p}}\Vert (u_1(s), v_1(s))-(u_2(s), v_2(s))\Vert _{{L^p(\Gamma )\times \widehat{W}^{1, p}(\Gamma )}}ds. \end{aligned}$$

Using Proposition B.3, concretely (B.21), with $F(u,v)=f_2(u,v)+\sigma u$ one obtains

4.46 $$\begin{aligned} II\le C(R)\int _0^t(t-s)^{-\frac{p+\gamma -1}{2p}}\Vert (u_1(s), v_1(s))-(u_2(s), v_2(s))\Vert _{{L^p(\Gamma )\times \widehat{W}^{1, p}(\Gamma )}}ds. \end{aligned}$$

Using Proposition B.3, concretely (B.21) again, with $F(u,v)=f_3(u,v)+\sigma v$ one obtains

4.47 $$\begin{aligned} III\le C(R)\int _0^t(t-s)^{-\frac{p+\gamma -1}{2p}}\Vert (u_1(s), v_1(s))-(u_2(s), v_2(s))\Vert _{{L^p(\Gamma )\times \widehat{W}^{1, p}(\Gamma )}}ds. \end{aligned}$$

Combining (4.44), (4.45), (4.46), (4.47) and choosing *T*(*R*) sufficiently small we obtain

4.48 $$\begin{aligned}&\Vert (\Phi (u_1,v_1;u_0))(t) - (\Phi (u_2, v_2;u_0))(t)\Vert _{L^p(\Gamma )} +\Vert (\Psi (u_1,v_1;v_0))(t) \nonumber \\&\qquad - (\Psi (u_2, v_2;v_0))(t)\Vert _{\widehat{W}^{1, p}(\Gamma )} \end{aligned}$$

4.49 $$\begin{aligned}&\quad \le \frac{1}{2} \Vert (u_1(t), v_1(t))-(u_2(t), v_2(t))\Vert _{{L^p(\Gamma )\times \widehat{W}^{1, p}(\Gamma )}},\ t\in [0, T(R)] \end{aligned}$$

as asserted.

Part (2) follows from part (1), (4.9), and Corollary B.1. $\square$

#### Remark 4.1

Recall (4.5) and note that $t\mapsto \Psi (u, v, v_0)(t)$ is considered as a mapping into $\widehat{W}^{1, p}(\Gamma )$. In view of these facts, it is natural to conjecture that the condition $v_0\in \mathcal {X}_p^{\frac{1}{2}+\epsilon }$ in Theorem 4.4 (1) should be relaxed to $v_0\in \widehat{W}^{1, p}(\Gamma )$. We claim, however, that the latter inclusion is not sufficient for $\Psi (u, v, v_0)(t)$ to be continuous in $\widehat{W}^{1, p}(\Gamma )$ at $t=0$. Indeed, $\Psi (u, v, v_0)(t)$, $t>0$ satisfies Neumann–Kirchhoff vertex conditions. If $t\mapsto \Psi (u, v, v_0)(t)$ was continuous in $\widehat{W}^{1, p}(\Gamma )$ at $t=0$ then $v_0$ would have to satisfy the same vertex conditions (since Dirichlet and Neumann traces are continuous with respect to $\widehat{W}^{1, p}(\Gamma )$ norm). Picking $v_0\in \widehat{W}^{1, p}(\Gamma )$ that does not satisfy the Neumann–Kirchhoff vertex conditions yields a contradiction.

That said, our next result shows that the requirement $v_0\in \mathcal {X}_p^{\frac{1}{2}+\epsilon }$ is not necessary for existence of mild solutions of (4.1), (4.2) that are continuous in $\widehat{W}^{1, p}(\Gamma )$ for strictly positive time $t\in (0, T)$ but not $t=0$, in which case the continuity is claimed only with respect $L^{p}(\Gamma )$ norm.

#### Proposition 4.5

Assume Hypothesis 1.1. Let $\beta \in (0, 1/8)$ and suppose that the initial data satisfies $\Vert (u_0, v_0)\Vert _{L^p(\Gamma )\times \widehat{W}^{1, p}(\Gamma )}< R$.

1. There exists $T>0$ such that integral equation (4.1) has a unique solution $(u(t),v(t))=(u(t;u_0,v_0),v(t;u_0,v_0))$ satisfying
  4.50 $$\begin{aligned}&(u,v) \in { C([0, T], L^p(\Gamma )\times L^p(\Gamma ))\cap C^{\beta }((0, T], \mathcal {X}^{\beta }_p \times \mathcal {X}_p^{\frac{1}{2}+\beta })},\nonumber \\&\sup _{t\in (0,T]}\Vert u(t), v(t)\Vert _{{L^p(\Gamma )}\times \widehat{W}^{1,p}(\Gamma )}\le R. \end{aligned}$$
2. There is $T>0$ such that (4.2) has a unique solution $(u(t),v(t))=(u(t;u_0),v(t;u_0))$ satisfying
  4.51 $$\begin{aligned}&u\in C([0, T], L^p(\Gamma ))\cap C^{\beta }((0, T], \mathcal {X}^{\beta }_p),\nonumber \\&\quad \max _{t\in [0,T]}\Vert u(t)\Vert _{L^p(\Gamma )}\le R. \end{aligned}$$

#### Proof

(1) First, note that $\mathcal {X}_p^{\frac{1}{2}+\beta }$ is dense in $\widehat{W}^{1,p}(\Gamma )$. Hence there is a sequence $\{v_n\}\subset \mathcal {X}_p^{\frac{1}{2}+\beta }$ such that

$$\begin{aligned} \Vert v_n- v_0\Vert _{\widehat{W}^{1,p}(\Gamma )}\rightarrow 0\quad \textrm{as}\quad n\rightarrow \infty . \end{aligned}$$

Then there is $N\in \mathbb {N}$ such that

$$\begin{aligned} \Vert (u_0, v_n)\Vert _{L^p(\Gamma )\times \widehat{W}^{1, p}(\Gamma )}< R,\,\, n\ge N. \end{aligned}$$

By Proposition 4.4, there is $T=T(R)>0$ such that $\{\Lambda (u_0,v_n)\}_{n\ge N}$ is a family of uniform contractions on $B_0(T, R)\times B_1(T, R)$. Then by fixed point theorem, the integral equation (4.1) with $v_0$ being replaced by $v_n$ ($n\ge N$) has a unique solution $(u_n(t),v_n(t))=(u(t;u_0,v_n),v(t;u_0,v_n))$ satisfying

$$\begin{aligned}&(u_n,v_n)\in C([0, T], L^p(\Gamma )\times \widehat{W}^{1, p}(\Gamma ))\cap { C^{\beta }}\left( (0, T], \mathcal {X}^{\beta }_p\times \mathcal {X}^{{\frac{1}{2}+\beta }}_p\right) ,\\&\quad \max _{t\in [0,T]}\Vert u_n(t), v_n(t)\Vert _{{L^p(\Gamma )}\times \widehat{W}^{1,p}(\Gamma )}\le R \end{aligned}$$

for $$n\ge N$$.

Next, let us note that^9^

$$\begin{aligned} \mathcal {X}^{\beta }_p\hookrightarrow \widehat{C}({{\overline{\Gamma }}})\hookrightarrow L^p(\Gamma )\quad \textrm{and}\quad \mathcal {X}^{\frac{1}{2}+\beta }_p\hookrightarrow \widehat{C}^1({{\overline{\Gamma }}}) \hookrightarrow W^{1,p}(\Gamma ). \end{aligned}$$

By Arzela-Ascoli Theorem there exists $(u^*,v^*)\in C((0,T],L^p(\Gamma )\times \widehat{W}^{1, p}(\Gamma ))$ and a subsequence $(u_{n_k}(t),v_{n_k}(t))$ such that

$$\begin{aligned} \lim _{n_k\rightarrow \infty } (u_{n_k}(t),v_{n_k}(t))=(u^*(t),v^*(t)) \quad \textrm{in}\quad L^p(\Gamma )\times W^{1,p}(\Gamma ), \end{aligned}$$

locally uniformly in (0, *T*], and

$$\begin{aligned} \sup _{t\in (0,T]}\Vert (u^*(t),v^*(t))\Vert _{L^p(\Gamma )\times W^{1,p}(\Gamma )}\le R. \end{aligned}$$

Note also that

$$\begin{aligned} \lim _{n\rightarrow \infty } e^{(\Delta -\sigma )t}v_n=e^{(\Delta -\sigma )t}v_0 \quad \textrm{in}\quad L^p(\Gamma ) \end{aligned}$$

uniformly in $t\in [0,T]$. It then follows that $(u^*(t),v^*(t))$ satisfies

$$\begin{aligned} u^*(t)&=e^{(\Delta -\sigma )t}u_0-\int _{0}^{t} e^{(\Delta -\sigma )(t-s)}\partial _{x}\big (f_1(u^*(s), v^*(s))\partial _xv^*(s)\big )ds\\&\quad + \int _{0}^{t} e^{(\Delta -\sigma )(t-s)}(f_2(u^*(s), v^*(s))+\sigma u^*(s))ds,\\ v^*(t)&=e^{(\Delta -\sigma I)t}v_0+ \int _{0}^{t} e^{(\Delta -\sigma )(t-s)}\left( f_3(u^*(s), v^*(s))+\sigma v^*(s)\right) ds \end{aligned}$$

for $t\in (0,T]$. Therefore, $(u(t;u_0,v_0),v(t;u_0,v_0)){:}{=}(u^*(t),v^*(t))$ is a solution of the integral equation (4.1) satisfying (4.50). The uniqueness of such solutions follows readily from the estimates similar to (4.44), (4.49).

(2) The fixed point theorem for contraction mapping $\Theta (u_0): B_0(T, R)\rightarrow B_0(T, R)$ for $0<T\ll 1$, as in Proposition 4.4, yields (4.51). $\square$

#### Theorem 4.1

Assume Hypothesis 1.1 and let $\beta \in (0, 1/8)$.

1. Let $u_0\in L^p(\Gamma )$. Then there exists $T_{\max }=T_{\max }(u_0)\in (0,\infty ]$ such that integral equation (4.2) has a unique solution $(u(t),v(t))=(u(t;u_0),v(t;u_0))$ satisfying
  4.52 $$\begin{aligned} u(\cdot )\in C([0, T_{\max }), L^p(\Gamma ))\cap C^{\beta }((0, T_{\max }), \mathcal {X}^{\beta }_p),\ u(0;u_0)=u_0. \end{aligned}$$
  If $T_{\max }<\infty$ then $\limsup \limits _{t\rightarrow T_{\max }^-}\Vert u(t)\Vert _{L^p(\Gamma )}=\infty$. Moreover, for arbitrary $r\ge 1$ and $\nu <\beta$,
  4.53 $$\begin{aligned} u\in C^{{ \beta }}((0, T_{\max }), \widehat{C}^{\nu }(\overline{\Gamma }))\cap C^{\beta }((0, T_{\max }), \mathcal {X}^{\beta }_r), \end{aligned}$$
  (hence (1.10) holds), and
  4.54 $$\begin{aligned} v\in C^{\beta }((0, T_{\max }), \widehat{C}^{2+\nu }(\overline{\Gamma })). \end{aligned}$$
2. Let $(u_0, v_0)\in L^p(\Gamma )\times \widehat{W}^{1, p}(\Gamma )$. Then there exists $T_{\max }=T_{\max }(u_0,v_0)\in (0,\infty ]$ such that the integral equation (4.1) has a unique solution
  $$\begin{aligned} (u(t),v(t))=(u(t;u_0,v_0),v(t;u_0,v_0)) \end{aligned}$$
  satisfying
  4.55 $$\begin{aligned} (u,v) \in C([0, T_{\max }), L^p(\Gamma )) \times L^p(\Gamma ) )\cap C^{0,\beta }((0, T_{\max }), \mathcal {X}^{\beta }_p\times \mathcal {X}^{{\frac{1}{2}+\beta }}_p), \end{aligned}$$
  with $u(0;u_0)=u_0, v(0;v_0)=v_0$. If $T_{\max }<\infty$ then
  4.56 $$\begin{aligned} \limsup \limits _{t\rightarrow T_{\max }^-}\Vert (u(t),v(t))\Vert _{L^p(\Gamma )\times \widehat{W}^{1, p}(\Gamma )}=\infty . \end{aligned}$$
  Furthermore, for $r\ge 1$, $\nu < \beta$, *u*, *v* satisfy
  4.57 $$\begin{aligned} u\in C^{\beta }((0, T_{\max }), \mathcal {X}^{\beta }_r)\cap C^{\beta }((0, T_{\max }), \widehat{C}^{\nu }(\overline{\Gamma })), \end{aligned}$$
  and
  4.58 $$\begin{aligned} v\in C^{1+\beta } ((0,T_{\max }), \mathcal {X}^{\beta }_r)\cap C^{\beta }((0, T_{\max }), \widehat{C}^{2+\nu }(\overline{\Gamma })) \end{aligned}$$
  (hence (1.12) and (1.13) hold).

#### Proof

(1) First of all, the existence of solution (4.52) to (4.2) for large *p* follows from Proposition 4.5 and standard extension arguments.

Next, let us prove (4.53) for arbitrary $r\ge 1$, which together with (4.52) implies (1.10). Due to compactness of the graph, it suffices to verify the assertion for $r>p$. To that end, combining (A.4) and (A.8) we note that for any $q> p$, $\beta \in (0,\frac{1}{8})$, and $\theta \in (0,1)$ with $\frac{1}{q}>\frac{1}{p}-\frac{1}{4}$, one has

$$\begin{aligned} u(\cdot ;u_0)\in C^{\beta }((0,T_{\max }),\widehat{L}^{q}(\Gamma )). \end{aligned}$$

Next, for the partition $p={\tilde{p}}_0<{\tilde{p}}_1<{\tilde{p}}_2<\cdots <{\tilde{p}}_N=r$ such that

$$\begin{aligned} \frac{1}{{\tilde{q}}_i}>\frac{1}{{\tilde{q}}_{i-1}}-\frac{1}{4},\quad i=1,2,\cdots ,N, \end{aligned}$$

one obtains

4.59 $$\begin{aligned} u(\cdot ;u_0)\in C^{{ \beta }}((0,T_{\max }),\widehat{L}^{{\tilde{q}}_i}(\Gamma )),\quad i=1,2,\cdots ,N. \end{aligned}$$

Hence, $u(\cdot ;u_0)\in C^{\beta }((0,T_{\max }),\widehat{L}^{r}(\Gamma ))$ for arbitrary $r\ge 1$. Then using $u(\varepsilon , u_0)$, $\varepsilon >0$ as an initial condition and employing (4.52) with $p=r$ we obtain

4.60 $$\begin{aligned} u(\cdot ; u_0)\in C^{\beta }((\varepsilon , T_{\max }(u_0, v_0)), \mathcal {X}^{\beta }_r), \end{aligned}$$

as asserted. Finally, (A.9) and (4.60) with $0<\nu <\frac{1}{8}$, $\theta \in (0,1)$, $\beta \in (\nu ,\frac{1}{8})$, and $r>1$ such that $\nu <2\beta \theta -r^{-1}$, one obtains $u(\cdot ;u_0)\in C^{\beta }((0,T_{\max }),\widehat{C}^\nu (\overline{\Gamma })).$ (4.53) then follows.

Now, we prove (4.54). Recall that $v(t;u_0)= -(\Delta -\sigma )^{-1}f(u(t;u_0))$. By (4.53) and Proposition B.4, we have

$$\begin{aligned} v\in C^{\beta }((0, T_{\max }), \widehat{W}^{2,r}(\Gamma )). \end{aligned}$$

By (A.5), we have

4.61 $$\begin{aligned} \widehat{W}^{2,r}(\Gamma )\hookrightarrow \widehat{C}^{{\tilde{\nu }}} ({\overline{\Gamma }}) \quad {\tilde{\nu }} <2-\frac{1}{r}. \end{aligned}$$

It then follows that

4.62 $$\begin{aligned} v\in C^{\beta }((0, T_{\max }), \widehat{C}^{{\tilde{\nu }}}({\overline{\Gamma }}))\quad \forall \,{\tilde{\nu }} <2-\frac{1}{r}. \end{aligned}$$

Note that

$$\begin{aligned} v_{xx}=v-f(u). \end{aligned}$$

This together with (4.53) and (4.62) implies (4.54).

(2) First, as in (1), the existence of solution (4.55)) to (4.1) for large *p* follows from Proposition 4.5 and standard extension arguments. Property (4.57) follows from the arguments of (4.53).

Next, we prove (4.58). To this end, we first prove

4.63 $$\begin{aligned} v\in C^{\beta } ((0,T_{\max }), \widehat{W}^{2,p}(\Gamma ))\cap C^{1+\beta } ((0,T_{\max }), \mathcal {X}^{\beta }_p). \end{aligned}$$

For arbitrary $[\kappa ,\tau ]\subset (0, T_{\max }(u_0, v_0))$, consider an auxiliary non-homogeneous Cauchy problem

4.64 $$\begin{aligned} w_t=w_{xx}+f_3(u,v), w(\tau )=v(\tau ), t\ge \tau . \end{aligned}$$

We claim this equation posses unique strong solution satisfying $w\in C^{1+\beta }([\kappa ,\tau ], \mathcal {X}^{\beta }_p)$ and that this solution coincides with *v*. To prove the former claim let us note that $F(s){:}{=}f_3(u(s), v(s))$ is a Hölder continuous mapping from $[\kappa ,\tau ]$ to $L^p(\Gamma )$ due to (4.55) and $\mathcal {X}^{\beta }_p\hookrightarrow C^{\nu }$ for some $\nu >0$. Hence, by Theorem C.1 item (2), a unique strong solution of (4.64) exists and satisfies the integral equation

4.65 $$\begin{aligned} w(t)=e^{\Delta (t-\tau )}{ w({\tau })}+\int _{\tau }^te^{\Delta (t-s-\tau )}f_3(u(s), v(s))ds, t\in [\tau , \kappa ]. \end{aligned}$$

Hence, to prove $w\in C^{1+\beta }([\kappa ,\tau ], \mathcal {X}^{\beta }_p)$ it suffices to show that the integral term, as a function of *t*, in the right-hand side above belongs to $C^{1+\beta }([\kappa ,\tau ], \mathcal {X}^{\beta }_p)$. This, in turn, follows from Hölder continuity of *F* combined with [26, Lemma 3.5.1]. Therefore, (4.64) shows that the mapping $t\mapsto w_{xx}(t)$ belongs to $C^{\beta }([\kappa ,\tau ], L^p(\Gamma ))$, hence, $w\in C^{\beta }([\kappa ,\tau ], \widehat{W}^{2,p}(\Gamma ))$. Since both *v* and *w* satisfy (4.65), by Theorem C.1, they coincide, thus (4.63) holds as asserted.

By (4.57) and (4.63), repeating the arguments of (4.63), we have

4.66 $$\begin{aligned} v\in C^{\beta } ((0,T_{\max }), \widehat{W}^{2,r}(\Gamma ))\cap C^{1+\beta } ((0,T_{\max }), \mathcal {X}^{\beta }_r)\quad \forall \, r>1. \end{aligned}$$

Note that

$$\begin{aligned} v_{xx}=v_t-f_3(u,v). \end{aligned}$$

This together with (4.66) and (4.61) implies (4.58). $\square$

### Proof of Theorem 1.1

In this subsection, we prove Theorem 1.1.

#### Proof of Theorem 1.1

We divide the proof of the theorem into three steps.

**Step 1.** In this step, let (*u*(*t*), *v*(*t*)) be as in Theorem 4.1, that is, (*u*(*t*), *v*(*t*)) is the solution to (4.2) if $\tau =0$ and to (4.1) if $\tau =1$. In both cases one has

4.67 $$\begin{aligned} u(t)&= e^{(\Delta -\sigma )(t-\kappa )}u(\kappa )-\int _{\kappa }^{t} e^{(\Delta -\sigma )(t-\kappa -s)}\partial _x\left( f_1(u(s), v(s))\partial _x v(s)\right) ds\nonumber \\&\quad +\int _{\kappa }^{t} e^{(\Delta -\sigma )(t-\kappa -s)}\left( \frac{f_2(u(s),v(s))+\sigma u(s)}{u(s)}\right) u(s), t\in [\kappa , T_{\max }), \end{aligned}$$

with the integral convergent in $L^q(\Gamma )$ for arbitrary $q\in [1, p]$, $\kappa >0$.

Let $\kappa \in (0,T_{max})$. We prove the existence and uniqueness of classical solutions of

4.68 $$\begin{aligned} {\left\{ \begin{array}{ll} u_t=(\Delta -\sigma )u-a(t, x)\partial _xu+b(t,x)u,\quad x\in \Gamma \\ \sum \limits _{\vartheta \sim e} \partial _{\nu }u_e(\vartheta )=0,\ u_e(\vartheta )=u_{e'}(\vartheta ), v_e(\vartheta )=v_{e'}(\vartheta ), e\sim \vartheta , e'\sim \vartheta ,\\ u(\kappa ,x)=u(\kappa )(x), \end{array}\right. } \end{aligned}$$

where $a(t,x)=a(t)(x)$ and $b(t,x)=b(t)(x)$ and *a*(*t*), *b*(*t*) are given as in (4.12). By Theorem 4.1, we have

$$\begin{aligned}  &   a(t,\cdot ),b(t,\cdot )\in \widehat{C}({\overline{\Gamma }})\,\,\,\textrm{for}\,\, t\in (0,T_{\max }),\\  &   a(t,\cdot ), b(t,\cdot )\in C^{\beta }((0,T_{\max }), \widehat{C}({\overline{\Gamma }})),\, 0<\beta <\frac{1}{8}, \end{aligned}$$

and

$$\begin{aligned} u(\kappa )\in \mathcal {X}_r^\beta ,\, r\ge 1,\,\, 0<\beta <\frac{1}{8}, \end{aligned}$$

Let $\Psi (t; u(\kappa )){:}{=}T(t,\kappa )u(\kappa )$, where the evolution operator $T(t, \kappa )$ is as in Theorem 1.4. We will show that $u(t)=\Psi (t, u(\kappa ))$, $t\in [\kappa , \tau ]$, for every $\kappa \in (0,T_{\max })$ and $\tau$ sufficiently close to $\kappa$. To that end, abbreviating $\Psi (s)=\Psi (s; u(\kappa ))$ we note that for $t>\kappa$ one has

4.69 $$\begin{aligned} \Psi (t)=e^{(\Delta -I)(t-\kappa )}u(\kappa )-\int _{\kappa }^{t}e^{(\Delta -\sigma )(t-\kappa -s)}a(s)\partial _s\Psi (s)ds+\int _{\kappa }^{t}e^{(\Delta -\sigma )(t-\kappa -s)}b(s)\Psi (s)ds \end{aligned}$$

Let us also notice that

4.70 $$\begin{aligned}&a\partial _s\Psi =\partial _x\left( f_1(\Psi ,v)\partial _xv\right) -\frac{\partial _vf_1(\Psi ,v)(\partial _xv)^2+f_1(\Psi ,v)\partial _{xx}v}{\Psi } \Psi , \end{aligned}$$

4.71 $$\begin{aligned}&b\Psi =\left( \frac{f_2(u,v)+\sigma u}{u}\right) \Psi -\frac{\partial _vf_1(u,v)(\partial _xv)^2+f_1(u,v)\partial _{xx}v}{u} \Psi . \end{aligned}$$

Combining (4.67), (4.69), (4.70) we arrive at

4.72 $$\begin{aligned} u(t)-\Psi (t)&=\int _{\kappa }^t e^{(\Delta -\sigma )(t-\kappa -s)}\partial _x\big (f_1(\Psi (s), v(s))-f_1(u(s), v(s))\partial _xv(s)\big )ds\nonumber \\&\quad +\int _{\kappa }^t e^{(\Delta -\sigma )(t-\kappa -s)}\left( \frac{f_2(u(s),v(s))+\sigma u(s)}{u(s)}\right) (u(s)-\Psi (s))ds\nonumber \\&\quad +\int _{\kappa }^t e^{(\Delta -\sigma )(t-\kappa -s)}\left( \frac{\partial _vf_1(u,v)}{u}-\frac{\partial _vf_1(\Psi ,v)}{\Psi } \right) (\partial _xv)^2\Psi (s)ds\nonumber \\&\quad +\int _{\kappa }^t e^{(\Delta -\sigma )(t-\kappa -s)}\left( \frac{f_1(u,v)}{u}-\frac{f_1(\Psi ,v)}{\Psi } \right) \partial _{xx}v\Psi (s)ds\nonumber \\&=I+II+III+IV. \end{aligned}$$

Our next objective is to estimate $L^q(\Gamma )$ norm of both sides of the above identity and employ Gronwall-type inequality from Proposition C.1. To estimate each term we use Proposition B.2 with special choices of *F* as listed below

4.73 $$\begin{aligned}&\text {I term:}\ F(u,\phi )=f_1(u,\phi ), \phi {:}{=}v, \psi =\partial _xv, \end{aligned}$$

4.74 $$\begin{aligned}&\text {II term:}\ F(u,\phi )= u, \psi =(f_2(u(s),v(s))+\sigma u(s))(u(s))^{-1}, \end{aligned}$$

4.75 $$\begin{aligned}&\text {III term:}\ F(u,\phi )= u^{-1}\partial _vf_1(u, \phi ),\ \phi =v, \psi =(\partial _x v)^2\Psi ,\end{aligned}$$

4.76 $$\begin{aligned}&\text {IV term:}\ F(u,\phi )= \partial _vf_1(u, \phi ),\ \phi =v,\ \psi =(\partial _x v)^2\Psi , \end{aligned}$$

to arrive at

4.77 $$\begin{aligned} \Vert u(t)-\Psi (t)\Vert _{L^q(\Gamma )}\le C\int _{\kappa }^t(t-\kappa -s)^{-\alpha }\Vert u(s)-\Psi (s)\Vert _{L^q(\Gamma )}ds,\ C>0, \alpha \in (0,1). \end{aligned}$$

Thus by Proposition C.1$u(t)=\Psi (t)$ for $t\ge \kappa$ with sufficiently small $|t-\kappa |$. Hence, by Theorem 1.4, $u=\Psi \in \widehat{C}^{1,2}([\kappa , \tau ]\times \overline{\Gamma })$.

**Step 2.** The uniqueness follows from the fact that classical solutions satisfy the integral equation (1.5).

**Step 3.** In this step, we prove that if $u_0\ge 0$ and $v_0\ge 0$, then $u(t)\ge 0$ and $v(t)\ge 0$ for all $t\in (0,T_{\max })$. We prove this for the case $\tau >0$. The case $\tau =0$ can be proved similarly. Note that for any $u_0\in L^p(\Gamma )$, $v_0\in \widehat{W}^{1,p}(\Gamma )$ with $u_0\ge 0, v_0\ge 0$, there are $u_n\in C({\overline{\Gamma }})$, $v_n\in C^1({\overline{\Gamma }})$ such that $u_n\ge 0,v_n\ge 0$ and

$$\begin{aligned} \lim _{n\rightarrow \infty } \Big (\Vert u_n-u_0\Vert _{L^p(\Gamma )}+\Vert v_n-v_0\Vert _{\widehat{W}^{1,p}(\Gamma )}\Big )=0. \end{aligned}$$

It is not difficult to see that

$$\begin{aligned}  &   \lim _{n\rightarrow \infty } \Vert u(t;u_n,v_0)-u(t;u_0,v_0)\Vert _{L^p(\Gamma )}\\  &   \quad +\Vert v(t;u_n,v_n)-v(t;u_0,v_0)\Vert _{L^p(\Gamma )}=0\quad \forall \, t\in [0,T_{\max }). \end{aligned}$$

By Theorem C.2 (3), we have $u(t;u_n.v_n)\ge 0$ and $v(t;u_0,v_n)\ge 0$. It then follows that $u(t;u_0,v_0)\ge 0$ and $v(t;u_0,v_0)\ge 0$. $\square$

## Global well-posedness of logistic-type Keller–Segel systems

The main goal of this section is to provide the proof of Theorem 1.2. Let us outline the strategy of the proof.

### Outline of the proof of Theorem 1.2

Employing local existence results from Sect. 4 yields classical solutions of (1.14) on the maximal existence time interval $[0,T_{\max })$.^10^ To prove global well-posedness, i.e., $T_{\max }=\infty$ we rule out finite time blow-up of $t\mapsto (u(t), v(t))$ in ${L^q(\Gamma )\times \widehat{W}^{1,q}(\Gamma )}$ for all $q\ge 1$. This is achieved by induction in *q*. The base case $q=1$ is proved in Lemma 5.1 by deriving from (1.14) the following differential inequality

5.1 $$\begin{aligned} \frac{d}{dt}\int _\Gamma u(t;u_0)dx\le k|\Gamma |+l\int _\Gamma u(t;u_0)dx-\frac{m}{|\Gamma |^{\varepsilon }}\left( \int _\Gamma u(t;u_0)dx\right) ^{1+\varepsilon }. \end{aligned}$$

Then a bound on $\Vert u(t)\Vert _{L^{1}(\Gamma )}$ follows from the comparison principle for ODEs. To make the induction step, we derive the following inequality

5.2 $$\begin{aligned} \frac{d}{dt}\int _{\Gamma } u^{n}dx\le C\left( 1+\int _{\Gamma } u^ndx-\left( \int _\Gamma u^{n}dx\right) ^{1+1/n}\right) , \end{aligned}$$

where $C>0$ depends on $\Vert (u(t), v(t))\Vert _{L^{n-1}(\Gamma )\times \widehat{W}^{1,n-1}(\Gamma )}$. Using the induction hypothesis and the comparison principle we then obtain a bound on $\Vert (u(t), v(t))\Vert _{L^{n}(\Gamma )\times \widehat{W}^{1,n}(\Gamma )}$. For $\tau =0$ and either $m>0$ or $m=k=l=0$ we show that the above norms are uniformly bounded with respect to *t*. This argument is carefully carried out in Lemma 5.1.

To prove global boundedness in case $\tau =0$ with $m>0$ or $m=k=l=0$ we use the integral equation

5.3 $$\begin{aligned} u(t)&=e^{t(\Delta -\sigma ) }u_0+\int _{0}^{t} \chi e^{(t-s)(\Delta -\sigma )}\partial _x\left( u(s)\partial _xv(s)\right) ds\nonumber \\&\quad +\int _{0}^{t} e^{(t-s)(\Delta -\sigma )}(g(u(s))+\sigma u(s))ds. \end{aligned}$$

The estimates derived in Lemmas 5.1 and 5.2 together with (5.3) yield bound on $\Vert u(t)\Vert _{\mathcal {X}^{\beta }_q}$ in terms of $\Vert (u(t), v(t))\Vert _{L^{q}(\Gamma )\times \widehat{W}^{1,q}(\Gamma )}$. Combining this bound with the embedding $\mathcal {X}^{\beta }_{q}\hookrightarrow L^{\infty }(\Gamma )$ gives uniform boundedness of solutions. This argument is spelled out at the end of current section.

### Norm estimates for solutions of the logistic-type model.

#### Lemma 5.1

Assume hypotheses of Theorem 1.2 and let $T>0$.

1. If $\tau =0$ then one has
  5.4 $$\begin{aligned} \sup \limits _{t\in [0,\min \{T,T_{\max }(u_0)\})} \Vert u(t;u_0)\Vert _{L^{1}(\Gamma )}\le { M_0}, \end{aligned}$$
  where
  5.5 $$\begin{aligned} M_{0}{ =M_0(u_0,k,l,m,T)}{:}{=} {\left\{ \begin{array}{ll} C(u_0, k,l,m), & \textrm{if}\,m>0,\\ e^{{ l{\hat{T}}}}\int u_0dx+\frac{k|\Gamma |}{l}\Big (e^{{ l{\hat{T}}}}-1\Big ) ,&  \textrm{if}\,\, m=0,\,\, { l}>0,\\ \int _\Gamma u_0(x)dx+k|\Gamma | { {\hat{T}}},&  \textrm{if}\,\, m={ l}=0, \end{array}\right. } \end{aligned}$$
  for some *T*-independent constant $C(u_0, k,l,m)>0$, ${\hat{T}}=\min \{T,T_{\max }(u_0)\}$.
2. If $\tau >0$ then $0\le \int u(t;u_0, v_0)dx\le { M_0}$, for all $t\in [0,\min \{T,T_{\max }(u_0, v_0)\})$ and ${ M_0}$ as above. Moreover, for arbitrary $q\ge 1$ there is $M_1=M_1(u_0, v_0, k,l,m, { T})>0$ such that
  5.6 $$\begin{aligned} \sup \limits _{t\in [0,\min \{T,T_{\max }(u_0, v_0)\})}\Vert v(t;u_0,v_0)\Vert _{\widehat{W}^{1,q}(\Gamma )}\le M_1. \end{aligned}$$

#### Proof

(1) Integrating both sides of the first equation in (1.14) over $\Gamma$ and using (1.17) we obtain

$$\begin{aligned} \frac{d}{dt}\int _\Gamma u(t;u_0)dx\le k|\Gamma |+l\int _\Gamma u(t;u_0)dx-m\int _\Gamma u^{1+\varepsilon }(t;u_0)dx. \end{aligned}$$

This inequality together with

$$\begin{aligned} \int _\Gamma u^{1+\varepsilon }(t;u_0)dx\ge {|\Gamma |}^{-\varepsilon }\left( \int _\Gamma u(t;u_0)dx\right) ^{1+\varepsilon } \end{aligned}$$

yields (5.1).

Then (5.1) and comparison principle for scalar ODEs yield the following assertions. If $m>0$, then

5.7 $$\begin{aligned} \int _\Gamma u(t;u_0)dx\le \max \left\{ \int _\Gamma u_0dx, r(k,l,m)\right\} , t\in [0,\min \{T,T_{\max }(u_0)\}), \end{aligned}$$

where $r(k,l,m)>0$ is the positive zero of $\varphi (x){:}{=} k|\Gamma |+lx-m|\Gamma |^{-\varepsilon }x^{1+\varepsilon }$. If $m=0$ and $l>0$ then

5.8 $$\begin{aligned} \int _\Gamma u(t;u_0)dx\le e^{lt}\int _{\Gamma }u_0dx+\frac{k|\Gamma |}{l}\Big (e^{lt}-1\Big ),\ t\in [0,\min \{T,T_{\max }(u_0)\}). \end{aligned}$$

If $$m=l=0$$, then

5.9 $$\begin{aligned} \int _\Gamma u(t;u_0)\le \int _\Gamma u_0dx+k|\Gamma |t,\ t\in [0,\min \{T,T_{\max }(u_0)\}). \end{aligned}$$

The assertion (1.17) follows from (5.7), (5.8), and (5.9).

(2). The first assertion is proved as in (1). Let us prove the second assertion. Integrating the second equation in (1.14) we obtain

5.10 $$\begin{aligned} \frac{d}{dt}\int _\Gamma v(t;u_0, v_0)dx\le \int _\Gamma u(t;u_0, v_0)dx-\int _\Gamma v(t;u_0, v_0)dx\le M_0-\int _\Gamma v(t)dx, \end{aligned}$$

hence, for some $C=C(v_0)>0$ one has

5.11 $$\begin{aligned} \int _\Gamma v(t;u_0, v_0)dx\le \max \left\{ \int _{\Gamma } v_0 dx, M_0 \right\} {:}{=}{ M_0'}. \end{aligned}$$

Next, let us rewrite the second equation in (1.14) as follows

5.12 $$\begin{aligned} v_t=v_{xx}-(1+\sigma _0)v+u+\sigma _0v, \end{aligned}$$

where $\sigma _0>0$ is as in Corollary 2.1. Then denoting $\omega {:}{=}1+\sigma _0$ one obtains

$$\begin{aligned} \partial _xv(t)=\partial _xe^{(\Delta -\omega )t}v_0+\int _{0}^{t}\partial _xe^{(\Delta -\omega )(t-s)}\big (u(s)+\sigma _0v(s)\big )ds. \end{aligned}$$

Since $\omega >\sigma _0$ we can employ Corollary 2.1 to get some $\delta \in (0, \omega )$ and a *T*-independent constant $C=C(\omega , p, \Gamma , \delta )>0$ for which one has

$$\begin{aligned}&\Vert \partial _x v(t)\Vert _{L^q(\Gamma )} \le \Vert \partial _xe^{(\Delta -\omega )t}v_0\Vert _{L^q(\Gamma )} +\int _{0}^{t}\Vert \partial _xe^{(\Delta -\omega )(t-s)}\big (u(s)+\sigma _0v(s))\Vert _{L^q(\Gamma )}ds\\&\quad \le Ct^{-\frac{1}{2}-\frac{1}{2}\left( 1-\frac{1}{q}\right) }e^{-\delta t}\Vert v_0\Vert _{L^{1}(\Gamma )}\\&\qquad +C\sup _{t\in [0,\min \{T,T_{\max }\})}( \Vert u(t)\Vert _{L^1(\Gamma )}+\Vert v(t)\Vert _{L^1(\Gamma )}) \int _{0}^{t}(t-s)^{-\frac{1}{2}-\frac{1}{2}\left( 1-\frac{1}{q}\right) }e^{-\delta (t-s)} ds\\&\quad \le Ct^{-\frac{1}{2}-\frac{1}{2}\left( 1-\frac{1}{q}\right) }e^{-\delta t}\Vert v_0\Vert _{L^{1}(\Gamma )}+C\sup _{t\in [0,\min \{T,T_{\max }\})}( \Vert u(t)\Vert _{L^1(\Gamma )}+\Vert v(t)\Vert _{L^1(\Gamma )}) \end{aligned}$$

for all $t\in [0, \min \{T,T_{\max }\})$. Combining this with (5.4), (5.11) we get

5.13 $$\begin{aligned} \Vert \partial _x v(t)\Vert _{L^q(\Gamma )} \le Ct^{-\frac{1}{2}-\frac{1}{2}\left( 1-\frac{1}{q}\right) }e^{-\delta t}\Vert v_0\Vert _{L^{1}(\Gamma )}+C(1+{ M_0'}), \end{aligned}$$

for some *T*-independent constant $C=C(u_0,v_0, \Gamma , \omega , \delta )>0$. By a similar argument, one obtains

5.14 $$\begin{aligned} \Vert v(t)\Vert _{L^q(\Gamma )} \le Ct^{-\frac{1}{2}\left( 1-\frac{1}{q}\right) }e^{-\delta t}\Vert v_0\Vert _{L^{1}(\Gamma )}+C(1+{ M_0'}). \end{aligned}$$

Then for $0<\varepsilon \ll 1$ there exists $C=C(u_0,v_0, \Gamma , \omega , \delta , \varepsilon )>0$ such that

5.15 $$\begin{aligned} \Vert v(t;u_0,v_0)\Vert _{W^{1,q}(\Gamma )}\le C ({ M_0'}+1), \text { for all } t\in [\varepsilon , \min (T, T_{\max })). \end{aligned}$$

By the second identity in (4.50), for sufficiently small $\varepsilon$ we also have

5.16 $$\begin{aligned} \Vert v(t;u_0,v_0)\Vert _{W^{1,q}(\Gamma )}\le C, \text { for all } t\in [0,\varepsilon ]. \end{aligned}$$

Combining (5.15), (5.16) we obtain (5.6). $\square$

#### Lemma 5.2

Assume hypotheses of Theorem 1.2. Then the following assertions hold for arbitrary $q\ge 1$.

1. If $\tau =0$ then there exists $M(q,T,u_0)>0$ such that
  5.17 $$\begin{aligned} \Vert (u(t;u_0), v(t;u_0))\Vert _{L^q(\Gamma )\times \widehat{W}^{1,q}(\Gamma )}\le M(q,T,u_0),\ t\in [0,\min \{T,T_{\max }(u_0)\}). \end{aligned}$$
  Moreover, if $m>0$ or $k=l=m=0$, then $M(q,T,u_0)$ can be chosen independently of *T*.
2. If $\tau >0$ then there exists $M(q,T,u_0, v_0)>0$ such that
  5.18 $$\begin{aligned} \Vert (u(t;u_0), v(t;u_0))\Vert _{L^q(\Gamma )\times \widehat{W}^{1,q}(\Gamma )}\le M(q,T,u_0, v_0),\ t\in [0,\min \{T,T_{\max }(u_0, v_0)\}). \end{aligned}$$
  Moreover, if $m>0$ or $k=l=m=0$, then $M(q,T,u_0)$ can be chosen independently of *T*.

#### Proof

First, for $\tau \ge 0$ and $n\ge 2$ we claim the following auxiliary identity

5.19 $$\begin{aligned} \frac{1}{n} \frac{d}{dt}\int _{\Gamma } u^{n}dx= -(n-1) \int _{\Gamma } u^{n-2}|u_{x}|^2dx +(n-1)\chi \int _{\Gamma }u^{n-1}u_x v_x dx + \int _{\Gamma }u^{n-1} g(u) dx. \end{aligned}$$

To begin the derivation of (5.19) let us multiply both sides of the first equation in (1.14) by $u^{n -1}$ and integrate over $\Gamma$

5.20 $$\begin{aligned} \int _{\Gamma } u^{n-1} u_tdx = \int _{\Gamma } u^{n-1}u_{xx}dx -\chi \int _{\Gamma }u^{n-1}(u v_x)_x dx + \int _{\Gamma }u^{n-1} g(u) dx. \end{aligned}$$

Then using

5.21 $$\begin{aligned} \int _{\Gamma } u^{n-1} u_t dx = \frac{1}{n} \frac{d}{dt}\int _{\Gamma } u^{n}dx, \end{aligned}$$

we infer

5.22 $$\begin{aligned} \frac{1}{n} \frac{d}{dt}\int _{\Gamma } u^{n}dx= \int _{\Gamma } u^{n-1}u_{xx}dx-\chi \int _{\Gamma }u^{n-1}(u v_x)_xdx + \int _{\Gamma }u^{n-1} g(u) dx. \end{aligned}$$

Next, integrating by parts we obtain

5.23 $$\begin{aligned} \int _{\Gamma } u^{n-1}u_{xx}dx&= -\int _{\Gamma } ( u^{n-1})_xu_{x}dx+\sum _{\theta \in \mathcal {V}}\sum _{e\sim \theta }u^{n-1}_e(\theta )\partial _{\nu }u_e(\theta )\nonumber \\&= -\int _{\Gamma } ( u^{n-1})_xu_{x}dx+\sum _{\theta \in \mathcal {V}}u^{n-1}(\theta )\sum _{e\sim \theta }\partial _{\nu }u_e(\theta )=-\int _{\Gamma } ( u^{n -1})_xu_{x}dx, \end{aligned}$$

where we used the fact that *u* satisfies Neumann–Kirchhoff conditions, that is, $u_e(\vartheta )=u(\vartheta )$ for every edge *e* adjacent to vertex $\vartheta$ and

5.24 $$\begin{aligned} \sum _{e\sim \theta }\partial _{\nu }u_e(\theta )=0. \end{aligned}$$

Integrating by parts the second term in the right-hand side of (5.22) in a similar fashion we arrive at (5.19).

(1) It suffices to prove the assertion for $q=n\in {\mathbb {N}}$. We prove this by induction.

The base case $q=1$ is given by Lemma 5.1. Assume that the it holds for $q=n-1$ ($n\ge 2$). To make the induction step we will derive the following inequality

5.25 $$\begin{aligned} \frac{1}{n} \frac{d}{dt}\int _{\Gamma } u^{n}dx\le C\left( 1+\int _{\Gamma } u^ndx-\left( \int _\Gamma u^{n}dx\right) ^{1+1/n}\right) , t\in (0,\min \{T,T_{\max }\}), \end{aligned}$$

for some $C=C(M(n-1,T,u_0))>0$ (with $x\mapsto C(x)$ having no blow-ups). Assuming (5.25) one obtains the assertions item (1) as follows:

- if $m>0$ or $k=l=m=0$ then, by induction assumption, $M(n-1, T, u_0)$ can be chosen independently from *T* in which case (5.25) yields $M(n,u_0)>0$ such that
  $$\begin{aligned} \int _\Gamma u^n(t;u_0)dx\le M(n,u_0), \end{aligned}$$
- if $m=0$ and $k^2+l^2\not =0$ then (5.25) yields
  $$\begin{aligned} \int _\Gamma u^n(t;u_0)dx\le M(n,T,u_0). \end{aligned}$$

Let us now derive (5.25). To that end let us rewrite the second term in the right-hand side of (5.19). Multiplying both sides of the second equation in (1.14) (recall that $\tau =0$) by $u^{n}$ and integrating by parts as before yields

$$\begin{aligned} n \int _{\Gamma } u^{n-1} u_{x}v_{x} dx= - \int _{\Gamma }u^{n} vdx + \int _{\Gamma }u^{n+1} dx. \end{aligned}$$

Combining this with (5.19) one obtains

5.26 $$\begin{aligned} \frac{1}{n} \frac{d}{dt}\int _{\Gamma } u^{n}dx&= -(n-1) \int _{\Gamma } u^{n-2}|u_{x}|^2dx -\frac{(n-1)\chi }{n} \int _{\Gamma }u^{n}vdx\nonumber \\&\quad + \frac{(n-1)\chi }{n} \int _{\Gamma }u^{n+1}dx+ \int _{\Gamma }u^{n-1} g(u) dx. \end{aligned}$$

This together with (1.17) implies that

5.27 $$\begin{aligned} \frac{1}{n} \frac{d}{dt}\int _{\Gamma } u^{n}dx&\le -(n-1) \int _{\Gamma } u^{n -2}|u_{x}|^2dx +\frac{(n -1)\chi }{n} \int _{\Gamma }u^{n+1}dx\nonumber \\&\quad + k \int _{\Gamma }u^{n-1} dx+l\int _{\Gamma } u^ndx, \end{aligned}$$

here, and throughout the proof, the negative term $\int _{\Gamma }-mu^{n+\varepsilon }dx$ has been dropped. Noticing that

$$\begin{aligned} \int _\Gamma u^{n-2}|u_x|^2dx=\frac{4}{n^2}\int _\Gamma \left| (u^{\frac{n}{2}})_x\right| ^2 dx, \end{aligned}$$

we proceed

5.28 $$\begin{aligned} \frac{1}{n} \frac{d}{dt}\int _{\Gamma } u^{n}dx&\le -\frac{4(n-1)}{n^2} \int _{\Gamma } |(u^{\frac{n}{2}})_{x}|^2dx \nonumber \\&\quad + k \int _{\Gamma }u^{n-1} dx+l\int _{\Gamma } u^ndx+\frac{(n -1)\chi }{n}\int _\Gamma u^{n+1}dx. \end{aligned}$$

Let us observe that

5.29 $$\begin{aligned} \int _\Gamma u^{n+1}dx= \int _\Gamma \big (u^{\frac{n}{2}}\big )^{\frac{2(n+1)}{n}}dx=\Vert u^{\frac{n}{2}}\Vert _{L^{\frac{2(n+1)}{n}}(\Gamma )}^{\frac{2(n+1)}{n}}, \end{aligned}$$

and use the Gagliardo-Nirenberg interpolation inequality to obtain

5.30 $$\begin{aligned} \Vert u^{\frac{n}{2}}\Vert _{L^{\frac{2(n+1)}{n}}(\Gamma )}\le C\Vert (u^{\frac{n}{2}})_x\Vert _{L^2(\Gamma )}^\theta \Vert u^{\frac{n}{2}}\Vert _{L^{\frac{2(n-1)}{n}}(\Gamma )}^{1-\theta }+C\Vert u^{\frac{n}{2}}\Vert _{L^{\frac{2(n-1)}{n}}(\Gamma )}, \end{aligned}$$

where $\theta =\frac{2n}{(n+1)(2n-1)}$ and $C=C(n, \Gamma )>0$. Therefore, one has

5.31 $$\begin{aligned} \int _\Gamma u^{n+1}dx\le C \Big (\int _\Gamma |(u^{\frac{n}{2}})_x|^2dx\Big )^{\frac{2}{2n-1}} \Big (\int _\Gamma u^{n-1}dx\Big )^{\frac{2(2n^2-n-1)}{(2n-1)n}}+C\Big (\int _\Gamma u^{n-1}dx\Big )^{\frac{n+1}{n-1}}. \end{aligned}$$

Applying Young’s inequality to the first term in the right-hand side above, we obtain $C=C(n)>0$ such that

5.32 $$\begin{aligned} \int _\Gamma u^{n+1}dx&\le \frac{2}{n\chi } \int _{\Gamma } |(u^{\frac{n}{2}})_{x}|^2dx + C \Big (\int _\Gamma u^{n-1}dx\Big )^{\frac{2(2n^2-n-1)}{(2n-1)n}\frac{2n-1}{2n-3}}\nonumber \\&\quad +C\Big (\int _\Gamma u^{n-1}dx\Big )^{\frac{n+1}{n-1}}, \end{aligned}$$

This together with (5.28) yield

5.33 $$\begin{aligned} \frac{1}{n} \frac{d}{dt}\int _{\Gamma } u^{n}dx&\le k\int _{\Gamma }u^{n-1} dx+l\int _{\Gamma } u^ndx-\frac{(n -1)\chi }{2n}\int _\Gamma u^{n+1}dx\nonumber \\&\le M(n-1,T, u_0)+l\int _{\Gamma } u^ndx-\frac{(n -1)\chi }{2n}\int _\Gamma u^{n+1}dx. \end{aligned}$$

Then using

5.34 $$\begin{aligned} \int _{\Gamma } u^n dx\le \left( \int _{\Gamma } u^{n+1}dx\right) ^{\frac{n}{n+1}}|\Gamma |^{\frac{1}{n+1}}, \end{aligned}$$

we obtain (5.25).

(2) It suffices to prove (5.18) for $q=n\in {\mathbb {N}}$. We prove this by induction. The base case $q=1$ is given by Lemma 5.1. Assume that it holds for $q=n-1$ ($n\ge 2$). To make the induction step we will derive (5.25) with $C=C( M(n-1, T, u_0, v_0))>0$. Given such an inequality, the assertions of item (2) follow as in the proof of item (1).

Let us now derive (5.25) with $C=C( M(n-1, T, u_0, v_0))>0$ for $\tau =1$ (the derivation for $\tau >0$ is analogous). Combining (1.17) and (5.19) one obtains

$$\begin{aligned} \frac{1}{n}\frac{d}{dt}\int _{\Gamma }u^{n}dx&\le -(n-1)\int _{\Gamma } u^{n-2} |u_x|^2dx+(n-1)\chi \int _{\Gamma }u^{n-1}u_x v_xdx \\&\quad +\int _{\Gamma }u^{n-1}(k+lu)dx\\&\le -\frac{(n-1)}{2}\int _{\Gamma } u^{n-2} |u_x|^2dx+\frac{(n-1)\chi ^2}{2}\int _{\Gamma }u^{n} |v_x|^2dx \\&\quad +\int _{\Gamma }u^{n-1}(k+lu)dx, \end{aligned}$$

where in the second step we used Young’s inequality for

5.35 $$\begin{aligned} \chi u^{n-1} u_x v_x= (u^{\frac{n}{2}-1}u_x )(\chi u^{\frac{n}{2}} v_x). \end{aligned}$$

Next, using Young’s inequality and Lemma  5.1 (2) one obtains

5.36 $$\begin{aligned} \frac{(n-1)\chi ^2}{2}\int _\Gamma u^n|v_x|^2dx&\le \int _\Gamma u^{n+1}dx+C\int _\Gamma |v_x|^{2(n+1)}dx\nonumber \\&\le \int _\Gamma u^{n+1}dx +C\left( 1+N\right) ^{1/(2(n+1))}\nonumber \\&\le \int _\Gamma u^{n+1}dx +C(M(1, T, u_0, v_0))^{1/(2(n+1))}, \end{aligned}$$

for some $C>0$. Therefore, one has

5.37 $$\begin{aligned} \frac{1}{n}\frac{d}{dt}\int _{\Gamma }u^{n}dx&\le -\frac{(n-1)}{2}\int _{\Gamma } u^{n-2} |u_x|^2dx+\int _\Gamma u^{n+1}dx \nonumber \\&\quad + k \int _{\Gamma }u^{n-1}dx +l \int _\Gamma u^ndx\nonumber \\&=-\frac{2(n-1)}{n^2}\int _\Gamma |( u^{\frac{n}{2}})_x|^2dx +\int _\Gamma u^{n+1}dx \nonumber \\&\quad + k \int _{\Gamma }u^{n-1}dx +l \int _\Gamma u^ndx + C(M(1, T, u_0, v_0))^{1/(2(n+1))}, \end{aligned}$$

for some $C>0$. Using (5.31) and Young’s inequality one obtains

5.38 $$\begin{aligned} \int _\Gamma u^{n+1}dx&\le \frac{(n-1)}{n^2} \int _{\Gamma } |(u^{\frac{n}{2}})_{x}|^2dx + C \Big (\int _\Gamma u^{n-1}dx\Big )^{\frac{2(2n^2-n-1)}{(2n-1)n}\frac{2n-1}{2n-3}}\nonumber \\&\quad +C\Big (\int _\Gamma u^{n-1}dx\Big )^{\frac{n+1}{n-1}}. \end{aligned}$$

Then combining (5.37), (5.38), (5.34) and the induction hypothesis we get (5.25) with $C=C( M(n-1, T, u_0, v_0))>0$. $\square$

### Proof of Theorem 1.2

#### Proof of Theorem 1.2

The finite time blow-up of $t\mapsto \Vert (u(s), v(s))\Vert _{L^{q}(\Gamma )\times W^{1,q}(\Gamma )}$ has been ruled out in Lemma 5.2, that is, $T_{\max }=\infty$.

To prove (1.18) and (1.20) it suffices to obtain a uniform, with respect to time, bound on $L^{\infty }(\Gamma )$-norm of each term in the right-hand side of (5.3), where we assume without loss of generality that $\sigma >\sigma _0$ for $\sigma _0$ is as in Corollary 2.1. This choice, in particular, allows us to use (B.36) with $t\in (0, T]$ replaced by $t\in (0,\infty )$, some $\delta \in (0, \sigma )$ and *t*-independent constant $C>0$.

Let us now switch to the first term in (5.3). Using comparison principle from Theorem C.2 with $f(t,x,u,v)=u$ one obtains

5.39 $$\begin{aligned} \Vert e^{t(\Delta -\sigma )}u_0\Vert _{L^{\infty }(\Gamma )}\le \max _{x\in \overline{\Gamma }}|u_0(x)|, t>0. \end{aligned}$$

Let us estimate $L^{\infty }-$norm of the second term in (5.3). Let us pick $q>2$, $\beta \in (0,1/8)$ with $2\beta -q^{-1}>0$ and recall that $u(t)\in \mathcal {X}^{\beta }_{q}$, $t\in (0, T_{\max })$. Using the embedding $\mathcal {X}^{\beta }_{q}\hookrightarrow L^{\infty }(\Gamma )$ we obtain

5.40 $$\begin{aligned}&\int _0^t \left\| e^{(\Delta -\sigma )(t-s)}\partial _x \big ( u(s)\partial _x v(s)\big )\right\| _{\mathcal {X}_q^{\beta }} ds\nonumber \\&\quad \le C\int _0^t (t-s)^{-\beta -\frac{q+1}{2q} }e^{{-\delta (t-s)}}\Vert (u(s), v(s))\Vert _{L^{q}(\Gamma )\times W^{1,q}(\Gamma )}ds\nonumber \\&\quad \le C \sup \limits _{s\in [0, \infty )}\Vert (u(s), v(s))\Vert _{L^{q}(\Gamma )\times W^{1,q}(\Gamma )}<\infty , t>0, \end{aligned}$$

where *C* is *t*-independent and the last inequality holds due to the forgoing assumption that either $m>0$ or $k=l=m=0$ and Lemma 5.2.

Next, employing (1.17), Lemma 5.2 and the comparison principle we obtain

5.41 $$\begin{aligned} \int _{0}^{t} e^{(t-s)(\Delta -\sigma )}(g(u(s))+\sigma u(s))ds&\le \int _{0}^{t} e^{(t-s)(\Delta -\sigma )}(C+l u(s))ds\nonumber \\&\le C(u_0)\int _{0}^{t} e^{-(t-s)\sigma /2}ds, t>0 \end{aligned}$$

Combining (5.3), (5.39), (5.40), (5.41), one obtains

5.42 $$\begin{aligned} \limsup _{t\rightarrow \infty } \Vert u(t)\Vert _{L^\infty (\Gamma )}<\infty . \end{aligned}$$

$$\square $$

## Functional spaces, interpolation spaces, Sobolev embedding theorems on metric graphs

In this section, we record several facts about fractional power spaces $\mathcal {X}^{\alpha }_p$, $\alpha \in (0,1)$, $1\le p< \infty$ generated by the Neumann–Kirchhoff Laplacian on compact metric graphs, more concretely, by the analytic semigroup in $e^{t\Delta }$. By definition, see, e.g., [26, Section 1.3]., $\mathcal {X}^{\alpha }_p{:}{=}\operatorname {dom}(({ \sigma }I_{L^p(\Gamma )}-\Delta )^{\alpha })$ is equipped with the graph norm of $({\sigma }I_{L^p(\Gamma )}-\Delta )^{\alpha }$ for some $\sigma >0$ (we note that $\operatorname {dom}(({ \sigma }I_{L^p(\Gamma )}-\Delta )^{\alpha })=\operatorname {dom}(({{\tilde{\sigma }}}I_{L^p(\Gamma )}-\Delta )^{\alpha })$ and the graph norms of $\operatorname {dom}(({ \sigma }I_{L^p(\Gamma )}-\Delta )^{\alpha })=\operatorname {dom}(({{\tilde{\sigma }}}I_{L^p(\Gamma )}-\Delta )^{\alpha })$ are equivalent for $\sigma ,{\tilde{\sigma }}>0$). Throughout this paper we use compact embeddings of $\mathcal {X}^{\alpha }_p$ into various function spaces. Such embeddings are well known in the case of classical domains $\Omega \subset {\mathbb {R}}^n$, cf. [26, Chapter 1]. To the best of our knowledge, these type of embedding for metric graphs, although expected, have not appeared in print. For completeness of exposition we present them in Theorem A.1. Let us first introduce some notation. The edge-wise direct sum of Banach spaces of functions will be denoted by , in particular, we write

A.1 $$\begin{aligned} \widehat{C}_0^{\infty }(\Gamma ){:}{=} \bigoplus _{e\in \mathcal {E}} C_0^{\infty }(e), \,\,\,&\widehat{W}^{k,p}({\Gamma }){:}{=} \bigoplus _{e\in \mathcal {E}}W^{k,p}(e), \,\, k\in {\mathbb {N}}_0,\,\,\, \widehat{C}^{\nu }(\overline{{\Gamma }}) {:}{=} \bigoplus _{e\in \mathcal {E}}\widehat{C}^{\nu }(\overline{e}), \end{aligned}$$

where $\nu >0$ and $C^{\nu }(\overline{e})$ denotes the usual space of Hölder continuous functions defined on the closed interval $\overline{e}$ endowed with the norm

A.2 $$\begin{aligned} \Vert u\Vert _{C^\nu ({\bar{e}})}=\sum _{\alpha \in {\mathbb {N}}_0,\alpha \le [\nu ]}\sup _{x\in {\bar{e}}} |u^{(\alpha )}(x)|+\sup _{x,y\in {\bar{e}},x\not =y} \frac{|u^{([\nu ])}(x)-u^{([\nu ])}(y)|}{|x-y|^{\nu -[\nu ]}}. \end{aligned}$$

Let us note that the edge-wise direct sums introduced in (A.1) induce no vertex conditions as oppose to the space of continuous functions on the closure $\overline{{\Gamma }}$ of the graph

A.3 $$\begin{aligned} C({\overline{\Gamma }})=\{u\in \widehat{C}({\overline{\Gamma }})\, :\, u\,\, \mathrm{is \,\, continuous\,\, at\,\, the\,\, vertices\,\, of}\,\, \Gamma \}. \end{aligned}$$

In the following theorem $(L^p(\Gamma ),\widehat{W}^{2,p}(\Gamma ))_{\theta ,q}$ denotes the interpolation space between $L^p(\Gamma )$ and $\widehat{W}^{2,p}(\Gamma )$ via the *K*-method, where $0<\theta <1$ and $1\le q<\infty$ (see  [58, Section 1.3.2] for definition).

### Theorem A.1

Suppose that $1\le p<\infty$. Then one has

1. For any $q\ge p$ and $s-\frac{1}{p}> t-\frac{1}{q}$, one has
  A.4
  A.5
  and for $s\in (0,1)\setminus \{\frac{1}{2}\}$ one has
  A.6 $$\begin{aligned} (L^p(\Gamma ), \widehat{W}^{2,p}(\Gamma ))_{s,p}=\widehat{W}^{2s,p}(\Gamma ), \end{aligned}$$
  where  denotes compact embedding.
2. One has
  A.7
  A.8
  and
  A.9

### Proof

(1) (A.4) and (A.5) follow from [1, Theorem 11.5], and (A.6) follows from [1, Theorem 11.6]. (2) First, (A.7) follows from  [58, Section 1.15.2, (unlabled) Theorem on page 101].

To prove (A.8), for a given $0<\nu <2\alpha -\frac{1}{p}$, let us choose $\theta \in (0,1)$ such that $\frac{\alpha }{2}\theta \not =1$ and $2 \alpha \theta -\frac{1}{p}>\nu$. Then one has

$$\begin{aligned} \mathcal {X}_p^\alpha \subset (L^p(\Gamma ), \mathcal {X}_p^\alpha )_{\theta ,p}=(L^p(\Gamma ),\mathcal {D}(\mathcal {A}_p))_{{\alpha }\theta ,p}\underset{(\hbox {A.7})}{\subset } ((L^p(\Gamma ),\widehat{W}^{2,p}(\Gamma ))_{{\alpha \theta },p}\underset{ (\hbox {A.6})}{=}\widehat{W}^{2\alpha \theta ,p}(\Gamma ). \end{aligned}$$

The embedding (A.9) follows from (A.5) and (A.8). $\square$

### Proposition A.1

[25, Theorem 1.4.3] Let $\sigma >0$, $\alpha \in (0,1)$, $p\in [1, \infty )$ and $\delta \in (0, \sigma )$. Then there exists $C>0$ such that for arbitrary $t>0$ and $u\in L^p(\Gamma )$, $v\in \mathcal {X}^{\alpha }$ one has

A.10 $$\begin{aligned}&\Vert (\sigma -\Delta )^{\alpha }e^{(\Delta -\sigma )t}u\Vert _{L^p(\Gamma )}\le C e^{-\delta t}t^{-\alpha } \Vert u\Vert _{L^p(\Gamma )}, \end{aligned}$$

A.11 $$\begin{aligned}&\Vert (e^{(\Delta -\sigma )t}-I)v\Vert _{L^p(\Gamma )}\le C t^{\alpha } \Vert v\Vert _{\mathcal {X}^{\alpha }}. \end{aligned}$$

## The $L^p-L^q$ properties of auxiliary nonlinear mappings

In this section, we establish $L^p-L^q$ properties of nonlinear mappings in (4.7), (4.8), (4.9).

### Hypothesis B.1

Let $F: {\mathbb {R}}^2\rightarrow {\mathbb {R}}$, $p\ge 1$, $R>0$. Assume that $F\in C^{1,1}({\mathbb {R}}^2)$ and that the following polynomial growth bounds hold

B.1 $$\begin{aligned} { \sum _{|\alpha |\le 1}}|D^{\alpha } F(s,t)| \le C(1+|s|^{\mu }+|t|^{\nu }),\ (s,t)\in {\mathbb {R}}^2, \end{aligned}$$

for some $C>0$, $\mu , \nu \ge 0$ with $\gamma {:}{=}\mu +\nu \ge 1$. Assume that $p\ge 2 \gamma$.

### Proposition B.1

Assume Hypothesis B.1. For $U=(u_1, u_2)\in (L^p(\Gamma ))^2$ and let us define

B.2 $$\begin{aligned} \mathcal {F}(U)(x){:}{=}F(u_1(x), u_2(x)),\ x\in \Gamma . \end{aligned}$$

Then the following assertions hold.

1. $$\mathcal {F}$$ maps $$L^p(\Gamma )$$ to $$L^{{p}/{\gamma }}(\Gamma )$$.
2. There exists $C>0$ such that for $\Vert U\Vert _{(L^p(\Gamma ))^2}\le R$ one has
  B.3 $$\begin{aligned} \Vert \mathcal {F}(U)\Vert _{L^{{p}/{\gamma }}(\Gamma )}\le C\left( 1+\Vert U\Vert _{(L^p(\Gamma ))^2}\right) , \end{aligned}$$
  that is $\mathcal {F}:L^p(\Gamma )\rightarrow L^{{p}/{\gamma }}(\Gamma )$ is a bounded (but in general nonlinear) mapping.
3. There exists $C>0$ such that for $\Vert U\Vert _{(L^p(\Gamma ))^2}\le R$, $\Vert V\Vert _{(L^p(\Gamma ))^2}\le R$ one has
  B.4 $$\begin{aligned} \Vert \mathcal {F}(U)-\mathcal {F}(V)\Vert _{L^{{p}/{\gamma }}(\Gamma )}\le C\Vert U-V\Vert _{(L^p(\Gamma ))^2}, \end{aligned}$$
  that is, $\mathcal {F}:L^p(\Gamma )\rightarrow L^{{p}/{\gamma }}(\Gamma )$ is locally Lipschitz continuous.

### Proof

(1) &(2). Using (B.1) one infers

B.5 $$\begin{aligned} \int _{\Gamma }\left| \mathcal {F}(U)\right| ^{\frac{p}{\gamma }}dx&\le 3^{\frac{p}{\gamma }}C(F)\int _{\Gamma }\big (1+|u_1|^{\frac{\mu p}{\gamma }}+|u_1|^{\frac{\nu -p}{\gamma }}\big )dx\nonumber \\&\le C(F, \Gamma ,p)(1+\Vert u_1\Vert _{L^p(\Gamma )}^p+\Vert u_2\Vert _{L^p(\Gamma )}^p). \end{aligned}$$

(3) One has

B.6 $$\begin{aligned} \int _{\Gamma }\left| \mathcal {F}(U)-\mathcal {F}(V)\right| ^{\frac{p}{\gamma }}dx&\le \ 2^{{\frac{p}{\gamma }}}\int _{\Gamma }\left| F(u_1,u_2)-F(v_1,u_2)\right| ^{\frac{p}{\gamma }}dx\nonumber \\&\quad +2^{{\frac{p}{\gamma }}}\int _{\Gamma }\left| F(v_1,u_2)-F(v_1,v_2)\right| ^{\frac{p}{\gamma }}dx. \end{aligned}$$

Let us estimate the second term and note that the first term can be estimated analogously. We have

B.7 $$\begin{aligned}&\int _{\Gamma }\left| F(v_1(x),u_2(x))-F(v_1(x),v_2(x))\right| ^{\frac{p}{\gamma }}dx=\int _{\Gamma }\left| \int _{v_2(x)}^{u_2(x)}\partial _2F(v_1(x),\xi )d\xi \right| ^{\frac{p}{\gamma }} dx\nonumber \\&\quad =\int _{\Gamma }|u_2(x)-v_2(x))|^{\frac{p}{\gamma }}\left| \int _0^1\partial _2F(v_1(x),v_2(x)-\rho (v_2(x)-u_2(x)))d\rho \right| ^{\frac{p}{\gamma }} dx. \end{aligned}$$

Using Hölder’s inequality with exponents $\gamma$, $\gamma (\gamma -1)^{-1}$ we obtain

B.8 $$\begin{aligned}&\left\| F(v_1,u_2)-F(v_1,v_2)\right\| _{L^{{p}/{\gamma }}(\Gamma )}^{\frac{p}{\gamma }}dx\le \int _{\Gamma }\left| \int _{v_2(x)}^{u_2(x)}\partial _2F(v_1(x),\xi )d\xi \right| ^{\frac{p}{\gamma }} dx\nonumber \\&\quad =\Vert u_2(x)-v_2(x)\Vert _{L^p(\Gamma )}^{\frac{p}{\gamma }}\left\| \left| \int _0^1\partial _2F(v_1(x),v_2(x)-\rho (v_2(x)-u_2(x)))d\rho \right| ^{\frac{p}{\gamma }} \right\| _{L^{\frac{\gamma }{\gamma -1}}(\Gamma )}\ \end{aligned}$$

Let us estimate the second term in the above product

B.9 $$\begin{aligned}&\left\| \left| \int _0^1 \partial _2F(v_1,v_2-\rho (v_2-u_2))d\rho \right| ^{p/\gamma }\right\| _{L^{\frac{\gamma }{\gamma -1}}(\Gamma )}=\left\| \int _0^1 \partial _2F(v_1,v_2-\rho (v_2-u_2))d\rho \right\| _{L^{\frac{p}{\gamma -1}}(\Gamma )}^{p/\gamma }\nonumber \\&\quad \le \left( \int _0^1 \Vert \partial _2F(v_1,v_2-\rho (v_2-u_2))\Vert _{L^{\frac{p}{\gamma -1}}(\Gamma )} d\rho \right) ^{p/\gamma }\nonumber \\&\quad \le C(F, \Gamma , p)\left( \int _0^1\left( \int _{\Gamma } 1+|v_1|^{\frac{\widehat{\mu } p}{\gamma -1}} +|v_2-\rho (v_2-u_2)|^{\frac{\widehat{\nu } p}{\gamma -1}} dx\right) ^{\frac{\gamma -1}{p}} d\rho \right) ^{p/\gamma }\nonumber \\&\quad \le C(F, \Gamma , p)\left( \int _0^1 \left( |\Gamma |+ \Vert v_1 \Vert ^p_{L^p(\Gamma )}+ \Vert v_2-\rho (v_2-u_2) \Vert ^p_{L^p(\Gamma )}\right) ^{\frac{\gamma -1}{p}}d\rho \right) ^{p/\gamma }\nonumber \\&\quad \le C(F, \Gamma , p, R), \end{aligned}$$

Combining (B.6), (B.7), (B.8), (B.9) we arrive at (B.4). $\square$

### Proposition B.2

Assume Hypothesis B.1, let $T\in (0, \infty )$, $q \ge r\ge 2\gamma$, $\sigma >0$, $\delta \in (0, \sigma )$, fix $\phi \in L^q(\Gamma )$, $\psi \in L^r(\Gamma )$ and $\rho >\max \{\Vert \phi \Vert _{L^q(\Gamma )}, \Vert \psi \Vert _{L^r(\Gamma )}\}$. For $u\in L^q(\Gamma )$ define a mapping

B.10 $$\begin{aligned} \mathcal {K}(u)(x){:}{=}F\left( u(x), \phi (x)\right) \psi (x), x\in \Gamma . \end{aligned}$$

Then $\mathcal {K}$ maps $L^q(\Gamma )$ into $L^{\frac{r}{2\gamma }}(\Gamma )$, $e^{(\Delta -\sigma I)t}\mathcal {K}$ maps $L^q(\Gamma )$ into $L^q(\Gamma )$ and both mappings are bounded and locally Lipschitz continuous. That is, for $R>0$ there exists a constant $C>0$, which in general depends on *T*, such that for ${ u}, v\in L^p(\Gamma )$ with $\Vert u\Vert _{L^q(\Gamma )}\le R$, $\Vert v\Vert _{L^q(\Gamma )}\le R$ one has

B.11 $$\begin{aligned} \Vert \mathcal {K}(u)\Vert _{{L^{\frac{r}{2\gamma }}(\Gamma )}}&\le C{ (1+\Vert u\Vert _{L^q(\Gamma )})}, \end{aligned}$$

B.12 $$\begin{aligned} \Vert \mathcal {K}(u)-\mathcal {K}(v)\Vert _{{L^{\frac{r}{2\gamma }}(\Gamma )}}&\le C\Vert u-v\Vert _{L^q(\Gamma )},\end{aligned}$$

B.13 $$\begin{aligned} \Vert e^{(\Delta -\sigma I)t}\mathcal {K}(u)-e^{(\Delta -\sigma I)t}\mathcal {K}(v)\Vert _{L^q(\Gamma )}&\le Ce^{-\delta t}t^{-\frac{1}{2}\left( \frac{2\gamma }{r}-\frac{1}{q}\right) }\Vert u-v\Vert _{{L^q(\Gamma )}}, t\in (0,T]. \end{aligned}$$

Furthermore, the mapping $e^{(\Delta -\sigma I)t}\partial _x$, originally defined on $C_0^{\infty }(\Gamma )$, can be extended to a bounded linear operator in $\mathcal {B}( L^{\frac{r}{2\gamma }}(\Gamma ), L^q(\Gamma ))$ so that $e^{(\Delta -\sigma I)t}\partial _{x}\mathcal {K}$ maps $L^q(\Gamma )$ into $L^q(\Gamma )$ and, for *u*, *v* as above, one has

B.14 $$\begin{aligned} \Vert e^{(\Delta -\sigma I)t}\partial _x\mathcal {K}(u)-e^{(\Delta -\sigma I)t}\partial _x\mathcal {K}(v)\Vert _{L^q(\Gamma )}\le Ce^{-\delta t}t^{-\frac{1}{2}\left( \frac{2\gamma }{r}-\frac{1}{q}+1\right) }\Vert u-v\Vert _{{L^q(\Gamma )}}, t\in (0,T]. \end{aligned}$$

Furthermore, if $\sigma >\sigma _0$, where $\sigma _0>0$ is as in Corollary 2.1, then there exists a *T*-independent constant $C>0$ and $0<\delta \ll \sigma$ such that (B.13), (B.14) hold with $t\in (0,T]$ replaced by $t\in (0,\infty )$.

### Proof

To show (B.12) (hence, (B.11)) we note

B.15 $$\begin{aligned}&\Vert \mathcal {K}(u)- \mathcal {K}(v)\Vert _{{L^{\frac{r}{2\gamma }}}(\Gamma )}\le \Vert \big (F(u, \phi )-F(v, \phi )\big )\psi \Vert _{{L^{\frac{r}{2\gamma }}(\Gamma )}}\nonumber \\&\quad \le \Vert F(u, \phi )-F(v, \phi )\Vert _{{L^{\frac{r}{\gamma }}}(\Gamma )}\Vert \psi \Vert _{{L^{\frac{r}{\gamma }}}(\Gamma )}\le C \Vert u-v\Vert _{L^q(\Gamma )}, \end{aligned}$$

where we used Proposition B.1 item (3). Then (B.13) and (B.14) follow from (B.12) combined with (1.25) and (1.21) correspondingly. The last statement follows from Corollary 2.1. $\square$

### Proposition B.3

Assume the setting of Proposition B.1, fix $T\in (0,\infty )$, $\sigma >0$ and $\delta \in (0, \sigma )$.

1. $e^{(\Delta -\sigma I)t}\mathcal {F}(U)$ maps $(L^p(\Gamma ))^2$ into $\widehat{W}^{1, p}(\Gamma )$ boundedly, in fact, this mapping is locally Lipschitz continuous. That is, for $R>0$ there exists $C>0$, which in general depends on *T*, such that for $\Vert U\Vert _{(L^p(\Gamma ))^2}\le R$, $\Vert V\Vert _{(L^p(\Gamma ))^2}\le R$, one has
  B.16 $$\begin{aligned}&\Vert e^{(\Delta -\sigma I)t}\mathcal {F}(U)\Vert _{\widehat{W}^{1, p}(\Gamma )}\le Ce^{-\delta t}\max \{ t^{-\frac{\gamma -1}{2p}}, t^{-\frac{p+\gamma -1}{2p}}\}\left( 1+\Vert U\Vert _{(L^p(\Gamma ))^2}\right) , t\in (0,T], \end{aligned}$$
  B.17 $$\begin{aligned}&\Vert e^{(\Delta -\sigma I)t}\mathcal {F}(U)-e^{(\Delta -\sigma I)t}\mathcal {F}(V)\Vert _{\widehat{W}^{1, p}(\Gamma )}\nonumber \\&\quad \le Ce^{-\delta t}\max ( t^{-\frac{\gamma -1}{2p}}, t^{-\frac{p+\gamma -1}{2p}})\Vert U-V\Vert _{(L^p(\Gamma ))^2}, t\in (0,T]. \end{aligned}$$
2. For $\beta >0$, $h> 0$ one has
  B.18 $$\begin{aligned}&\Vert e^{(\Delta -\sigma I)t}\mathcal {F}(U)\Vert _{\mathcal {X}^{\beta }_p}\le Ce^{-\delta t} t^{-\beta -\frac{\gamma -1}{2p}} \left( 1+\Vert U\Vert _{(L^p(\Gamma ))^2}\right) , t\in (0,T], \end{aligned}$$
  B.19 $$\begin{aligned}&\Vert (e^{(\Delta -\sigma I)h}-I)e^{(\Delta -\sigma I)t}\mathcal {F}(U)\Vert _{\mathcal {X}^{\beta }_p}\le Ch^{\beta }e^{-\delta t} t^{-2\beta -\frac{\gamma -1}{2p}} \left( 1+\Vert U\Vert _{(L^p(\Gamma ))^2}\right) , t\in (0,T]. \end{aligned}$$
3. The mapping $e^{(\Delta -\sigma I)t}\partial _x$ originally defined on $C_0^{\infty }(\Gamma )$ can be extended to a bounded linear operator in $\mathcal {B}(L^{{p}/{\gamma }}(\Gamma ), L^p(\Gamma ))$ so that $e^{(\Delta -\sigma I)t}\partial _{x}\mathcal {F}(U)$ maps $(L^p(\Gamma ))^2$ into $L^p(\Gamma )$ boundedly, this mapping is locally Lipschitz continuous, that is, for $R\ge 0$ there exists $C>0$, which in general depends on T such that for $\Vert U\Vert _{(L^p(\Gamma ))^2}\le R$, $\Vert V\Vert _{(L^p(\Gamma ))^2}\le R$, one has
  B.20 $$\begin{aligned}&\Vert e^{(\Delta -\sigma I)t}\partial _x\mathcal {F}(U)\Vert _{L^p(\Gamma )}\le Ce^{-\delta t}t^{-\frac{p+\gamma -1}{2p}}\left( 1+\Vert U\Vert _{(L^p(\Gamma ))^2}\right) , t\in (0,T], \end{aligned}$$
  B.21 $$\begin{aligned}&\Vert e^{(\Delta -\sigma I)t}\partial _x\mathcal {F}(U)-e^{(\Delta -\sigma I)t}\partial _x\mathcal {F}(V)\Vert _{L^p(\Gamma )}\le Ce^{-\delta t}t^{-\frac{p+\gamma -1}{2p}}\Vert U-V\Vert _{(L^p(\Gamma ))^2}, t\in (0,T]. \end{aligned}$$

Furthermore, if $\sigma >\sigma _0$, where $\sigma _0>0$ is as in Corollary 2.1, then there exists a *T*-independent constant $C>0$ and $0<\delta \ll \sigma$ such that (B.16)–(B.21) hold with $t\in (0,T]$ replaced by $t\in (0,\infty )$.

### Proof

(1) The inequalities (B.16), (B.17) follow from Theorem 1.3 combined with (B.3), (B.4) correspondingly.

(2) The inequality (B.18) follows (B.3) and (A.10). To prove (B.19) we write

B.22 $$\begin{aligned} \Vert (e^{(\Delta -\sigma I)h}-I)e^{(\Delta -\sigma I)t}\mathcal {F}(U)\Vert _{\mathcal {X}^{\beta }_p}&\le Ch^{\beta }e^{-\delta t} \Vert e^{(\Delta -\sigma I)t}\mathcal {F}(U)\Vert _{\mathcal {X}^{2\beta }_p}\nonumber \\&\le Ch^{\beta }e^{-\delta t} t^{-2\beta -\frac{\gamma -1}{2p}} \left( 1+\Vert U\Vert _{(L^p(\Gamma ))^2}\right) . \end{aligned}$$

(3) The inequalities (B.20), (B.21) follow from (1.23) combined with (B.3), (B.4) correspondingly.

The last statement follows from Corollary 2.1. $\square$

### Proposition B.4

Let $\sigma >0$, $p\ge 1$ then there exists $C>0$ such that

B.23 $$\begin{aligned}&\Vert (\Delta -\sigma )^{-1}u\Vert _{L^{\infty }(\Gamma )}\le C \Vert u\Vert _{L^p(\Gamma )}, \end{aligned}$$

B.24 $$\begin{aligned}&\Vert \partial _x(\Delta -\sigma )^{-1}u\Vert _{L^{\infty }(\Gamma )}\le C\Vert u\Vert _{L^p(\Gamma )}, \end{aligned}$$

B.25 $$\begin{aligned}&\Vert (\Delta -\sigma )^{-1}u\Vert _{\widehat{W}^{2,p}(\Gamma )}\le C\Vert u\Vert _{L^p(\Gamma )}, \end{aligned}$$

for all $$t>0$$.

### Proof

Let us recall, e.g., from [10, Corollary 7 in Section 4.2], the following inequality

B.26 $$\begin{aligned} \Vert f\Vert _{L^{\infty }(\Gamma )}\le C\left( \Vert f\Vert _{L^p(\Gamma )}+\Vert \Delta f\Vert _{L^p(\Gamma )}\right) , f\in \widehat{W}^{2,p}(\Gamma ), C>0. \end{aligned}$$

To prove (B.23) we denote $f{:}{=}(\Delta -\sigma )^{-1}u$, that is, $\Delta f-\sigma f=u$, $u\in L^p(\Gamma )$, and employ (B.26) to obtain

B.27 $$\begin{aligned} \Vert f\Vert _{L^{\infty }(\Gamma )}&\le C\left( \Vert f\Vert _{L^p(\Gamma )}+\Vert \Delta f\Vert _{L^p(\Gamma )}\right) = C\left( (1+|\sigma |)\Vert f\Vert _{L^p(\Gamma )}+\Vert u\Vert _{L^p(\Gamma )}\right) \nonumber \\&\le C\left( (1+|\sigma |)\Vert (\Delta -\sigma )^{-1}\Vert _{\mathcal {B}(L^p(\Gamma ))}\Vert u\Vert _{L^p(\Gamma )}+\Vert u\Vert _{L^p(\Gamma )}\right) , \end{aligned}$$

where we implicitly used $p-$independence of the spectrum and resolvent of $\mathcal {A}_p$ obtained in [3, Theorem 3, Section 4.2]. The third inequality follows from (B.24) and

B.28 $$\begin{aligned} \Vert \Delta (\Delta -\sigma )^{-1}u\Vert _{L^p(\Gamma )}\le C\Vert u\Vert _{L^p(\Gamma )}. \end{aligned}$$

$$\square $$

### Corollary B.1

Assume Hypothesis 1.1 and suppose that $\tau =0$. Define

B.29 $$\begin{aligned} \mathcal {F}_3(u){:}{=}(\Delta -\sigma )^{-1}f(u)\text { for } u\in L^p(\Gamma ). \end{aligned}$$

Then $\mathcal {F}_3$ is a bounded mapping between $L^p(\Gamma )$ and $\widehat{W}^{1, p}(\Gamma )$, that is, there exists a constant $C>0$ such that if $\Vert u\Vert _{L^p(\Gamma )}\le R$ then one has

B.30 $$\begin{aligned} \Vert \mathcal {F}_3(u)\Vert _{\widehat{W}^{1, p}(\Gamma )}\le C(1+\Vert u\Vert _{L^p(\Gamma )}). \end{aligned}$$

Moreover, $\mathcal {F}_3$ is locally Lipschitz continuous, that is, for $\Vert u\Vert _{L^p(\Gamma )}\le R$, $\Vert v\Vert _{L^p(\Gamma )}\le R$ one has

B.31 $$\begin{aligned} \Vert \mathcal {F}_3(u)-\mathcal {F}_3(v)\Vert _{\widehat{W}^{1, p}(\Gamma )}\le C\Vert u-v\Vert _{L^p(\Gamma )}. \end{aligned}$$

### Proof

By Proposition B.1 with $F(u,v)=f(u)$, the mapping $u\mapsto f(u)$ from $L^p(\Gamma )$ to $L^{\frac{p}{\gamma }}(\Gamma )$ is bounded and locally Lipschitz. Next, by Proposition B.4, $(\Delta -\sigma )^{-1}$ maps $L^{\frac{p}{\gamma }}(\Gamma )$ into $\widehat{W}^{1,\infty }(\Gamma )$ which is boundedly embedded into $\widehat{W}^{1, p}(\Gamma )$. The assertions follow from the fact that $\mathcal {F}_3$ is a composition of these mappings. $\square$

### Proposition B.5

Assume Hypothesis B.1 and let $T\in (0,\infty )$, $\sigma > 0$, $\delta \in (0, \sigma )$. For $U=(u_1, u_2)\in L^p(\Gamma )\times \widehat{W}^{1, p}(\Gamma )$ let

B.32 $$\begin{aligned} \mathcal {H}(U)(x){:}{=}F\left( u_1(x), u_2(x)\right) \partial _x u_2(x), x\in \Gamma . \end{aligned}$$

Then $\mathcal {H}$ maps $L^p(\Gamma )\times \widehat{W}^{1, p}(\Gamma )$ into $L^{\frac{p}{2\gamma }}(\Gamma )$, $e^{(\Delta -\sigma I)t}\mathcal {H}$ maps $L^p(\Gamma )\times \widehat{W}^{1, p}(\Gamma )$ into $L^p(\Gamma )$ and both mappings are bounded and locally Lipschitz continuous. That is, for $R>0$ there exists $C>0$, which in general depends on *T*, such that for $U, V\in L^p(\Gamma )\times \widehat{W}^{1, p}(\Gamma )$ with $\Vert U\Vert _{L^p(\Gamma )\times \widehat{W}^{1, p}(\Gamma )}\le R$, $\Vert V\Vert _{L^p(\Gamma )\times \widehat{W}^{1, p}(\Gamma )}\le R$ one has

B.33 $$\begin{aligned} \Vert \mathcal {H}(U)-\mathcal {H}(V)\Vert _{{L^{\frac{p}{2\gamma }}(\Gamma )}}\le C\Vert U-V\Vert _{L^p(\Gamma )\times \widehat{W}^{1, p}(\Gamma )}. \end{aligned}$$

In addition, one has

B.34

Furthermore, the mapping $e^{(\Delta -\sigma I)t}\partial _x$, originally defined on $C_0^{\infty }(\Gamma )$, can be extended to a bounded linear operator in $\mathcal {B}(L^{\frac{p}{2\gamma }}(\Gamma ), L^p(\Gamma ))$ so that $e^{(\Delta -\sigma I)t}\partial _{x}\mathcal {H}$ maps $L^p(\Gamma )\times \widehat{W}^{1, p}(\Gamma )$ into $L^p(\Gamma )$ and, for *U*, *V* as above, one has

B.35 $$\begin{aligned} \Vert e^{(\Delta -\sigma I)t}\partial _x\mathcal {H}(U)-e^{(\Delta -\sigma I)t}\partial _x\mathcal {H}(V)\Vert _{L^p(\Gamma )}\le Ce^{-\delta t}t^{-\frac{p+2\gamma -1}{2p}}\Vert U-V\Vert _{{L^p(\Gamma )\times \widehat{W}^{1, p}(\Gamma )}}, t\in (0,T]. \end{aligned}$$

In addition, for $\beta \ge 0$, $h>0$ one has $e^{(\Delta -\sigma I)t}\partial _x \mathcal {H}(U)\in \mathcal {X}^{\beta }_p$ and the following inequalities hold

B.36 $$\begin{aligned}&\Vert e^{(\Delta -\sigma I)t}\partial _x \mathcal {H}(U)\Vert _{\mathcal {X}^{\beta }_p}\le Ce^{-\delta t} t^{-\beta -\frac{p+2\gamma -1}{2p}}\Vert U\Vert _{{L^p(\Gamma )\times \widehat{W}^{1, p}(\Gamma )}}, t\in (0,T], \end{aligned}$$

B.37 $$\begin{aligned}&\Vert (e^{(\Delta -\sigma I)h}-I)e^{(\Delta -\sigma I)t}\partial _x \mathcal {H}(U)\Vert _{\mathcal {X}^{\beta }_p}\le Ch^{\beta }e^{-\delta t} t^{-2\beta -\frac{p+2\gamma -1}{2p}}\Vert U\Vert _{{L^p(\Gamma )\times \widehat{W}^{1, p}(\Gamma )}}, t\in (0,T]. \end{aligned}$$

Furthermore, if $\sigma >\sigma _0$, where $\sigma _0>0$ is as in Corollary 2.1, then there exists a *T*-independent constant $C>0$ and $0<\delta \ll \sigma$ such that (B.34)–(B.37) hold with $t\in (0,T]$ replaced by $t\in (0,\infty )$.

### Proof

For $\mathcal {F}$ as in (B.2) one has

B.38 $$\begin{aligned} \Vert \mathcal {H}(U)- \mathcal {H}(V)\Vert _{{L^{\frac{p}{2\gamma }}}(\Gamma )}\le \Vert \mathcal {F}\big (U\big )(\partial _xu_2-\partial _xv_2)\Vert _{L^{\frac{p}{2\gamma }}(\Gamma )}+ \Vert \big (\mathcal {F}(U)-\mathcal {F}(V)\big )\partial _xv_2\Vert _{{L^{\frac{p}{2\gamma }}(\Gamma )}}. \end{aligned}$$

Using the Cauchy–Schwartz inequality we obtain

B.39 $$\begin{aligned} \Vert \big (\mathcal {F}(U)-\mathcal {F}(V)\big )\partial _xv_2\Vert _{{L^{\frac{p}{2\gamma }}}(\Gamma )}^{\frac{p}{2\gamma }}&\le \Vert \big (\mathcal {F}(U)-\mathcal {F}(V)\big )\Vert _{L^{{p}/{\gamma }}(\Gamma )}^{\frac{p}{2\gamma }}\Vert \partial _xv_2\Vert ^{\frac{p}{2\gamma }}_{L^{{p}/{\gamma }}(\Gamma )}\nonumber \\&\le C\Vert U-V\Vert _{(L^p(\Gamma ))^2}^{\frac{p}{2\gamma }}\Vert v_2\Vert ^{\frac{p}{2\gamma }}_{\widehat{W}^{1, p}(\Gamma )}, \end{aligned}$$

where we used Proposition B.1 item (3) and $p/\gamma \le p$. By a similar argument one obtains

B.40 $$\begin{aligned} \Vert \mathcal {F}\big (U\big )(\partial _xu_2-\partial _xv_2)\Vert _{{L^{\frac{p}{2\gamma }}}(\Gamma )}^{\frac{p}{2\gamma }}&\le \Vert \mathcal {F}(U)\Vert _{L^{{p}/{\gamma }}(\Gamma )}^{\frac{p}{2\gamma }}\Vert \partial _xu_2-\partial _xv_2\Vert ^{\frac{p}{2\gamma }}_{L^{{p}/{\gamma }}(\Gamma )}\nonumber \\&\le C\left( 1+\Vert U\Vert _{(L^p(\Gamma ))^2}^{\frac{p}{2\gamma }}\right) \Vert u_2-v_2\Vert ^{\frac{p}{2\gamma }}_{\widehat{W}^{1, p}(\Gamma )}, \end{aligned}$$

where we used Proposition B.1 item (2) and $p/\gamma \le p$.

The inequality (B.34) follows from (B.33) and (1.25).

The inequality (B.35) follows from (B.33) and (1.23).

To prove (B.36) we write

B.41 $$\begin{aligned}&\Vert e^{(\Delta -\sigma I)t}\partial _x \mathcal {H}(U)\Vert _{\mathcal {X}^{\beta }_p}= \Vert e^{\frac{1}{2}(\Delta -\sigma I)t}e^{\frac{1}{2}(\Delta -\sigma I)t}\partial _x \mathcal {H}(U)\Vert _{\mathcal {X}^{\beta }_p}\nonumber \\&\quad \underset{(\hbox {A.10})}{\le }Ce^{-\frac{\delta t}{2}} t^{-\beta }\Vert e^{\frac{1}{2}(\Delta -\sigma I)t}\partial _x \mathcal {H}(U)\Vert _{L^p(\Gamma )}\underset{(\hbox {B.34})}{\le }C e^{-\delta t}t^{-\beta -\frac{p+2\gamma -1}{2p}}\Vert U\Vert _{{L^p(\Gamma )\times \widehat{W}^{1, p}(\Gamma )}}. \end{aligned}$$

The inequality (B.37) is proved analogously by using (A.11).

The last statement follows from Corollary 2.1. $\square$

## General theory of semi-linear parabolic equations on graphs

In this section, we discuss local existence of classical solutions for

C.1 $$\begin{aligned}&u_t=u_{xx}+f(t,x,u, u_x),\quad x\in \Gamma , \end{aligned}$$

C.2 $$\begin{aligned}&\sum \limits _{\vartheta \sim e} \partial _{\nu }u_e(\vartheta )=0,\ u_e(\vartheta )=u_{e'}(\vartheta ), e\sim \vartheta , e'\sim \vartheta ,\end{aligned}$$

C.3 $$\begin{aligned}&u{ (t_0,\cdot )}=u_0(\cdot )\in \mathcal {X}^{\alpha }_p, p\in [1,\infty ). \end{aligned}$$

To that end, we first focus on local existence of strong solutions for

C.4 $$\begin{aligned} u_t=\Delta u+F(t,u), u(t_0)=u_0\in \mathcal {X}^{\alpha }_p, \end{aligned}$$

where $\Delta =\mathcal {A}_p$, cf. (2.1), and $\alpha \ge 0$.

### Definition C.1

[25, Definition 3.3.1] A strong solution of (C.4) on $$[t_0,t_1)$$ with initial condition $$u(t_0)=u_0\in \mathcal {X}_p^\alpha $$ is a continuous function $$u: [t_0, t_1) \rightarrow L^p(\Gamma )$$ such that $$u(t_0)=u_0$$, $$u(t) \in \operatorname {dom}(\mathcal {A}_p)$$ for $$t\in (t_0,t_1)$$, $$\frac{d u}{d t}$$ exists for $$t\in (t_0,t_1)$$, $$(t_0,t_1)\ni t \rightarrow F(t,u(t))\in L^p(\Gamma )$$ is locally Hölder continuous, and $$\int ^{t_0+\sigma }_{t_0} \Vert F(t,u(t))\Vert _{L^p(\Gamma )} dt < \infty $$ for some $$\sigma >0,$$ and the differential equation $$u_t+A_p u=F(t,u) $$ is satisfied on $$(t_0,t_1)$$ in $$L^p(\Gamma )$$.

In the following theorem we adopt classical results regarding strong solutions of (C.4) to the setting of metric graphs.

### Theorem C.1

Let $p\in [1,\infty )$ and $\alpha \in { [}0,1)$. Assume that $F:[0,\infty )\times X_p^\alpha \rightarrow L^p(\Gamma )$ is locally Hölder continuous in *t* with exponent $\nu \in (0,1)$ and locally Lipschitz continuous in *u*, that is, for every $(t, u)\in [0,\infty )\times \mathcal {X}^{\alpha }_p$ there exists a neighborhood $V\subset [0,\infty )\times \mathcal {X}^{\alpha }_p$ containing (*t*, *u*) and a constant $L=L(V)$ such that

C.5 $$\begin{aligned} \Vert F(t_1, u_1)-F(t_2, u_2)\Vert _{L^p(\Gamma )}\le L(|t_1-t_2|^{\nu }+\Vert u_1-u_2\Vert _{\mathcal {X}^{\alpha }_p}), (t_k, u_k)\in V, k=1,2. \end{aligned}$$

Then the following assertions hold.

1. For any $t_0\in [0,\infty )$ and $u_0\in \mathcal {X}_p^\alpha$, there exists $T_{\max }=T_{\max }(t_0,u_0)>0$ such that (C.4) has a unique strong solution $u(t;t_0,u_0)$ on $[t_0,t_0+T_{\max })$ with initial value $u(t_0;t_0,u_0)=u_0.$ Moreover,
  C.6 $$\begin{aligned} u(\cdot ;t_0,u_0)\in C([t_0,t_0+T_{\max }),\mathcal {X}_p^\alpha ), \end{aligned}$$
  and if $T_{\max }< \infty ,$ then
  C.7 $$\begin{aligned} \limsup _{t \nearrow T_{\max }} \left\| u(t+t_0;t_0,u_0) \right\| _{{X_p^{\alpha }}} =\infty . \end{aligned}$$
2. For any $$t_0\in [0,\infty )$$ and $$u_0\in \mathcal {X}_p^\alpha $$, if *u*(*t*) is a strong solution of (C.4) on $$[t_0,t_1)$$ with initial condition $$u(t_0)=u_0$$, then *u* satisfy the following integral equation
  C.8 $$\begin{aligned} u(t)=e^{\Delta ( t-t_0)}u_0 +\int _{t_0}^te^{\Delta ( t-s)} F(s,u(s))ds. \end{aligned}$$
  Conversely, if $t\mapsto u(t)$ is continuous as a mapping from $(t_0,t_1)$ into $X^\alpha ,$ one has $\int ^{t_0+\rho }_{t_0} \Vert F(t,u(t))\Vert _{L^p(\Gamma )}dt < \infty$ for some $\rho >0,$ and if the integral equation (C.8) holds for $t_0< t<t_1,$ then *u*(*t*) is a strong solution of the differential equation (C.4) on $(t_0,t_1).$ Furthermore,
  C.9 $$\begin{aligned} u\in C^\delta ((t_0,T_{\max }), \mathcal {X}_p^\alpha ), \delta \in (0, 1-\alpha ). \end{aligned}$$
3. For any $t_0\in \mathbb {R}$ and $u_0\in X_p^\alpha$,
  C.10 $$\begin{aligned} u(\cdot ;t_0,u_0)\in C^1((t_0,t_0+T_{\max }(t_0,u_0)), \mathcal {X}_p^\beta ), \beta \in (0, \nu ). \end{aligned}$$
4. If $u_n,u_0\in \mathcal {X}_p^\alpha$ and $\Vert u_n-u_0\Vert _{\mathcal {X}_p^\alpha }\rightarrow 0$ as $n\rightarrow \infty$, then for any $t_0\in \mathbb {R}$, one has
  C.11 $$\begin{aligned} \liminf _{n\rightarrow \infty } T_{\max }(t_0,u_n)\ge T_{\max }(t_0,u_0), \end{aligned}$$
  and
  C.12 $$\begin{aligned} \lim _{n\rightarrow \infty }\Vert u(t;t_0,u_n)-u(t;t_0,u_0)\Vert _{\mathcal {X}_p^\alpha }=0, \end{aligned}$$
  uniformly in compact intervals of $[t_0,t_0+T_{\max }(t_0,u_0))$.

### Proof

(1) It follows from [25, Theorem 3.3.3 ] and [25, Theorem 3.3.4 ] together with the fact that $e^{t\Delta }$ is analytic in $L^p(\Gamma )$.

(2) The first statement in part (2) follows from [25, Lemma 3.3.2], the fact that $u \in C^\delta ((t_0,t_1);X^\alpha )$ is obtained in the course of the proof of [25, Lemma 3.3.2].

(3) The proof of part (3) stems from that of [25, Theorem 3.5.2]. For completeness, we provide the details. It suffices to prove

$$\begin{aligned} u(\cdot ;t_0,u_0)\in C^1((\tau ,\tau +h),\mathcal {X}_p^\beta ), t_0<\tau<\tau +h<t_0+T_{\max }(t_0,u_0),\,\,0<\beta <\nu , \end{aligned}$$

for all $\tau \in (t_0, t_0+T_{\max }(t_0, u_0))$ and some $h=h(\tau )>0$. For any fixed $\tau \in (t_0, T_{\max }(t_0,u_0))$, let $u_1=u(\tau ;t_0,u_0)$. Then one has $u(t;t_0,u_0)=u(t;\tau ,u_1),\ \tau<t<T_{\max }(t_0,u_0)$ and for any $\sigma >0$,

C.13 $$\begin{aligned} u(t;t_0,u_0)=u(t;\tau ,u_1)={ e^{(\Delta -\sigma )(t-\tau )}u_1+\int _{\tau }^t e^{(\Delta -\sigma )(t-s)}g(s)ds,} \end{aligned}$$

where $g(t){:}{=}{ u(t;\tau ,u_1)}+F(t,u(t;\tau ,u_1))$. By  [25, Lemma 3.5.1], in order to prove that the mapping

C.14 $$\begin{aligned} (\tau , \tau +h)\ni t\mapsto G(t){:}{=}\int _{\tau }^t e^{(\Delta -\sigma )(t-s)}g(s)ds \end{aligned}$$

is continuously differentiable into $\mathcal {X}^{\beta }_p$ for some $h=h(\tau )>0$, hence, $u(\cdot , t_0, u_0)\in C^1((\tau ,\tau +h),\mathcal {X}_p^\beta )$ it suffices to show that

C.15 $$\begin{aligned} \Vert g(s)-g(t)\Vert _{L^p(\Gamma )}\le K(t) (s-t)^{\nu },\ \tau<t<s<\tau +h, \end{aligned}$$

for some continuous $K(\cdot )$ and $\int _{\tau }^{\tau +h}K(t)dt<\infty$. Our objective, therefore, is to derive (C.15). To that end, by (C.5), there exist a neighborhood $V\subset [0,\infty )\times \mathcal {X}^{\alpha }_p$ containing $(\tau ,u_1)$, and positive constants $L>0$, $h>0$ such that

C.16 $$\begin{aligned} \Vert g(t)-g(s)\Vert _{L^p(\Gamma )}\le L\big (|t-s|^\nu +\Vert u(t;\tau ,u_0)-u(s;\tau ,u_0)\Vert _{X_p^\alpha }\big ), \end{aligned}$$

for $s,t\in (\tau , \tau +h)$ and $t<s$. Note that

$$\begin{aligned} u(s;\tau ,u_1)-u(t;\tau ,u_1)&=\big (e^{(\Delta -\sigma ) (s-t)}-I\big )e^{(\Delta -\sigma )(t-\tau )}u_1\\&\quad +\int _{\tau }^t e^{(\Delta -\sigma )(t-\kappa )}\big (g(\kappa +s-t)-g(\kappa )\big )d\kappa \\&\quad +\int _{\tau }^{\tau +s-t} e^{(\sigma -\Delta )(s-\kappa )}g(\kappa )d\kappa . \end{aligned}$$

Then there exist constants $C_1, C_2>0$ such that

$$\begin{aligned}&\Vert u(s;\tau ,u_1)-u(t;\tau ,u_1)\Vert _{\mathcal {X}_p^\alpha }\\&\quad \le C_1\big ( (s-t)\cdot \Vert (\sigma -\Delta )^{1+\alpha } e^{(\Delta -\sigma )(t-\tau )} { u_1}\Vert _{L^p(\Gamma )} \\&\qquad +\int _{\tau }^t (t-\kappa )^{-\alpha } \Vert g(\kappa +s-t)-g(\kappa )\Vert _{L^p(\Gamma )}d\kappa \\&\qquad +\int _{\tau }^{\tau +s-t}\Vert (\sigma -\Delta )^\alpha e^{\Delta (s-\kappa )} g(\kappa )\Vert _{L^p(\Gamma )}d\kappa \big )\\&\quad \le C_2\big ((s-t) (t-\tau )^{-\alpha }\Vert { (\sigma -\Delta ) u_1}\Vert _{L^p(\Gamma )}+ \int _{\tau }^t (t-\kappa )^{-\alpha }\Vert g(\kappa +s-t)-g(\kappa )\Vert _{L^p(\Gamma )}d\kappa \\&\qquad +(s-t)(t-\tau )^{-\alpha } \big ). \end{aligned}$$

This together with (C.16) imply

C.17 $$\begin{aligned} \Vert g(s)-g(t)\Vert _{L^p(\Gamma )}&\le L (s-t)^\nu + C_2\big ((s-t) (t-\tau )^{-\alpha }\Vert { (\sigma -\Delta )u_1}\Vert _{L^p(\Gamma )}+ (s-t) (t-\tau )^{-\alpha }\big )\nonumber \\&\quad +C_2\int _{\tau }^t (t-\kappa )^{-\alpha }\Vert g(k+s-t)-g(\kappa )\Vert _{L^p(\Gamma )}d\kappa . \end{aligned}$$

Then Gronwall’s inequality (see [25, Section 1.2.1] yields $L_1>0$ such that

C.18 $$\begin{aligned} \Vert g(s)-g(t)\Vert _{L^p(\Gamma )}\le (s-t)^{\nu } K(t), \end{aligned}$$

where

$$\begin{aligned} K(t){:}{=}L_1 \Big ( 1+ { h}^{1-\nu } (t-\tau )^{-\alpha }\Vert { (\sigma -\Delta )u_1}\Vert _{L^p(\Gamma )}+ { h}^{1-\nu }(t-\tau )^{-\alpha }\Big ), \end{aligned}$$

as required in (C.15).

(4) Follows from [25, Theorem 3.4.1]. $\square$

Equation (C.1) is related to (C.4) via the following elementary proposition.

### Lemma C.1

Let $f:[0,\infty )\times \Gamma \times {\mathbb {R}}\times {\mathbb {R}}\rightarrow {\mathbb {R}}$. Assume that there exists $\nu \in (0,1)$, such that for every triple $(t,u,v)\in [0,\infty )\times {\mathbb {R}}\times {\mathbb {R}}$ there exists an open neighborhood $U\subset [0,\infty )\times {\mathbb {R}}\times {\mathbb {R}}$ and a constant $L=L(U)$ such that for all $(t_k, u_k, v_k)\in U$, $k=1,2$ one has

C.19 $$\begin{aligned} |f(t_1, x, u_1, v_1)-f(t_2, x, u_2, v_2)|\le L(|t_1-t_2|^{\nu }+|u_1-u_2|+|v_1-v_2|), \end{aligned}$$

that is, $f=f(t,x,u,v)$ is locally Hölder continuous in *t* with exponent $\nu \in (0,1)$, locally Lipschitz continuous in *u* and *v*, uniformly for $x\in \Gamma$. Assume in addition that $f(t, \cdot , u,v)\in L^p(\Gamma )$. Let $p\in [1,\infty )$ and $\alpha \in (0,1)$ be such that $2\alpha -p^{-1}>1$. For $u\in \mathcal {X}^{\alpha }_p$ let $F(t,u)=f(t,\cdot ,u,u_x)$ then the mapping $F:[0,\infty )\times \mathcal {X}_p^\alpha \rightarrow L^p(\Gamma )$ is locally Hölder continuous in *t* with exponent $\nu$ and Lipschitz continuous in *u*.

### Proof

Fix $(t_0, u_0)\in [0,\infty )\times \mathcal {X}^{\alpha }_p$. Then due to $2\alpha -\frac{1}{p}>1$, one has $X_p^\alpha \hookrightarrow \widehat{C}^1(\widehat{\Gamma })$, see (A.9). Therefore we may choose an open neighborhood $V\subset [0,\infty )\times \mathcal {X}^{\alpha }_p$ containing $(t_0, u_0)$ such that for $(t_k, u_k)\in V$, $k=1,2$, we have $(t_k, u_k(x), \partial _xu_k(x))\in U$ for all $x\in \Gamma$. Then by (C.19) we have

C.20 $$\begin{aligned}&|f(t,x,u(x),u_x(x))-f(s,x,v(x),v_x(x))|\nonumber \\&\quad \le L \big (|s-t|^\nu +|u(x)-v(x)|+|u_x(x)-v_x(x)|,\ x\in \Gamma . \end{aligned}$$

Hence, $$f(t,\cdot ,u(\cdot ),u_x(\cdot ))-f(s,\cdot ,v(\cdot ),v_x(\cdot ))\in L^p(\Gamma )$$ and

$$\begin{aligned}&\Vert f(t,\cdot ,u(\cdot ),u_x(\cdot ))-f(s,\cdot ,v(\cdot ),v_x(\cdot ))\Vert _{L^p(\Gamma )}\\&\quad \le L \big (|t-s|^\nu \sum _{e\in \mathcal {E}}|e|^{\frac{1}{p}} +\Vert u(\cdot )-v(\cdot )\Vert _{L^p(\Gamma )}+\Vert u_x(\cdot )-v_x(\cdot )\Vert _{L^p(\Gamma )}\big )\\&\quad \le {\tilde{L}} \big (|t-s|^\nu +\Vert u-v\Vert _{X_p^\alpha } \big ), \end{aligned}$$

as required. $$\square $$

Applying the above theorem, we have

### Theorem C.2

Assume that $f=f(t,x,u,v)$ is as in Lemma C.1 and suppose in addition that $f(t, \cdot , u, v)\in \widehat{C}(\overline{\Gamma })$. Assume that $0<\alpha <1$ and $1<p<\infty$ are such that $2\alpha -p^{-1}>1$ and $2\nu -p^{-1}>0$. Then the following assertions hold.

(1) (Existence of classical solutions) For given $$t_0\in {\mathbb {R}}$$ and $$u_0\in \mathcal {X}_p^\alpha $$, (C.1) has a unique classical solution $$u(t,x;t_0,u_0)$$ on some interval $$[t_0,t_0+T)$$ with initial condition $$u(t_0,x;t_0,u_0)=u_0(x)$$ in $$\mathcal {X}_p^\alpha $$, and

C.21 $$\begin{aligned} u(\cdot ,\cdot ;t_0,u_0)\in C([t_0,t_0+T_{\max }),\mathcal {X}_p^\alpha )\cap C^\delta ((t_0,t_0+T_{\max }),\mathcal {X}_p^\alpha )\cap C^1((t_0,t_0+T_{\max },\mathcal {X}_p^\beta ) \end{aligned}$$

for all $\delta \in (0,1-\alpha )$ and $\beta \in (0,\nu )$. Moreover if $T_{\max }< \infty ,$ then

C.22 $$\begin{aligned} \limsup _{t \rightarrow T^-_{\max }} \left\| u(t+t_0,\cdot ;t_0,u_0) \right\| _{\mathcal {X}_p^\alpha }=\infty . \end{aligned}$$

(2) (Continuity with respect to initial conditions) If $u_n,u_0\in \mathcal {X}_p^\alpha$ and $\Vert u_n-u_0\Vert _{\mathcal {X}_p^\alpha }\rightarrow 0$ as $n\rightarrow \infty$, then for any $t_0\in \mathbb {R}$, $\liminf _{n\rightarrow \infty } T_{\max }(t_0,u_n)\ge T_{\max }(t_0,u_0)$ and

$$\begin{aligned} \lim _{n\rightarrow \infty }\Vert u(t,\cdot ;t_0,u_n)-u(t,\cdot ;t_0,u_0)\Vert _{\mathcal {X}_p^\alpha }=0 \end{aligned}$$

uniformly in compact intervals of $[t_0,t_0+T_{\max }(t_0,u_0))$.

(3) (Comparison principle) If $u_1, u_2\in \widehat{C}(\overline{\Gamma })$ and $u_1(x)\le u_2(x)$, $x\in \Gamma$ then $u(t,x;t_0,u_1)\le u(t,x;t_0,u_2)$, $x\in \Gamma$ for $t>t_0$ such that both $u(t,x;t_0,u_1)$ and $u(t,x;t_0,u_2)$ exist.

### Proof of Theorem C.2

(1) First, let us define a mapping $F:[0,\infty )\times \mathcal {X}^{\alpha }_p\rightarrow L^p(\Gamma )$ as follows

C.23 $$\begin{aligned} F(t,u)(x)=f(t,x,u(x),u_x(x)), u\in \mathcal {X}^{\alpha }_p. \end{aligned}$$

Then by Lemma C.1$F=F(t,u)$ satisfies the hypotheses of Theorem C.1. Therefore for every $u_{0}\in \mathcal {X}^{\alpha }_p$ there exists a unique strong solution $u(t;t_0,u_0)$ of (C.4) defined on the maximal time interval $(t_0, T(t_0, u_0))$ and satisfying the initial condition $u(t_0;t_0,u_0)=u_0$. Let us denote

C.24 $$\begin{aligned} u(t, x; t_0, u_0){:}{=}u(t; t_0, u_0)(x), x\in \Gamma . \end{aligned}$$

Then (C.21) follows from (C.6), (C.9) and (C.10), while (C.22) follows from (C.16).

Next, we prove that $u(t,x;t_0,u_0)$ is a classical solution of (C.1). To that end we note that $2\alpha -p^{-1}>1$ and $2\nu -p^{-1}>0$. Then there is $\beta \in (0,\nu )$ such that $2\beta -p^{-1}>0$, hence, by (A.9) one has

$$\begin{aligned} \mathcal {X}^{\alpha }_p\hookrightarrow \widehat{C}^{1}({\overline{\Gamma }}),\quad \mathcal {X}_p^\beta \hookrightarrow \widehat{C}({\overline{\Gamma }}). \end{aligned}$$

This together with (C.6) and (C.10) yield

C.25 $$\begin{aligned} u(\cdot ,\cdot ;t_0,u_0)\in \widehat{C}^{0,1}([t_0,t_0+T_{\max }(t_0,u_0))\times {\overline{\Gamma }})\cap \widehat{C}^{1,0}((t_0,t_0+T_{\max }(t_0,u_0))\times {\overline{\Gamma }}). \end{aligned}$$

Next, defining

$$\begin{aligned} {\tilde{f}}(t,x)=f(t,x,u(t,x;t_0,u_0),u_x(t,x;t_0,u_0)). \end{aligned}$$

and using (C.25) one obtains

C.26 $$\begin{aligned} {\tilde{f}}\in \widehat{C}^{0,0}((t_0,t_0+T_{\max }(t_0,u_0))\times {\overline{\Gamma }}), \end{aligned}$$

Re-writing (C.1) we obtain

$$\begin{aligned} -u_{xx}(t,x;t_0,u_0)=-u_t(t,x;t_0,u_0)+{\tilde{f}}(t,x). \end{aligned}$$

This together with (C.25) and (C.26) yield

$$\begin{aligned} u(\cdot ,\cdot ;t_0,u_0)\in \widehat{C}^{1,2}((t_0,t_0+T_{\max }(t_0,u_0))\times {\overline{\Gamma }}). \end{aligned}$$

Therefore, $u(t,x;t_0,u_0)$ is a classical solution of (C.1) with initial condition $u(t_0,x;t_0,u_0)=u_0(x)\in \mathcal {X}_p^\alpha$.

Now, we claim that (C.1) has a unique classical solution $u(t,x;t_0,u_0)$ satisfying $u(t_0,x;t_0,u_0)=u_0(x)$ and (C.21). In fact, if $u(t,x;t_0,u_0)$ is a classical solution of (C.1) satisfying $u(t_0,x;t_0,u_0)=u_0(x)$ and (C.21), then $u(t,\cdot ;t_0,u_0)$ satisfies

$$\begin{aligned} u(t,\cdot ;t_0,u_0)=e^{\Delta ( t-t_0)}u_0(\cdot ) +\int _{t_0}^te^{\Delta ( t-s)} F(s,u(s,\cdot ;t_0,u_0))ds, \end{aligned}$$

and hence is a strong solution of (C.4) with initial condition $u(t_0,\cdot ;t_0,u_0)=u_0(\cdot )$. The claim then follows from the uniqueness of strong solutions of (C.4).

(2) Follows from Theorem C.1 (4).

(3) We divide the proof into 3 steps.

**Step 1**. Let us introduce the following notation

C.27 $$\begin{aligned}&w_0{:}{=}u_2-u_1, w(t,x){:}{=}u(t,x;t_0,u_2)-u(t,x;t_0,u_1), \end{aligned}$$

C.28 $$\begin{aligned}&U_k{:}{=}u(t,x;t_0,u_k), V_k{:}{=}\partial _x U_k, k=1,2. \end{aligned}$$

Then by assumption $w_0\ge 0$ and one has

C.29 $$\begin{aligned}&-w_t+w_{xx}+f(t,x,U_1,V_1) -f(t,x,U_2,V_2)=0, \end{aligned}$$

C.30 $$\begin{aligned}&\sum \limits _{\vartheta \sim e} \partial _{\nu }w_e(\vartheta )=0, \end{aligned}$$

For given $U_1=u(t,x;t_0,u_1)$ and $U_2=u(t,x;t_0,u_2)$ we will rewrite (C.29) as a linear parabolic equation for *w*(*x*, *t*). To that end, let

$$\begin{aligned} \Phi (x,t,s){:}{=}f\left( t,x,sU_1+(1-s)U_2,sV_1+(1-s)V_2\right) . \end{aligned}$$

Then one has

C.31 $$\begin{aligned} \Phi (x,t,1)-\Phi (x,t,0)=f(t,x,U_1,(U_1)_{x}) -f(t,x,U_2,(U_2)_{x}), \end{aligned}$$

and

$$\begin{aligned} \Phi (x,t,1)-\Phi (x,t,0)&=\int _0^1 \frac{\partial \Phi }{\partial s}(t,x,s)ds \\&=\int _0^1 \frac{\partial f}{\partial u}(t,x,sU_1+(1-s)U_2,sV_1+(1-s)V_2)(U_1-U_2)ds \\  &\quad + \int _0^1 \frac{\partial f}{\partial v}(t,x,sU_1+(1-s)U_2,sV_1+(1-s)V_2)(V_1-V_2) ds \\  &= B(x,t)(U_1-U_2) + C(x,t)(V_1-V_2), \end{aligned}$$

where we denoted

$$\begin{aligned} B(x,t)&=\int _0^1 \frac{\partial f}{\partial u}(t,x,sU_1+(1-s)U_2,sV_1+(1-s)V_2)ds \quad \text { and } \\ C(x,t)&=\int _0^1 \frac{\partial f}{\partial v}(t,x,sU_1+(1-s)U_2,sV_1+(1-s)V_2) ds \end{aligned}$$

Therefore (C.29) can be rewritten as follows

$$\begin{aligned} L(w){:}{=}-w_t+w_{xx}+B(x,t)w_x+C(x,t)w=0. \end{aligned}$$

**Step 2**. Let us define $\widehat{w}(x,t)$via $w(x,t) = e^{-\lambda t}\hat{w}(x,t)$, where $\lambda = \sup _{x,t}|C(x,t)|$. Then one has

$$\begin{aligned} L(w)&= -( \widehat{w}_t e^{-\lambda t} - \lambda \widehat{w} e^{-\lambda t}) +\widehat{w}_{xx} e^{-\lambda t} + B(x,t)\widehat{w}_x e^{-\lambda t} + \widehat{w} e^{-\lambda t} \\&= e^{-\lambda t} \left( - \widehat{w}_t +\widehat{w}_{xx} + B(x,t)\widehat{w}_x + (C(x,t) + \lambda )\widehat{w} \right) =0. \end{aligned}$$

Thus $\hat{w}$ satisfies $\hat{L}(\widehat{w})=0$ where $\widehat{L}$ is defined as

C.32 $$\begin{aligned} \widehat{L}(\widehat{w})= - \widehat{w}_t +\widehat{w}_{xx} + B(x,t)\widehat{w}_x + \widehat{C}(x,t) \widehat{w}, \text { where } \widehat{C}(x,t)= C(x,t) + \lambda \ge 0 \end{aligned}$$

**Step 3**. In this step we show that for any $u_1,u_2 \in \mathcal {X}_p^\alpha$ with $u_2-u_1 \ge 0$ we have $u(t,x;t_0,u_2)-u(t,x;t_0,u_1)\ge 0$, $t\ge t_0$

First, assume that $u_1,u_2 \in C({\overline{\Gamma }})\cap \mathcal {X}_p^\alpha$ and $\inf _{x\in \Gamma }(u_2-u_1)(x)>0$. Then by definition $\widehat{w}_0 \in C({\overline{\Gamma }})\cap \mathcal {X}_p^\alpha$ and $\inf _{x\in \Gamma }\widehat{w}_0(x)=\inf _{x\in \Gamma }e^{\lambda t}(u_2-u_1)(x)>0$. We claim that $\widehat{w}(t,x;\hat{w}_0)>0$ for all $t>t_0$ and $x\in \Gamma$. Assuming the contrary, there exist $t_1>t_0$ and $x_1\in \Gamma$ such that $\widehat{w}(t_1,x_1;t_0,\widehat{w}_0)=0$ and $\widehat{w}(t,x;t_0,\widehat{w}_0)>0$ for all $t_0<t<t_1$ and $x\in \Gamma$. Let $e_1\in \mathcal {E}$ be such that $x_1\in {\bar{e}}_1$. Using the maximum principle for parabolic equations (on bounded intervals) we infer that $x_1$ is an end point of edge $e_1$, that is, $x_1\in \partial e_1$. Then Hopf’s Lemma gives

$$\begin{aligned} \partial _{\nu } \widehat{w_e}(t_1,x_1;t_0,\hat{w}_0)< 0,\ \text { for every edge }\,\, e \in \mathcal {E}\, \textrm{with}\,\, x_1\in \partial e, \end{aligned}$$

where $\frac{\partial \widehat{w}}{\partial \nu }$, as usual, denotes the derivative of $\widehat{w}$ with respect to the unit outer normal. This implies, in particular, that

$$\begin{aligned} \sum _{e\sim x_1}{\partial _{\nu } \widehat{w}_e(x_1)} < 0. \end{aligned}$$

This contradicts the vertex condition (C.30). Thus $u(t,x;t_0,u_2)-u(t,x;t_0,u_1)\ge 0$ as asserted.

Next, for any $u_1,u_2\in \mathcal {X}_p^\alpha$, due to $2\alpha -p^{-1}>0$ and (A.9), there exist $u_{1,n},u_{2, n}\in C({\overline{\Gamma }})\cap \mathcal {X}_p^\alpha$ such that

$$\begin{aligned} \lim \limits _{n\rightarrow \infty }(u_{2, n}-u_{1, n})= u_2-u_1\quad \textrm{in}\quad L^p(\Gamma ). \end{aligned}$$

Moreover, since $\inf _{x\in \Gamma }(u_2-u_1)\ge 0$ we may assume that $\inf _{x\in \Gamma }(u_{2,n}-u_{1,n})>0$. Therefore one has

C.33 $$\begin{aligned} \widehat{w}_n{:}{=}e^{\lambda t}(u_{2, n}-u_{1, n})\in C({\overline{\Gamma }})\cap \mathcal {X}_p^\alpha ,\ \inf _{x\in \Gamma } \widehat{w}_n(x)>0, \widehat{w}_n\rightarrow \widehat{w}\text { in } L^p(\Gamma ), \end{aligned}$$

and, by the previous argument,

C.34 $$\begin{aligned} \widehat{w}(t,x; t_0, \hat{w}_n)>0, t>t_0, x\in \Gamma , n\ge 1. \end{aligned}$$

Finally, using

C.35 $$\begin{aligned} \widehat{w}(t,\cdot ;t_0, \hat{w}_n)\rightarrow \widehat{w}(t,\cdot ;t_0,\hat{w}_n)\quad \textrm{as}\quad n\rightarrow \infty \quad \textrm{in}\quad L^p(\Gamma ), \end{aligned}$$

we obtain $u(t,x;t_0,u_2)-u(t,x;t_0,u_1)\ge 0$, $t\ge t_0$ as required. $\square$

### Proposition C.1

[25, p. 190]. Let $\alpha \in [0,1)$ $a_1, a_2>0$, $T>0$. Assume that an integrable function $u:[0,T]\rightarrow [0,\infty )$ satisfies

C.36 $$\begin{aligned} 0\le u(t)\le a_1 t^{-\alpha }+a_2 \int _0^t(t-s)^{-\alpha }u(s)ds, \end{aligned}$$

for $t\in [0,T]$. Then there exists $C=C(\alpha , a_2, T)>0$ such that

C.37 $$\begin{aligned} 0\le u(t)\le \frac{a_1C}{1-\alpha } t^{-\alpha }, \ t\in (0, T). \end{aligned}$$

## Footnotes

1 A more comprehensive introduction into differential operators and their spectral theory on metric graphs can be found, for example, in [6].

2 See Sect. 4.1 for a more detailed outline of the proof.

3 See Sect. 5.1 for a more detailed outline of the proof.

4 $\mathcal {B}(\mathcal {X}, \mathcal {Y})$ denotes the space of bounded linear operators between Banach spaces $\mathcal {X}, \mathcal {Y}$.

5 In the setting of compact graphs the constant *C* must be *T*-dependent. Indeed, $u\equiv 1$ does not satisfy the inequality with any *T*-independent *C* for $p<q$. However, Corollary 2.1 does provide some variations of (1.21)–(1.26) the operator $e^{(\Delta -\sigma )t}$ with $\sigma \gg 1$ and time-independent constant *C*.

6 Which is not unique, as any fixed value of $R>(2\ell _+^2 + 8T\log {(\deg )}+1)\ell _-^{-2}$ will serve the same purpose.

7 In general, $$\delta \ll 1$$

8 We also invoke [26, Cor. 3.3.5] to get solution $\Psi$ not just on a small interval near $\tau$ but on $[\tau , T)$.

9 The first embedding holds for $\beta >\frac{1}{2p}$. Since $p\ge 8$, the first embedding holds for $\beta \in (\frac{1}{16}, \frac{1}{8})$. Throughout the rest of the proof we assume this range for $\beta$ without loss of generality.

10 For brevity we will abbreviate the maximal time of existence in both cases $\tau =0$ and $\tau >0$ by $T_{\max }$ instead of $T_{\max }(u_0)$ or $T_{\max }(u_0, v_0)$.
